# High-frequency irreversible electroporation versus transurethral resection of the prostate for benign prostatic hyperplasia (GIANT): a single-centre, randomised, double-blind, phase 3, non-inferiority trial

**DOI:** 10.1016/j.eclinm.2026.104034

**Published:** 2026-07-02

**Authors:** Bi-Ming He, Rong-Bing Li, Dong-Yang Li, Li-Qun Huang, Zhi-Chao Jin, Zhen-Kai Shi, Shuai-Dong Wang, Jia-Sun Lu, Ji-Ling Wen, Hai-Feng Wang

**Affiliations:** a Department of Urology, Shanghai East Hospital, School of Medicine, Tongji University, Shanghai, China; b Department of Urology, Shanghai Geriatric Medical Center, Zhongshan Hospital Fudan University Minhang Campus, Shanghai, China; c Department of Health Statistics, Naval Medical University, Shanghai, China; d Department of Urology, Jing'an District Central Hospital, Fudan University, Shanghai, China

**Keywords:** Benign prostatic hyperplasia, Transurethral resection of the prostate, Irreversible electroporation, Non-thermal ablation, Ejaculatory preservation, Minimally invasive surgical therapies

## Abstract

**Background:**

Transurethral resection of the prostate (TURP) is the gold standard for treating benign prostatic hyperplasia (BPH) but frequently causes retrograde ejaculation and bleeding. High-frequency irreversible electroporation (H-FIRE) is a non-thermal ablation technique offering tissue selectivity. We aimed to compare the efficacy and safety of H-FIRE versus TURP in men with symptomatic BPH.

**Methods:**

In this investigator-initiated, single-centre, randomised, double-blind, non-inferiority trial at Shanghai East Hospital, China, men aged 40 years or older with symptomatic BPH (prostate volume 30–100 mL) were assigned (1:1) to undergo H-FIRE or TURP. To maintain blinding, a sham protocol involving perineal dressings and standardised irrigation was used. The co-primary outcomes were change from baseline in maximum urinary flow rate (Qmax) and International Prostate Symptom Score (IPSS) at 3 months. Non-inferiority margins were −4 mL/s for Qmax and 3 points for IPSS. Efficacy was analysed in the intention-to-treat population. This trial is registered with ClinicalTrials.gov, NCT05306145.

**Findings:**

Between June 30, 2022, and May 25, 2025, 118 participants were enrolled and randomly assigned to H-FIRE (n = 59) or TURP (n = 59). H-FIRE was non-inferior to TURP for both primary endpoints: mean Qmax improvement was 7.95 mL/s with H-FIRE versus 7.84 mL/s with TURP (adjusted difference 0.11 mL/s, 95% CI −2.52 to 2.73; p = 0.001 for non-inferiority); mean IPSS reduction was −13.24 versus −13.75 (adjusted difference 0.50, −1.09 to 2.10; p = 0.001 for non-inferiority). Retrograde ejaculation occurred in 0% (0/59) of patients in the H-FIRE group compared with 54.2% (32/59) in the TURP group (p < 0.0001). H-FIRE was associated with greater haemodynamic stability (mean haemoglobin change +0.82 g/L [95% CI −1.84 to 3.48] versus −12.14 g/L [−14.72 to −9.55]; p < 0.0001) but required longer postoperative catheterisation (median 19 days [IQR 15.0–22.0] versus 4 days [4.0–5.0]). There were no grade 3–4 adverse events, and no deaths occurred in either group.

**Interpretation:**

At 3 months, H-FIRE provided short-term functional improvement non-inferior to TURP while completely preserving ejaculatory function and minimising blood loss. Although associated with longer temporary catheterisation due to tissue resorption, H-FIRE represents a viable function-sparing alternative for men prioritising sexual health and offers potential for ambulatory management.

**Funding:**

10.13039/501100001809National Natural Science Foundation of China (No. 82373048) and 10.13039/501100001809National Natural Science Foundation of China for Youth (No. 82303612).

**Research in context**

**Evidence before this study**

We searched PubMed, Embase, and the Cochrane Library for randomised controlled trials published from inception to May 30, 2025, using the search terms “benign prostatic hyperplasia”, “transurethral resection of the prostate”, “irreversible electroporation”, “minimally invasive surgical therapies”, and “randomised trial”. No language restrictions were applied. Transurethral resection of the prostate (TURP) provides effective symptom relief but is associated with a 65–80% rate of retrograde ejaculation and significant perioperative bleeding. While novel minimally invasive surgical therapies (MISTs) aim to reduce morbidity, most rely on thermal energy, which poses risks to neurovascular bundles. Previous trials comparing MISTs with TURP have largely been limited by open-label designs, insufficient statistical power for non-inferiority, or a failure to demonstrate outcomes comparable to the surgical gold standard. No previous double-blind, sham-controlled randomised trials have compared non-thermal ablation technology with TURP.

**Added value of this study**

To our knowledge, the GIANT trial is the first double-blind, randomised, sham-controlled trial to compare high-frequency irreversible electroporation (H-FIRE) with TURP. By implementing a rigorous sham protocol involving perineal dressings and unified postoperative care, we achieved high internal validity and successfully maintained blinding. We found that H-FIRE was non-inferior to TURP regarding symptom relief and flow rate improvement at 3 months. Crucially, H-FIRE demonstrated a unique “function-sparing” profile with 0% de novo retrograde ejaculation (compared with 54% in the TURP group) and negligible blood loss. These benefits were observed alongside a trade-off: H-FIRE required a longer duration of postoperative catheterisation (median 19 days) due to the mechanism of gradual tissue resorption.

**Implications of all the available evidence**

H-FIRE represents a viable, function-sparing alternative to TURP, offering patients a distinct choice: accepting a temporary period of prolonged catheterisation in exchange for superior haemodynamic stability and the lifelong preservation of ejaculatory function. The safety profile of H-FIRE—characterised by the absence of irrigation requirements and bleeding risks—supports its potential implementation as an ambulatory (day-case) procedure, which could significantly improve health system efficiency by reducing hospital bed occupancy. Future research should focus on optimising ablation parameters to shorten catheterisation time and assessing long-term durability.

## Introduction

Benign prostatic hyperplasia (BPH) is a common condition characterised by the proliferation of smooth muscle and epithelial cells within the prostate gland. It predominantly affects men over the age of 50, with prevalence increasing markedly with age.^1^ BPH frequently leads to bothersome lower urinary tract symptoms (LUTS) and benign prostatic obstruction (BPO), substantially impairing quality of life (QOL) in ageing males and posing a growing public health challenge.^2^ Over the past two decades, the global burden of BPH has risen dramatically and is projected to continue increasing due to population ageing and growth.^3^

When conservative management fails or complications arise, surgical intervention is indicated. Since its introduction in 1970, transurethral resection of the prostate (TURP) has remained the gold standard surgical treatment for LUTS/BPO.^4^ Although generally effective, TURP is associated with several notable complications, including transurethral resection syndrome, significant bleeding, urinary incontinence, and sexual dysfunction.^5^, ^6^, ^7^, ^8^ These adverse events may prolong hospitalisation and increase healthcare costs. Consequently, the evolving landscape of BPH management has witnessed the continuous development of various minimally invasive alternatives—ranging from novel energy ablation techniques to prostatic stents^9^ —which aim to achieve effective de-obstruction while minimising surgical morbidity.

High-frequency irreversible electroporation (H-FIRE) is a non-thermal ablative modality that employs pulsed, high-voltage, low-energy electric currents to create nanoscale pores in cell membranes, leading to cell death.^10^ Unlike thermal ablation, H-FIRE exhibits tissue selectivity, preserving the collagen matrix and critical surrounding structures, such as nerves and blood vessels.^11^^,^^12^ In a previous single-arm trial involving patients with localised prostate cancer, H-FIRE demonstrated favourable urinary functional outcomes with minimal impact on erectile function and urinary continence.^13^ However, evidence regarding its efficacy and safety in treating LUTS/BPO remains limited. Therefore, we initiated the GIANT trial—a non-inferiority, double-blind, randomised controlled study—to test the hypothesis that H-FIRE provides urinary functional outcomes comparable to those of TURP, while resulting in fewer side effects in men with LUTS/BPO.

## Methods

### Study design and participants

The GIANT trial was an investigator-initiated, single-centre, randomised, double-blind, non-inferiority trial conducted at Shanghai East Hospital, China. The study compared H-FIRE with standard TURP in men with LUTS secondary to BPO. The trial adhered to a predefined statistical analysis plan, and results are reported following CONSORT guidelines. An independent Data and Safety Monitoring Board oversaw safety, protocol compliance, and data integrity. All authors vouch for the accuracy and completeness of the data and for the fidelity of the trial to the protocol. The first author and the last author designed the trial, and the first author wrote the manuscript draft.

Eligible participants were adult men with bothersome LUTS/BPO for whom TURP was considered appropriate in standard practice. Inclusion criteria included age ≥40 years, prostate volume between 30 and 100 mL, maximum flow rate (Q_max_) < 15 mL/s, and an International Prostate Symptom Score (IPSS) > 8. Exclusion criteria comprised a history of prostate cancer, suspected malignancy, neurogenic bladder, metallic implants, prolonged catheterisation (>2 weeks), or prior prostate/urethral surgery, or any other conditions deemed unsuitable by the investigator. The full study protocol, including detailed eligibility criteria, has been published previously.^14^ Information regarding race and ethnicity was self-reported by the participants using predefined categories according to local demographic standards.

Patients were not formally involved in the design or conduct of this study. This decision was primarily driven by the logistical challenges of maintaining strict blinding in a sham protocol surgical trial. However, the genesis of the research question was rooted in patient values observed in our clinical practice—specifically, the widespread prioritisation of ejaculatory preservation over maximal flow rate improvement. Regarding dissemination, although not originally specified in the protocol, we have updated our plan to provide a plain language summary of these results to study participants upon request, reflecting our commitment to patient engagement.

### Ethics

The study protocol was approved by the Institutional Ethics Committee of Shanghai East Hospital (Approval/Reference Number: 2021117). The trial was conducted in accordance with the Declaration of Helsinki and Good Clinical Practice guidelines. Written informed consent was obtained from all participants prior to enrolment and any study-related procedures.

### Randomisation and masking

Participants were randomly assigned in a 1:1 ratio to receive either H-FIRE or TURP. Randomisation was performed using blocks of 6 and stratified by age (<70 versus ≥70 years) and prostate volume (<60 mL versus ≥60 mL). An independent computerised system generated the allocation sequence, which was revealed only after written consent and eligibility confirmation.

To ensure rigorous blinding, particularly for patient-reported outcomes (PROs), we implemented a comprehensive masking strategy addressing both physical and psychological aspects. First, to prevent unblinding due to differing side–effect profiles (e.g., prolonged catheterisation in H-FIRE versus haematuria in TURP), a unified preoperative counselling strategy was implemented. All participants were informed of a combined list of potential risks associated with both procedures—including urinary retention, prolonged catheterisation, bleeding, and retrograde ejaculation—without attributing specific risks to a specific arm. This ensured that postoperative symptoms would not definitively unblind the participant.

Physically, postoperative care was strictly standardised: all patients, regardless of group, received continuous bladder irrigation for a minimum of 24 h, discontinued solely based on urine colour. Similarly, catheter removal decisions were based on standardised clinical criteria rather than the surgical method. To conceal the transperineal puncture sites specific to H-FIRE, a sterile perineal dressing was applied to all patients postoperatively and removed after three days.

The operating surgeon was excluded from all postoperative assessments and data analysis. All outcome assessors, research nurses, and statisticians remained blinded to treatment allocation until the database was locked.

### Procedures

All surgeries were performed by a single urologist (HW) with extensive experience (>400 TURP and >200 H-FIRE procedures) under general anaesthesia with muscle paralysis. Patients were placed in the lithotomy position.

For participants presenting with concomitant bladder calculi, cystoscopic holmium laser lithotripsy was performed immediately prior to the assigned prostatic intervention within the same anaesthetic session. H-FIRE was performed using the REMEDINE composite steep pulse therapeutic apparatus. Under transrectal ultrasound guidance, 4 to 6 electrode needles were inserted transperineally via a template grid to target the median and lateral lobes. The spacing between needles (0.5–2.0 cm) and pulse parameters were adjusted based on prostate configuration.

TURP was performed using a standard monopolar tungsten loop electrode (160 W cutting/80 W coagulation). Resection proceeded systematically from the bladder neck to the verumontanum, removing tissue from the transition zone down to the surgical capsule.

All patients received prophylactic antibiotics (cephalosporins or quinolones) for 7 days postoperatively. α1-Adrenergic receptor antagonists were used to manage bladder spasms and were discontinued after catheter removal.

Assessments were conducted at baseline, during hospitalisation, and at 1 and 3 months postoperatively. PROs included the IPSS,^15^ 5-item version of the International Index of Erectile Function (IIEF-5),^16^ International Consultation on Incontinence Questionnaire Male Sexual Matters Associated with Lower Urinary Tract Symptoms Module (ICIQ-MLUTSsex),^17^ International Consultation on Incontinence Questionnaire (ICIQ),^18^ separate Expanded Prostate Cancer Index Composite (EPIC)^19^ pad-use item, Hospital Anxiety and Depression Scale (HADS),^20^ early postoperative urinary symptoms, and pain.^21^ Patient-reported outcome questionnaires were self-administered. Urodynamic assessments (Q_max_) were performed via standardised uroflowmetry when patients experienced a strong desire to void; instances of acute urinary retention preventing voluntary voiding were treated as missing Q_max_ values. Adverse events were monitored throughout the trial and for up to 2 years after treatment.

A comprehensive quality assurance program was established to minimise performance bias. For H-FIRE, transrectal ultrasound images were archived at three standardised timepoints (before insertion, needle placement, and after ablation) to verify targeting accuracy. For TURP, a blinded independent review process was used. All procedures were video-recorded. To ensure unbiased assessment, an independent statistician randomly selected three full-length videos from every block of 15 consecutive surgeries. These anonymised videos were reviewed by three independent expert urologists using a standardised technical proficiency questionnaire (Supplementary Appendix).

### Outcomes

The co-primary outcomes were the change from baseline in Q_max_ and in IPSS^15^ score at 3 months. Q_max_ is a key objective measure of urinary flow widely used in LUTS/BPO trials. The IPSS is an 8-item patient-reported tool scored from 0 to 35, with higher scores indicating more severe symptoms.

Secondary outcomes included changes from baseline in erectile function (IIEF-5 and ICIQ-MLUTSsex), postvoid residual volume, voided volume, urinary incontinence (ICIQ and EPIC pad-use), quality of life (IPSS-QoL and HADS), perioperative parameters, early postoperative symptoms, pain, and adverse events. Specifically, the occurrence of retrograde ejaculation was systematically evaluated through targeted clinical interviews and cross-verified using Item 3 of the ICIQ-MLUTSsex questionnaire (assessing the presence and volume of antegrade semen emission).

### Statistical analysis

The sample size was calculated to provide 90% power to simultaneously detect non-inferiority for the co-primary endpoints: change in Qmax and change in IPSS from baseline to 3 months. The non-inferiority margins were prespecified based on minimally clinically important differences established in prior literature: −4 mL/s for Qmax and 3 points for IPSS. Sample sizes were calculated separately for each endpoint using a one-sided significance level of 0.025, assuming a true between-group difference of 0. Standard deviations for the change from baseline were estimated at 6.0 mL/s for Qmax and 4.5 points for IPSS. Based on these parameters, 49 participants per group were required to satisfy the power requirements for both endpoint. To accommodate a 1:1 allocation ratio and an anticipated 20% attrition rate, the final enrolment target was set at 118 participants (59 per arm).

Efficacy analyses for primary outcomes were conducted on the full analysis set (FAS) and the per-protocol set (PPS). The FAS included all randomly assigned participants analysed according to their treatment allocation. The PPS was a subset of the FAS excluding participants with major protocol deviations as prespecified in statistical analysis plan, including missing baseline characteristics or missing primary outcome data. All randomised participants received their allocated intervention; therefore, exclusions from the PPS were not due to non-adherence to the assigned surgical procedure. Between-group differences in the change from baseline to 3 months were estimated using linear mixed-effect models. The models included the treatment group, baseline value, and stratification factors (age group [<70 versus ≥70 years] and prostate volume group [<60 versus ≥60 mL]) as fixed effects. Non-inferiority was established if the lower bound of the two-sided 95% CI for the between-group difference in the mean change in Qmax (H-FIRE minus TURP) was greater than −4 mL/s, and the upper bound for the between-group difference in the mean change in IPSS was less than 3 points. Missing data for primary endpoints in FAS were handled using model-based multiple imputation by chained equations under the missing-at-random assumption. The FAS analysis with multiple imputation was considered the primary analysis, and the PPS analysis without imputation was used to assess the robustness of the non-inferiority conclusion in the prespecified protocol-adherent population. The imputed variables were Qmax and IPSS measurements required to calculate change from baseline to 3 months. The imputation model included baseline, 1-month, and 3-month values of Qmax and IPSS, together with the stratification factors of age group (<70 versus ≥70 years) and prostate volume group (<60 versus ≥60 mL). Imputation was performed separately within each randomised treatment group. Two sensitivity analyses were performed to assess the robustness of the primary results: (1) a post-hoc adjustment for any baseline characteristics showing statistically significant imbalance; and (2) a two-way tipping point analysis under a missing-not-at-random assumption, varying shift parameters to identify the threshold at which non-inferiority conclusions would be overturned. For the tipping point analysis, shift values were added to the imputed 3-month Qmax and IPSS values separately in each treatment group. Each shifted dataset was analysed using the primary model, and the resulting estimates were pooled to determine whether the non-inferiority conclusion was preserved across plausible departures from the missing-at-random assumption. Prespecified subgroup analyses (stratified by age, body mass index, prostate volume, catheterisation status, bladder stone status, and prior medical treatment) were performed for both primary outcomes using regression models with interaction terms between the treatment group and the subgroup factor.

Secondary efficacy outcomes were analysed using a complete case analysis approach separately for each respective outcome, including all randomised participants with valid baseline and post-treatment data for that outcome. Therefore, the number of participants included in secondary analyses varied across outcomes according to data availability, and missing secondary outcome data were not imputed. Continuous variables assumed to follow an approximate normal distribution (e.g., voided volume) were analysed using LMMs. Variables with skewed distributions or ordinal data (e.g., Post-Void Residual Urine Volume, surgical pain scores) were analysed using the Wilcoxon rank sum test, with median differences and 95% CIs estimated using the Hodges-Lehmann method. Time-to-event outcomes (postoperative hospital stay and catheterisation duration) were analysed using Kaplan–Meier curves and compared via the log-rank test to evaluate recovery speed. Because secondary analyses were exploratory, no adjustments were made for multiplicity. Safety analyses included all participants who received the assigned intervention. Participants who were subsequently lost to follow-up remained in the safety analysis, and adverse events were summarised using all available safety information collected up to the last contact. Missing safety data were not imputed. Adverse events were summarised by frequency and severity; between-group comparisons were performed using the Chi-squared test or Fisher's exact test. All analyses were performed using SAS software, version 9.4 (SAS Institute Inc., Cary, NC). Detailed statistical methods are described in the statistical analysis plan, which is available in the supplementary appendix.

### Role of the funding source

The funders had no role in study design, data collection, data analyses, interpretation, or writing of the report.

## Results

**Fig. 1:**
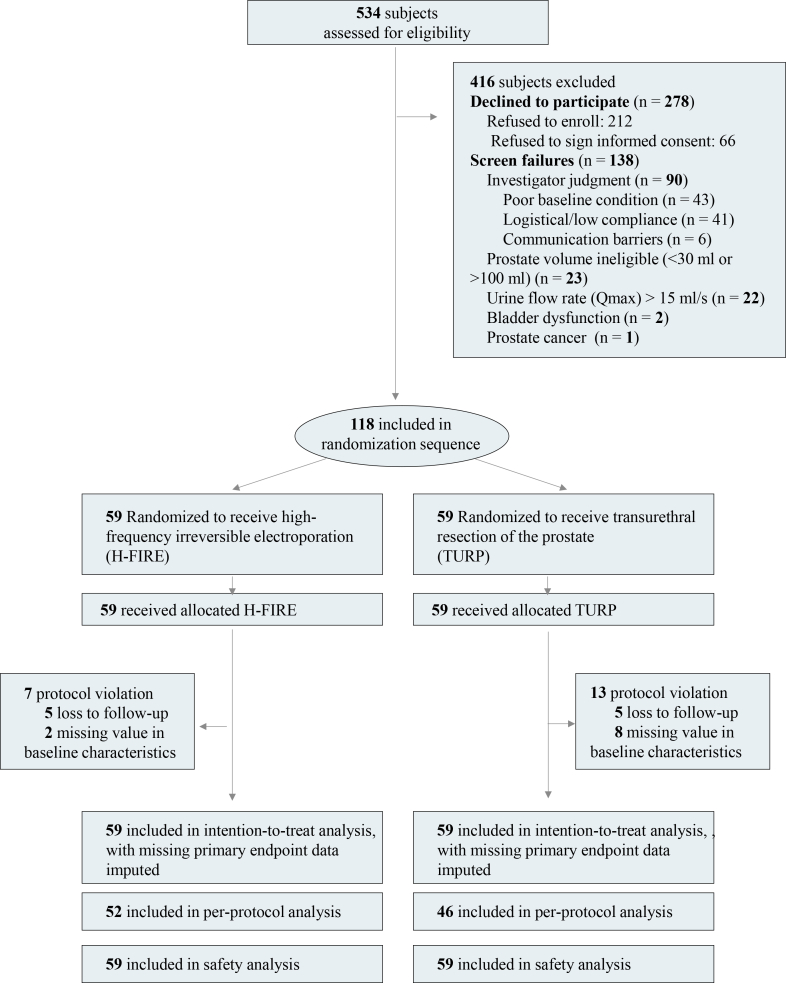
**Trial profile**.

From June 30, 2022, to May 25, 2025, a total of 118 participants were recruited and randomised (59 to H-FIRE, 59 to TURP; Fig. 1). Thus, all randomised participants received their allocated intervention and were included in the FAS and safety analysis. Participants with missing primary endpoint data were retained in the FAS through multiple imputation. Regarding the PPS, 7 participants in the H-FIRE group (5 lost to follow-up, 2 missing baseline data) and 13 participants in the TURP group (5 lost to follow-up, 8 missing baseline data) were excluded due to major protocol-defined deviations. Consequently, 52 patients in the H-FIRE group and 46 patients in the TURP group were analysed in the PPS. Independent blinded review of surgical videos confirmed that the TURP procedures were performed to a high technical standard (mean resection completeness score 4.8/5.0; Supplementary Table S7), ensuring a rigorous comparison.

At the 3-month primary endpoint visit, data completeness was high: 52 (88.1%) of 59 participants in the H-FIRE group and 46 (78.0%) in the TURP group had valid Qmax recordings and completed the IPSS questionnaire (Supplementary Table S5). Baseline demographic and clinical characteristics were generally well-balanced (Table 1), although minor imbalances in body mass index and serum sodium were noted (Supplementary Table S1). The cohort was characterised by a high prevalence of preoperative catheterisation (33.9% [20/59] versus 37.3% [22/59]) and relatively low baseline sexual function (median IIEF-5 score 2.0 [IQR 0.0–19.0] versus 3.0 [0.0–16.0]).

**Table 1:** Baseline clinical participant characteristics.

Characteristics	H-FIRE group (n = 59)	TURP group (n = 59)
Age, year, mean ± SD	69.58 ± 6.44	68.88 ± 5.58
<70	28 (47.46%)	29 (49.15%)
≥70	31 (52.54%)	30 (50.85%)
Race, n (%)		
Han	59 (100.00%)	59 (100.00%)
Others	0 (0)	0 (0)
Body mass index, kg/m^2^, mean ± SD	24.44 ± 2.21	23.14 ± 2.96
Comorbidity		
Hypertension	32 (54.24%)	24 (40.68%)
Diabetes Mellitus	14 (23.73%)	7 (11.86%)
Coronary heart disease	5 (8.47%)	0 (0.00%)
Gout	4 (6.78%)	1 (1.69%)
Medical treatment of BPH before surgery, n (%)	41 (69.49%)	38 (64.41%)
5α-reductase inhibitors	22 (37.29%)	20 (33.90%)
α1-adrenergic receptor antagonists	4 (6.78%)	4 (6.78%)
Combination of both drugs mentioned above	4 (6.78%)	4 (6.78%)
Others	19 (32.20%)	18 (30.51%)
Prostate-specific antigen, ng/mL, M (Q1, Q3)	6.99 (2.72, 12.90)	3.59 (2.16, 9.27)
Prostate volume, mL		
M (Q1, Q3)	58.44 (49.06, 75.51)	58.23 (43.66, 77.83)
<60	30 (50.85%)	30 (50.85%)
≥60	29 (49.15%)	29 (49.15%)
Digital rectal examination, n (%)		
Positive	3 (5.08%)	3 (5.08%)
Negative	56 (94.92%)	56 (94.92%)
Bladder calculi, n (%)		
Yes	8 (13.56%)	6 (10.17%)
No	51 (86.44%)	53 (89.83%)
Urinary catheterisation before surgery, n (%)		
Yes	20 (33.90%)	22 (37.29%)
No	39 (66.10%)	37 (62.71%)
Preoperative clinical characteristics		
Qmax, mL/s, mean ± SD	7.90 ± 3.08	7.28 ± 3.44
IPSS, points, mean ± SD	19.83 ± 5.78	19.64 ± 5.02
IIEF, points, M (Q1, Q3)	2.00 (0.00, 19.00)	3.00 (0.00, 16.00)
ICIQ- MLUTSsex, points, M (Q1, Q3)	5.00 (2.00, 6.00)	5.00 (2.00, 6.00)
PVRU, mL, M (Q1, Q3)	40.00 (5.00, 110.00)	39.00 (5.00, 170.00)
Voided Volume, mL, M (Q1, Q3)	116.94 (81.34, 193.14)	104.15 (66.60, 153.76)
ICIQ for urinary incontinence, points		
Free	51 (86.44%)	46 (77.97%)
≥1	8 (13.56%)	13 (22.03%)
EPIC pad-use, n (%)		
Free	58 (98.31%)	57 (96.61%)
≥1	1 (1.69%)	2 (3.39%)
HADS–depression, points, M (Q1, Q3)	1.00 (0.00, 3.00)	1.00 (0.00, 4.00)
HADS–anxiety, points, M (Q1, Q3)	0.00 (0.00, 2.00)	0.00 (0.00, 1.00)
Haemoglobin, g/L, mean ± SD	139.07 ± 14.75	141.07 ± 14.87
Serum sodium, mmol/L, Mean ± SD	138.98 ± 3.24	140.55 ± 2.55

M (Q1, Q3), median (the first quartile, the third quartile); SD, standard deviation; Qmax, maximum urinary flow rate; IPSS, International Prostate Symptom Score; IIEF, International Index of Erectile Function; ICIQ, International Consultation on Incontinence Questionnaire; ICIQ-MLUTSsex, Incontinence Questionnaire Male Sexual Matters Associated with Lower Urinary Tract Symptoms Module; PVRU, Post-Void Residual Urine Volume; EPIC, Expanded Prostate Cancer Index Composite; QoL, Quality of Life; HADS, Hospital Anxiety and Depression Scale.

At the 3-month follow-up, the majority of patients in both groups (64.4% [38/59] in H-FIRE versus 62.7% [37/59] in TURP) remained unsure of their treatment allocation. To formally quantify this, Bang's Blinding Index was calculated, yielding 0.051 for the H-FIRE group and 0.102 for the TURP group. Because both values are proximate to 0 (ideal blinding), it indicates that the unified counselling protocol largely neutralised the unblinding risk posed by differing catheterisation durations (Supplementary Table S6), confirming the efficacy of the sham perineal dressing (Supplementary Figure S1) and unified care protocol.

**Fig. 2:**
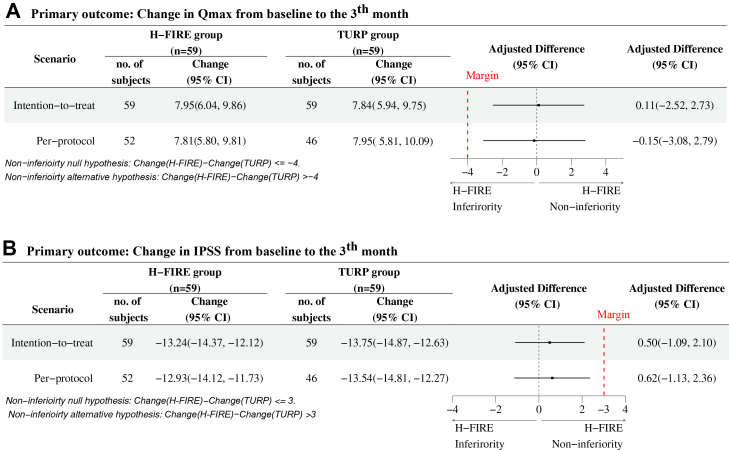
**Non-inferiority analysis of the primary outcomes at 3 months.** The forest plot displays the adjusted differences (H-FIRE minus TURP) in the change from baseline for Qmax **(A)** and Total IPSS **(B)**. The solid squares represent the point estimates of the difference, and the horizontal bars represent the 95% CIs. The vertical dashed lines indicate the prespecified non-inferiority margins (−4.0 mL/s for Qmax; +3.0 points for IPSS).

The primary analysis confirmed the non-inferiority of H-FIRE compared with TURP regarding both urodynamic improvement and symptom relief at 3 months (Fig. 2, Table 2). The co-primary endpoints were analysed in both the FAS and the PPS, as prespecified for this non-inferiority trial.

**Table 2:** Efficacy analyses on primary and secondary outcomes.

Outcomes	H-FIRE group (n = 59)		TURP group (n = 59)		Difference (95% CI)	p
	n	Estimation (95% CI)	n	Estimation (95% CI)		
Primary outcomes						
1. Change in Qmax, mL/s^a^						
Intention-to-treat analysis	59	7.95 (6.04, 9.86)	59	7.84 (5.94, 9.75)	0.11 (−2.52, 2.73)	0.001
Per-protocol analysis	52	7.81 (5.80, 9.81)	46	7.95 (5.81, 10.09)	−0.15 (−3.08, 2.79)	0.005
2. Change in IPSS, Points^a^						
Intention-to-treat analysis	59	−13.24 (−14.37, −12.12)	59	−13.75 (−14.87, −12.63)	0.50 (−1.09, 2.10)	0.001
Per-protocol analysis	52	−12.93 (−14.12, −11.73)	46	−13.54 (−14.81, −12.27)	0.62 (−1.13, 2.36)	0.004
Secondary outcomes						
1. Sexual function						
Change in IIEF at 3 months, points^a^	52	−2.79 (−4.43, −1.16)	53	−1.28 (−2.90, 0.33)	−1.51 (−3.81, 0.79)	0.195
Change in ICIQ-MLUTSsex at 3 months, points^a^	52	0.53 (0.05, 1.00)	51	0.77 (0.30, 1.25)	−0.25 (−0.92, 0.43)	0.469
2. Change in PVRU at 3 months, mL^b^	53	−15.00 (−55.00, 0.00)	53	−22.00 (−38.00, 0.00)	0.00 (−21.00, 21.00)	0.929
3. Change in voided volume at 3 months^a^	52	53.92 (27.94, 79.90)	46	59.90 (32.26, 87.55)	−5.98 (−44.13, 32.16)	0.756
4. Urinary incontinence at 3 months						
Change in ICIQ at 3 months, Points^a^	51	−0.36 (−1.00, 0.27)	52	−0.26 (−0.89, 0.37)	−0.10 (−1.00, 0.79)	0.817
EPIC pad-use at 3 months^b^	52	0.00 (0.00, 0.00)	52	0.00 (0.00, 0.00)	0.00 (0.00, 0.00)	0.451
EPIC pad-free, %^c^	52	94.23 (84.05, 98.79)	52	90.38 (78.97, 96.80)	3.85 (−6.37, 14.06)	0.715
5. Change in quality of life at 3 months						
IPSS QoL, points^b^	54	−2.00 (−3.00, −2.00)	53	−2.00 (−3.00, −2.00)	0.00 (−1.00, 0.00)	0.431
HADS–depression, points^a^	52	−1.15 (−1.53, −0.76)	52	−0.96 (−1.35, −0.58)	−0.19 (−0.73, 0.36)	0.503
HADS–anxiety, points^a^	52	−0.68 (−1.00, −0.35)	52	−0.52 (−0.85, −0.20)	−0.15 (−0.61, 0.30)	0.512
6. Surgical pain assessment at 3 months						
Pain at rest, points^b^	52	0.00 (0.00, 0.00)	52	0.00 (0.00, 0.00)	0.00 (0.00, 0.00)	0.102
Pain-free, %^c^	52	98.08 (89.74, 99.95)	52	90.38 (78.97, 96.80)	7.69 (−1.15, 16.53)	0.205
Pain during normal activities, points^b^	52	0.00 (0.00, 0.00)	52	0.00 (0.00, 0.00)	0.00 (0.00, 0.00)	0.981
Pain-free, %^c^	52	94.23 (84.05, 98.79)	52	94.23 (84.05, 98.79)	0.00 (−8.96, 8.96)	>0.999
Pain during sex, exercising, or strenuous work, points^b^	47	0.00 (0.00, 0.00)	50	0.00 (0.00, 0.00)	0.00 (0.00, 0.00)	0.168
Pain-free, %^c^	47	100.00 (92.45, 100.00)	50	96.00 (86.29, 99.51)	4.00 (−1.43, 9.43)	0.495
Worst pain, points^b^	52	0.00 (0.00, 0.00)	52	0.00 (0.00, 0.00)	0.00 (0.00, 0.00)	0.301
Pain-free, %^c^	52	94.23 (84.05, 98.79)	52	98.08 (89.74, 99.95)	−3.85 (−11.20, 3.51)	0.618
7. Perioperative outcomes						
Operative time, minute^b^	59	25.00 (22.00, 28.00)	59	45.00 (39.00, 60.00)	−21.00 (−30.00, −15.00)	<0.0001
Postoperative hospital stay, day^b^	59	2.00 (2.00, 2.00)	59	4.00 (3.00, 4.00)	−2.00 (−2.00, −1.00)	<0.0001
Catheterisation duration, day^b^	58	19.00 (15.00, 22.00)	59	4.00 (4.00, 5.00)	15.00 (11.00, 18.00)	<0.0001
Change in haemoglobin, g/L						
At 6th h^a^	56	−1.56 (−3.81, 0.70)	59	−4.96 (−7.16, −2.76)	3.40 (0.25, 6.56)	0.035
At 24th h^a^	56	0.82 (−1.84, 3.48)	59	−12.14 (−14.72, −9.55)	12.96 (9.24, 16.67)	<0.0001
Change in serum sodium, mmol/L						
At 6th h^a^	57	−0.49 (−1.26, 0.28)	59	−1.17 (−1.93, −0.42)	0.69 (−0.41, 1.78)	0.217
At 24th h^a^	56	−1.93 (−2.75, −1.12)	59	−2.35 (−3.14, −1.56)	0.42 (−0.74, 1.57)	0.477
8. EPUS at 3 months, points^b^	52	1.00 (1.00, 1.00)	52	1.00 (0.00, 1.00)	0.00 (0.00, 1.00)	0.089

Qmax, maximum urinary flow rate; IPSS, International Prostate Symptom Score; IIEF, International Index of Erectile Function; ICIQ, International Consultation on Incontinence Questionnaire; ICIQ-MLUTSsex, Incontinence Questionnaire Male Sexual Matters Associated with Lower Urinary Tract Symptoms Module; PVRU, Post-Void Residual Urine Volume; EPIC, Expanded Prostate Cancer Index Composite; QoL, Quality of Life; HADS, Hospital Anxiety and Depression Scale; EPUS, Early Postoperative Urinary Symptoms.

Primary outcomes were analysed in both the Full Analysis Set (FAS) and Per-Protocol Set (PPS). The FAS included all randomised participants according to randomised treatment allocation, with missing primary endpoint data handled using multiple imputation by chained equations under the missing-at-random assumption. The PPS excluded participants with major protocol-defined deviations, including missing baseline characteristics or missing primary outcome data. For Qmax (larger is better), the non-inferiority margin was −4 mL/s. For IPSS (smaller is better), the non-inferiority margin was 3 points. Secondary outcomes were analysed using complete case analysis for each respective outcome, including randomised participants with valid baseline and post-treatment data for that outcome. Missing secondary outcome data were not imputed; therefore, denominators varied across secondary outcomes.

a Continuous normally distributed variables are presented as Mean (95% Confidence Interval). The difference between groups was estimated using linear mixed models with the change from baseline as the dependent variable. The model was adjusted for the baseline value and stratification factors: age group (<70 years versus ≥70 years) and prostate volume group (<60 mL versus ≥60 mL).

b Skewed, ordinal, or count variables are presented as Median (95% Confidence Interval). Due to skewed distribution or ordinal nature, between-group comparisons were performed using the Wilcoxon Rank Sum test without covariate adjustment. Values represent the Median difference (Hodges-Lehmann estimate) and 95% Confidence Interval.

c The proportion of “Free” patients (score = 0) was estimated with 95% Confidence Interval. The difference in proportion and 95% Confidence Interval was assessed using Clopper-Pearson exact. p values was obtained from Fisher's exact test.

In the FAS, the mean improvement in Q_max_ was 7.95 mL/s (95% CI 6.04–9.86) in the H-FIRE group and 7.84 mL/s (95% CI 5.94–9.75) in the TURP group. The adjusted between-group difference was 0.11 mL/s (95% CI −2.52 to 2.73; p = 0.001 for non-inferiority). Since the lower limit of the 95% CI (−2.52 mL/s) was significantly above the prespecified non-inferiority margin of −4.0 mL/s, H-FIRE met the non-inferiority criterion for Q_max_. Consistent results were observed in the PPS. Longitudinal analysis showed that these improvements were sustained throughout the follow-up period (Supplementary Figure S8B), with consistent trends observed in subgroups of patients with baseline catheterisation (Supplementary Figure S9B) and bladder calculi (Supplementary Figure S10B).

Regarding urinary symptoms, both groups achieved substantial reductions in total IPSS. The mean change was −13.24 points (95% CI −14.37 to −12.12) for H-FIRE and −13.75 points (95% CI −14.87 to −12.63) for TURP. The adjusted difference was 0.50 points (95% CI −1.09 to 2.10; p = 0.001 for non-inferiority). With the upper limit of the 95% CI (2.10 points) falling below the margin of 3.0 points, the non-inferiority of H-FIRE for symptom relief was established. Consistent results were also observed in the PPS population. Analysis of IPSS subdomains revealed comparable improvements in both voiding and storage symptoms between groups (Supplementary Table S3).

The robustness of the non-inferiority conclusion was further corroborated by tipping point analyses (Supplementary Figures S2 and S3) and sensitivity analyses adjusting for baseline imbalances in BMI and serum sodium (Supplementary Table S2). Furthermore, subgroup analyses demonstrated consistent treatment effects across age, prostate volume, and baseline characteristics (Supplementary Figures S4 and S5).

Secondary efficacy outcomes were interpreted as supportive and exploratory findings and were analysed using complete case analysis for each respective outcome, as prespecified in the analysis plan. Secondary functional outcomes regarding voiding and incontinence were comparable between groups. Improvements in PVRU (median difference 0.00 mL, 95% CI −21.00 to 21.00; p = 0.929) and voided volume (adjusted difference −5.98 mL, 95% CI −44.13 to 32.16; p = 0.756) were similar across arms. The EPIC pad-free rate at 3 months was high in both groups (94.2% [49/52] for H-FIRE versus 90.4% [47/52] for TURP; difference 3.8%, 95% CI −6.4 to 14.1; p = 0.715), with no significant difference in ICIQ scores. Quality of life improvements (IPSS-QoL) and psychological status (HADS) showed no significant between-group differences.

Regarding sexual function, although the overall IIEF-5 scores showed no significant difference in change from baseline (adjusted difference −1.51 points, 95% CI −3.81 to 0.79; p = 0.195; domain breakdown in Supplementary Table S4), a distinct profile emerged regarding ejaculation. Analysis of adverse events (Table 3) revealed that retrograde ejaculation occurred in 0% (0/59) participants in the H-FIRE group compared to 54.2% (32/59) in the TURP group (p < 0.0001). This suggests that while erectile recovery was similar, H-FIRE offered superior preservation of antegrade ejaculation.

**Table 3:** Safety analysis.

Event	Overall (n = 118)	H-Fire group (n = 59)	TURP group (n = 59)	p
Adverse event, n (%)^a^	112 (94.92%)	53 (89.83%)	59 (100.00%)	0.036
Number of AE				
1	18 (15.25%)	14 (23.73%)	4 (6.78%)	–
2	31 (26.27%)	11 (18.64%)	20 (33.90%)	–
>2	63 (53.39%)	28 (47.46%)	35 (59.32%)	–
Haematuria	93 (78.81%)	34 (57.63%)	59 (100.00%)	<0.0001
Grade I	74 (79.57%)	34 (100.00%)	40 (67.80%)	
Grade II	16 (17.20%)	0 (0.00%)	16 (27.12%)	
Grade III	3 (3.23%)	0 (0.00%)	3 (5.08%)	
Urinary infection	63 (53.39%)	35 (59.32%)	28 (47.46%)	0.196
Grade I	26 (41.27%)	16 (45.71%)	10 (35.71%)	
Grade II	37 (58.73%)	19 (54.29%)	18 (64.29%)	
Grade III	0 (0)	0 (0)	0 (0)	
Pain	56 (47.46%)	26 (44.07%)	30 (50.85%)	0.461
Grade I	54 (96.43%)	24 (92.31%)	30 (100.00%)	
Grade II	2 (3.57%)	2 (7.69%)	0 (0.00%)	
Grade III	0 (0)	0 (0)	0 (0)	
Urinary retention	43 (36.44%)	33 (55.93%)	10 (16.95%)	<0.0001
Grade I	40 (93.02%)	32 (96.97%)	8 (80.00%)	
Grade II	3 (6.98%)	1 (3.03%)	2 (20.00%)	
Grade III	0 (0)	0 (0)	0 (0)	
Retrograde ejaculation	32 (27.12%)	0 (0.00%)	32 (54.24%)	<0.0001
Grade I	32 (100.00%)	0 (0)	32 (100.00%)	
Grade II	0 (0)	0 (0)	0 (0)	
Grade III	0 (0)	0 (0)	0 (0)	
Urethral stricture	11 (9.32%)	5 (8.47%)	6 (10.17%)	0.752
Grade I	0 (0)	0 (0)	0 (0)	
Grade II	11 (100.00%)	5 (100.00%)	6 (100.00%)	
Grade III	0 (0)	0 (0)	0 (0)	
Fever	3 (2.54%)	1 (1.69%)	2 (3.39%)	>0.999
Grade I	2 (66.67%)	1 (100.00%)	1 (50.00%)	
Grade II	1 (33.33%)	0 (0.00%)	1 (50.00%)	
Grade III	0 (0)	0 (0)	0 (0)	
Serious adverse event, n (%)^a^	0 (0)	0 (0)	0 (0)	–

Grade I is mild (asymptomatic/observation), Grade II is moderate (minor intervention needed, limits daily activities), Grade III is severe (disabling, hospitalisation), Grade IV is life-threatening (urgent intervention), and Grade V is death.

a Neither grade IV nor grade V was reported in this trial.

H-FIRE demonstrated significant advantages in operative efficiency and haemodynamic stability (Table 2). The median operative time was significantly shorter for H-FIRE compared to TURP (25.0 min [IQR 22.0–28.0] vs 45.0 min [IQR 39.0–60.0]; median difference −21.0 min, 95% CI −30.0 to −15.0; p < 0.0001).

Notably, H-FIRE was associated with minimal blood loss. 24 h after surgery, the TURP group had a significant reduction in haemoglobin (mean change −12.14 g/L, 95% CI −14.72 to −9.55), whereas the H-FIRE group remained stable (0.82 g/L, −1.84 to 3.48), resulting in a significant between-group difference of 12.96 g/L (95% CI 9.24–16.6; p < 0.0001). Similarly, evaluation of electrolyte stability revealed only minimal, clinically insignificant reductions in serum sodium levels in both groups (−1.93 mmol/L for H-FIRE versus −2.35 mmol/L for TURP at 24 h), with no significant difference between the cohorts (adjusted difference 0.42 mmol/L, 95% CI −0.74 to 1.57; p = 0.48), reflecting the absence of fluid absorption complications.

Kaplan–Meier analysis of time to discharge confirmed a significantly shorter hospital stay for the H-FIRE group (median 2.0 days [IQR 2.0–2.0] vs 4.0 days [IQR 3.0–4.0]; p < 0.0001; Supplementary Figure S6).

However, catheterisation duration was significantly longer in the H-FIRE group (median 19.00 days, IQR 15.0–22.0]) compared with the TURP group (median 4.00 days, IQR 4.0–5.0; p < 0.0001), reflecting the expected postoperative oedema associated with non-thermal ablation. This difference in catheter removal trajectory is visualised in the Kaplan–Meier estimates (Supplementary Figure S7).

Overall, adverse events were reported in 89.83% (53/59) of the H-FIRE group and 100% (59/59) of the TURP group (p = 0.036; Table 3). The safety profiles differed distinctly: H-FIRE was associated with a significantly lower incidence of haematuria (57.63% [34/59] versus 100.00% [59/59]; p < 0.0001), consistent with the haemoglobin findings. Conversely, urinary retention requiring catheterisation/recatheterisation was more frequent in the H-FIRE group (55.93% [33/59]) compared with the TURP group (16.95% [10/59]; p < 0.0001). No cases of transurethral resection syndrome were reported in either group. There were no grade 3–4 adverse events related or unrelated to the study treatments. Furthermore, no deaths occurred during the trial.

## Discussion

In this randomised, double-blind trial utilising a sham protocol, high-frequency irreversible electroporation (H-FIRE) was non-inferior to the gold standard TURP regarding symptom relief (IPSS) and urodynamic improvement (Qmax) at three months. Beyond comparable efficacy, the two modalities demonstrated distinct clinical profiles.

The most compelling advantage of H-FIRE was the complete preservation of ejaculatory function (0% de novo retrograde ejaculation), a stark contrast to the 54% rate in the TURP arm. However, as recently highlighted by Valdivia y Alvarado et al., true sexual and fertility preservation encompasses not only the anatomical patency of antegrade ejaculation but also the integrity of semen parameters.^22^ While our trial confirms the preservation of the ejaculatory act, future objective assessments of semen quality are essential to fully validate the fertility-sparing potential of H-FIRE for younger patients. Furthermore, H-FIRE exhibited superior haemodynamic stability with negligible blood loss. This safety profile translated into tangible efficiency gains: H-FIRE patients had a significantly shorter hospital stay (median 2 days vs 4 days).

However, this benefit came with a specific trade-off: a prolonged duration of catheterisation (median 19 days vs 4 days). This extended requirement is inherent to the non-thermal ablative mechanism, attributable to two factors: (1) immediate reactive tissue oedema compressing the prostatic urethra, and (2) the gradual process of necrotic tissue resorption and sloughing, which can transiently obstruct the channel.

Collectively, these findings frame H-FIRE as a “function-sparing” strategy that facilitates genuine shared decision-making. Rather than universally replacing TURP, it offers patients a clear choice: exchange the risks of permanent sexual dysfunction and longer hospitalisation for a clinically demanding, albeit temporary, period of ambulatory catheter management. Because individual tolerance for prolonged catheterisation varies profoundly, the acceptability of this trade-off must be individualised, empowering patients to select the surgical modality that best aligns with their personal quality-of-life priorities.

A defining feature of the GIANT trial is its pursuit of rigorous internal validity through a randomised, double-blind design—a methodological benchmark rarely achieved in surgical device trials. Recognising that the “operator factor” and placebo effects often confound the assessment of subjective symptoms (IPSS) and sexual function, we implemented a multi-layered sham protocol. Physically, we minimised performance and detection bias by standardising postoperative care, including uniform bladder irrigation and the use of sham perineal dressings for all patients. Psychologically, we employed a unified risk counselling strategy to mitigate unblinding risks associated with differing side–effect profiles. The success of this blinding strategy was empirically confirmed: at the 3-month exit interview, the majority of participants remained unaware of their treatment allocation (Supplementary Table S6).

To verify that our results reflected the true efficacy of the technology rather than operator variability, we incorporated a dual-arm independent quality assurance programme. Blinded expert review of TURP procedures yielded consistently high technical scores (mean resection completeness score 4.8/5.0; see Supplementary Table S7). Similarly, procedural consistency in the H-FIRE arm was rigorously monitored via standardised multi-phase transrectal ultrasound imaging documentation. These empirical data confirm that H-FIRE was compared against optimal surgical performance, thereby precluding bias from substandard control techniques.

Furthermore, our analytic approach was designed to be robust. By employing a prespecified, multi-tiered strategy for Qmax that accounted for urinary retention events and conducting tipping point analyses to assess missing data (Supplementary Figures S2 and S3), we ensured that our non-inferiority conclusion was statistically conservative.

We acknowledge that the single-centre design and the reliance on a single expert surgeon limits immediate generalisability. Furthermore, our highly selected cohort comprised exclusively Han Chinese men with late-stage presentation (high preoperative catheter-dependence and severely impaired baseline erectile function), making it difficult to directly extrapolate these functional preservation findings to younger, sexually active Western populations. However, this was a deliberate methodological necessity rather than a limitation of resources. Implementing such a complex double-blind protocol—specifically the logistics of sham dressings, uniform irrigation, and unified counselling—across multiple institutions would have posed significant risks to protocol adherence and blinding integrity. Therefore, we prioritised internal validity over external generalisability for this pivotal trial.

Second, despite our meticulous sham protocol and unified risk counselling yielding highly favourable formal blinding indices (Bang's Blinding Index ≈ 0), we must acknowledge the inherent biological limitations of blinding in surgical trials. The discrepancy in postoperative catheterisation duration (median 19 versus 4 days) inevitably carries a residual risk of unblinding highly perceptive patients or caregivers. This unblinding risk warrants consideration, as it could theoretically introduce a degree of detection or reporting bias in patient-reported subjective endpoints (such as IPSS, QoL, and pain scores).

Third, regarding the follow-up duration, our chosen primary endpoint remains consistent with the latest EAU guidelines.^4^ Given the median catheterisation duration of 19 days in the H-FIRE arm, our 3-month (12-week) endpoint ensures that all patients have completed this critical recovery window. However, we strongly emphasise that a 3-month horizon is insufficient to establish true long-term therapeutic equivalence to TURP. Major determinants of clinical value in BPH surgery—such as long-term durability, the incidence of delayed bladder neck contracture, urethral stricture, and ultimately, retreatment rates—cannot be captured in this timeframe. This is particularly relevant for H-FIRE, a novel modality characterised by delayed tissue ablation and resorption. Therefore, our findings are strictly limited to short-term functional non-inferiority. Comprehensive long-term durability and late complication profiles are actively being tracked in our ongoing extension phase.

Our study represents a decisive step forward in the evidence hierarchy for H-FIRE. While a recent trial^23^ demonstrated the superiority of H-FIRE over pharmacological therapy (tamsulosin), GIANT is the first to rigorously challenge the surgical gold standard, TURP, in a head-to-head comparison.

Methodologically, GIANT distinguishes itself from previous pivotal MIST trials. For instance, the landmark study comparing prostatic artery embolisation with TURP was limited by an open-label design and insufficient power.^24^ In contrast, GIANT employed a strict double-blind, sham protocol and achieved adequate statistical power to robustly confirm non-inferiority.

In terms of clinical impact, our findings parallel the landmark UNBLOCS trial,^25^ which established non-inferiority for Thulium laser resection. However, a critical distinction remains: UNBLOCS compared two thermal modalities, both carrying risks of thermal injury to the neurovascular bundles. GIANT, using a non-thermal mechanism, demonstrated a unique functional advantage—0% retrograde ejaculation—which was not observed in thermal laser trials. This suggests that while UNBLOCS confirmed an alternative for resection, GIANT establishes a new paradigm for functional preservation (the “BPH Trifecta”: effective de-obstruction, safety, and complete ejaculatory sparing).

Clinically, the choice offered by H-FIRE is particularly relevant for sexually active men who prioritise functional integrity over immediate voiding recovery. From a healthcare resource perspective, the implications are substantial. While H-FIRE necessitates general anaesthesia, its superior haemodynamic profile positions it as an ideal candidate for ambulatory (day-case) surgery. Although participants in this trial remained hospitalised for a median of 2 days for protocol-mandated observation, the absence of irrigation requirements suggests a strong theoretical potential for H-FIRE to be integrated into an ambulatory (same-day discharge) care pathway. However, dedicated prospective studies using specific day-case protocols are required to clinically validate this hypothesis.

Looking forward, three key areas warrant further investigation. First, the long-term durability of H-FIRE requires confirmation. While 3-month outcomes demonstrate non-inferiority, data from our ongoing extension phase are needed to assess late-term recurrence rates and the incidence of urethral strictures compared with TURP. Second, the prolonged catheterisation time (median 19 days) remains the primary limitation of this non-thermal modality. Future research should focus on optimising ablation parameters or exploring pharmacological adjuncts to accelerate tissue resorption. Finally, formal health economic analyses are warranted to determine whether the cost savings achieved through reduced hospitalisation (day-case model) and zero transfusion requirements offset the costs of the H-FIRE technology and disposables.

In men with symptomatic BPH, H-FIRE is non-inferior to the gold standard TURP in providing short-term functional de-obstruction and symptom relief. The obvious clinical advantages of H-FIRE lie in its superior safety and “function-sparing” profile: it provides absolute preservation of antegrade ejaculation, negligible blood loss, and significantly shorter operative and hospitalisation times. While inherently associated with a prolonged period of temporary postoperative catheterisation due to delayed tissue resorption, H-FIRE represents a highly effective, tissue-selective alternative for patients prioritising the lifelong preservation of sexual function and possesses significant potential for ambulatory (day-case) implementation.

## Contributors

Concept and design: BM He and HF Wang. Acquisition, analysis, or interpretation of data: BM He, RB Li, DY Li, LQ Huang, ZK Shi, SD Wang, JL Wen, and JS Lu. Draughting of the manuscript: BM He. Critical revision of the manuscript for important intellectual content: BM He and HF Wang. Statistical analysis: BM He and ZC Jin. Administrative, technique, or material support: RB Li, ZK Shi, SD Wang, JL Wen, and JS Lu. Supervison: JL Wen, JS Lu, and HF Wang. Dr. Bi-Ming He and Prof. Hai-Feng Wang directly accessed and verified the underlying data in the study and take responsibility for the integrity of the data and the accuracy of the data analysis. Dr. Hai-Feng Wang was responsible for the final decision to submit the manuscript for publication. All authors read and approved the final version of the manuscript.

## Data sharing statement

De-identified participant data will be available upon reasonable request to the corresponding author for researchers who provide a methodologically sound proposal.

## Declaration of interests

All authors declare no competing financial interests. No support was received from any organisation for the submitted work, and there are no financial relationships with any organisations that might have an interest in the submitted work.

## Appendix A Supplementary data

Supplement

Protocol

Summary of changes in protocol

GIANT HFire vs TURP SAP V1.1
