# Supplementary material for: High-frequency irreversible electroporation versus transurethral resection of the prostate for benign prostatic hyperplasia (GIANT): a single-centre, randomised, double-blind, phase 3, non-inferiority trial
Source: eClinicalMedicine. 2026 Jul 2;97:104034. doi: 10.1016/j.eclinm.2026.104034 (PMC13352031; doi:10.1016/j.eclinm.2026.104034)
Supplement: Supplement [file mmc1.pdf]

Supplementary appendix

High-Frequency Irreversible Electroporation (H-FIRE) versus Transurethral Resection of the Prostate for Benign Prostatic Hyperplasia: A Randomised, Double-Blind, Phase 3, Non-Inferiority Trial (GIANT)

Bi-Ming He, MD, PhD-1#, Rong-Bing Li, MD-1, Dong-Yang Li, MD, PhD-1, Li-Qun Huang, MD, PhD-2, Zhi-Chao Jin, MD, PhD-3, Zhen-Kai Shi, MD-1, Shuai-Dong Wang, MD-1, Jia-Sun Lu, MD-1, Ji-Ling Wen, MD, PhD-4,1, and Hai-Feng Wang, MD, PhD-1#

Affiliations:

[1]Department of Urology, Shanghai East Hospital, School of Medicine, Tongji University, Shanghai, China

[2]Department of Urology, Shanghai Geriatric Medical Center, Zhongshan Hospital Fudan University Minhang Campus, Shanghai, China

[3]Department of Health Statistics, Naval Medical University, Shanghai, China

[4]Department of Urology, Jing'an District Central Hospital, Fudan University, Shanghai, China.

[#]Correspondence:

Prof. Hai-Feng Wang, MD, PhD, Department of Urology, Shanghai East Hospital, No. 150, Jimo Road, Shanghai, 200120, China. Email: kuohaiandrew2000@vip.sina.com.cn. Telephone: +8613681750891.

Dr. Bi-Ming He, MD, PhD, Department of Urology, Shanghai East Hospital, No. 150, Jimo Road, Shanghai, 200120, China. Email: 190589109@qq.com. Telephone: +8615502139410.

List of supplementary material

Section S1 Outcomes definition

Figure S1: Photographic documentation of the sham blinding protocol in the TURP group.

Figure S2: Tipping point analysis for the primary outcome: Change in Qmax.

Figure S3 Tipping point analysis for the primary outcome: Change in IPSS.

Figure S4 Subgroup analysis for the primary outcome of Qmax.

Figure S5 Subgroup analysis for the primary outcome of Total IPSS.

Figure S6 Kaplan-Meier estimates of postoperative hospital length of stay (time to discharge).

Figure S7: Kaplan-Meier estimates of time to catheter removal (de-catheterization).

Figure S8: Longitudinal changes in primary outcomes over the follow-up period.

Figure S9. Longitudinal changes in primary outcomes over the follow-up period in patients with catheterization at baseline.

Figure S10. Longitudinal changes in primary outcomes over the follow-up period in patients with bladder calculi at baseline.

Table S1. Baseline Clinical Participant Characteristics with Group Comparisons

Table S2 Sensitivity analysis for efficacy analysis on primary and secondary outcomes

Table S3: Breakdown of IPSS Scores by Voiding and Storage Subdomains at 3 months

Table S4 Detailed Analysis of Erectile Function (IIEF-5) at 3 month

Table S5 missing pattern in intention-to-treat set (n=118).

Table S6. Cross-tabulation of patients' perceived versus actual treatment allocation at 3 months post-surgery.

Table S7: Independent Assessment of Transurethral Resection of the Prostate (TURP) Quality

Outcomes definition

Outcome	Definition	Assessment Time
maximal flow rate (Q_{max})	Q_{max} is a widely used clinical measure of urodynamics evaluated by the urinary flow rate assessment for benign prostatic obstruction. Patients with bladder catheters will be assessed the Q_{max} after the removal of the catheter.<br><br>Q_{max} will be assessed via uroflowmetry. To standardize the assessment across all patients and visits, the following protocol will be strictly adhered to:<br>►Pre-assessment Preparation: Patients will be instructed to drink water prior to their visit and to delay voiding until they experience a persistent and strong desire to void.<br>►Measurement: The Q_{max} obtained during a single voluntary void will be recorded as the raw value for that visit.<br>►Handling of Acute Urinary Retention: If a participant is unable to void despite a persistent and strong desire to do so, and this is confirmed clinically, the event will be recorded as "acute urinary retention". For the purpose of Q_{max} analysis, this will be treated as a missing data point and assigned a value of NA in the dataset.	Baseline, 1month and 3 months

International Prostate Symptom Score (IPSS)	IPSS is a well-established and validated patient-reported outcome, with 7 items to assess the degree of urinary symptoms during the last 4 weeks, ranging from 0 to 35, with higher scores indicating more severe urinary symptoms. IPSS will be assessed for those who have removed the bladder catheter after the surgery. If patients still have bladder catheters, IPSS will not be assessed.	Baseline, 1month and 3 months
5-item version of the International Index of Erectile Function (IIEF-5)	IIEF-5 is a 5-item self-report questionnaire to the presence and severity of erectile dysfunction, ranging from 0 to 25, with higher scores indicating better erectile function. IIEF-5 will be assessed for those who have removed the bladder catheter after the surgery. If patients still have bladder catheters, IIEF-5 will not be assessed.	Baseline, 1month and 3 months
International Consultation on Incontinence Questionnaire Male Sexual Matters Associated with Lower Urinary Tract Symptoms Module (ICIQ-MLUTSsex)	ICIQ-MLUTSsex is a self-report questionnaire to assess sexual dysfunction in men with LUTS (Appendix 3). ICIQ-MLUTSsex will be assessed for those who have removed the bladder catheter after the surgery. If patients still with bladder catheters, ICIQ-MLUTSsex will not be assessed.	Baseline, 1month and 3 months

post-void residual urine volume (PVRU)	PVRU is the amount of urine retained in the bladder after a voluntary void and is a clinical measure for assessing benign prostatic obstruction.	Baseline, 1month and 3 months
Voided volume	Voided volume is the amount of urine that is a voluntary void and functions as a clinical measure for assessing LUTS.	Baseline, 1month and 3 months
ICIQ (International Consultation on Incontinence Questionnaire)	ICIQ is a 3-item self-report questionnaire for urinary incontinence, ranging from 0 to 21, with higher scores indicating worse incontinence. The ICIQ score will be assessed for those who removed the bladder catheter after the surgery. The ICIQ score will not be assessed if patients still have bladder catheters.	Baseline, 1month and 3 months
separate EPIC (Expanded Prostate Cancer Index Composite) pad-use item	EPIC pad-use item is a self-report questionnaire to record the daily pad use, with higher daily pad use indicating worse incontinence. EPIC pad-use score will be assessed for those who have removed the bladder catheter after the surgery. If patients still have bladder catheters, EPIC pad-use will not be assessed.	Baseline, 1month and 3 months
IPSS QoL subscore	IPSS QoL subscore is a 1-item self-report questionnaire to assess the quality of life with prostate symptoms, ranging from 0 to 6, with higher scores indicating a worse quality of life (Appendix	Baseline, 1month and 3 months

	1). IPSS QoL subscore will be assessed for those who have removed the bladder catheter after the surgery. The IPSS QoL subscore will not be assessed if patients still have bladder catheters.	
Hospital Anxiety and Depression Scale (HADS)	HADS is a self-assessment scale for evaluating the presence and severity of anxiety and depression, ranging from 0 to 42, with a higher score indicating more distress.	Baseline, 1month and 3 months
perioperative parameters, including operative time, the postoperative hospital stay, haemoglobin declination, serum sodium declination, and catheterisation duration.	Operative time is the time of the procedure of H-FIRE or TURP (If the patient has a combined bladder stone, the time for bladder lithotripsy will not be calculated).<br>Postoperative hospital stay is the length from the first day after surgery to discharge.<br>Haemoglobin declination is defined as the baseline haemoglobin minus postoperative haemoglobin.<br>Serum sodium declination is defined as the baseline serum sodium minus postoperative serum sodium.<br>Catheterisation duration is the length from the first day after surgery to catheterisation. The catheterisation day before surgery will not be calculated.	Baseline and during hospitalisation
Early postoperative urinary symptoms	The early postoperative urinary symptoms will be measured by a 5-item self-report questionnaire	Baseline, 1month and 3 months

include dysuria, urgency, or postmicturition pain.		
Pain	Pain will be assessed by 4-item surgical pain scale, ranging from 0 to 40, higher score indicating more severe pain.	Baseline, 1 week (phone),1month and 3 months

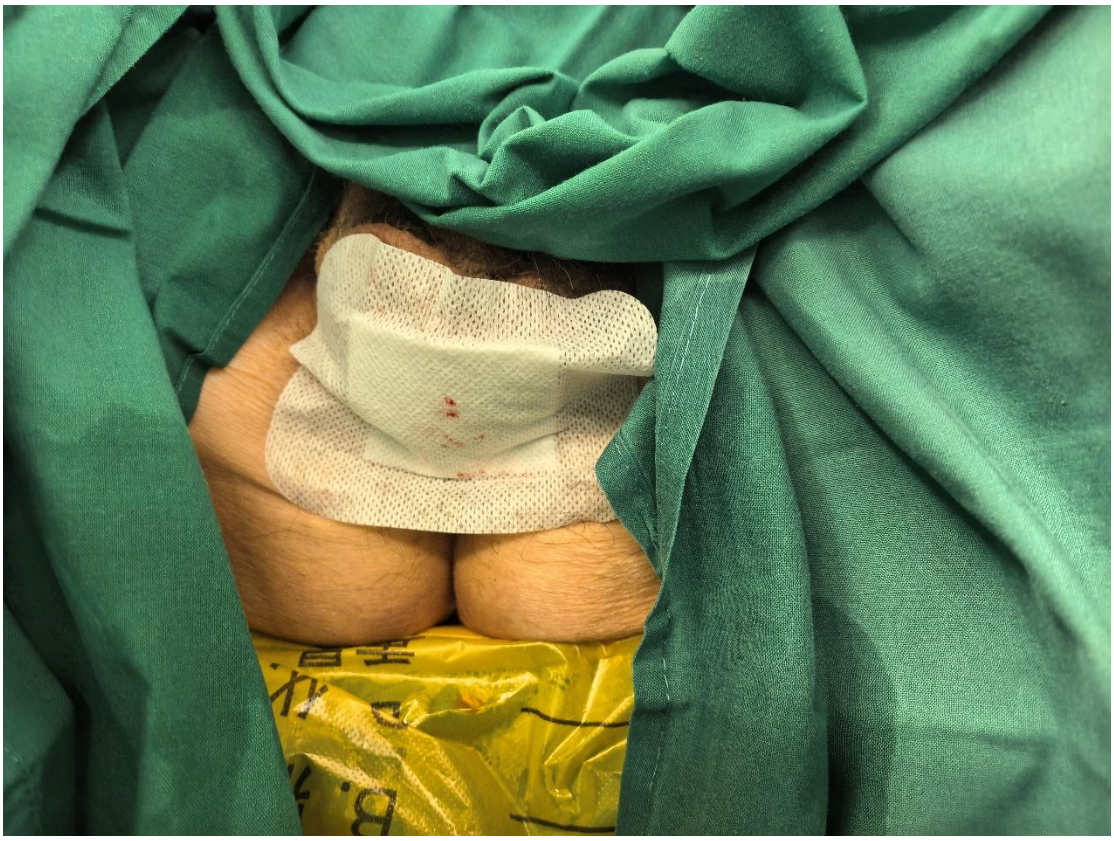

Figure S1. Photographic documentation of the sham blinding protocol in the TURP group.

This representative image demonstrates the physical masking strategy used to maintain the double-blind design. The participant underwent Transurethral Resection of the Prostate (TURP) and therefore had no transperineal needle insertions.

To prevent unblinding based on external physical cues:

(1) A sterile perineal dressing, identical to that used in the H-FIRE group, was applied immediately post-procedure.

(2) To simulate the visual appearance of postoperative oozing associated with transperineal needle puncture, a small amount of the patient's own blood (obtained directly from the indwelling catheter bag) was applied to the gauze.

(3) This dressing was kept in place for 3 days, consistent with the H-FIRE protocol.

This rigorous "visual placebo" ensured that patients could not deduce their treatment allocation by inspecting the perineal region.

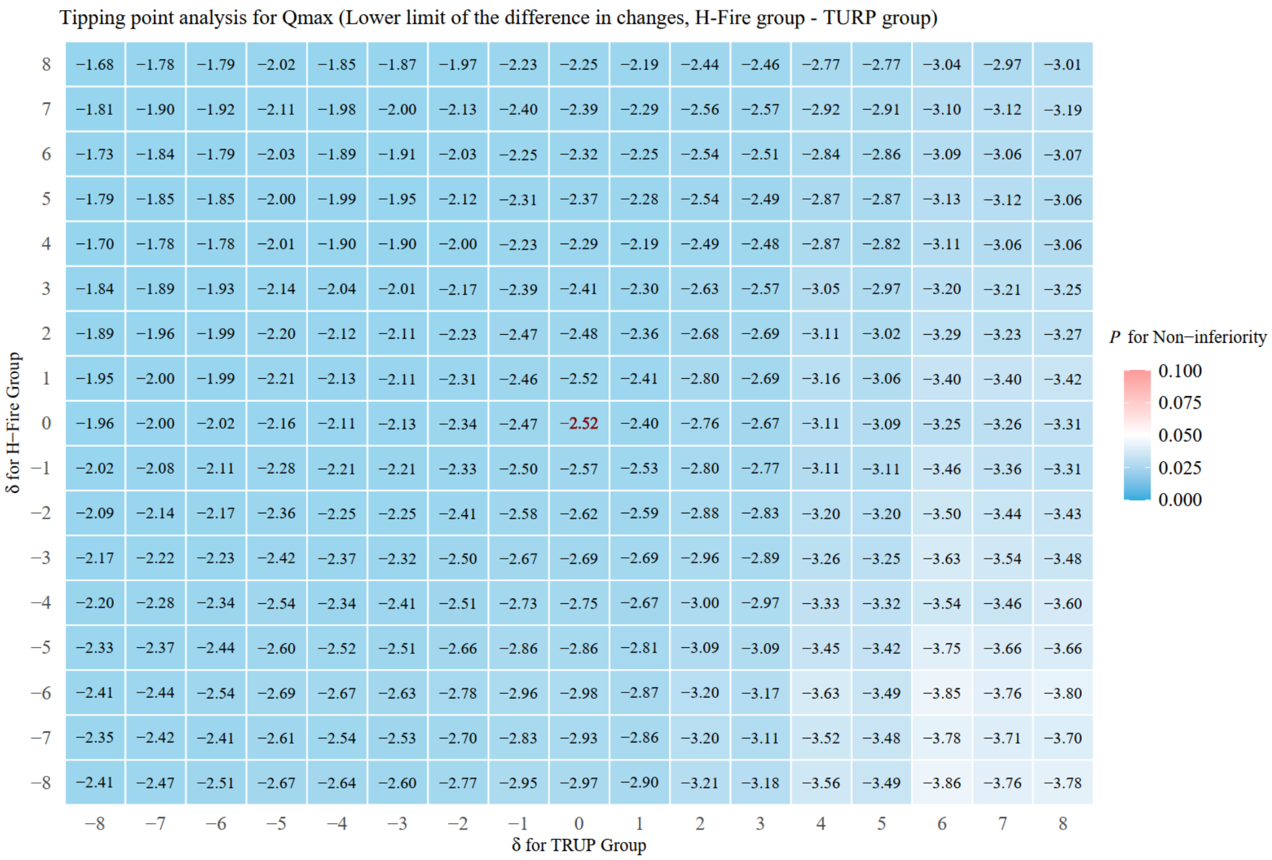

Figure S2. Tipping point analysis for the primary outcome: Change in Qmax.

The heatmap displays the P-values for non-inferiority testing of the change in Qmax from baseline at 3 months under different imputation scenarios for missing data. The x-axis (δ for TURP Group) and y-axis (δ for H-FIRE Group) represent the shift values added to the observed mean change for the imputed missing data in each respective group. The values within the cells represent the lower limit of the 95% confidence interval for the treatment difference (H-FIRE minus TURP). Blue regions indicate scenarios where non-inferiority is established (lower limit > -4.0 mL/s), while red/white regions would indicate failure to demonstrate non-inferiority. The analysis demonstrates that the non-inferiority conclusion holds true across all examined scenarios.

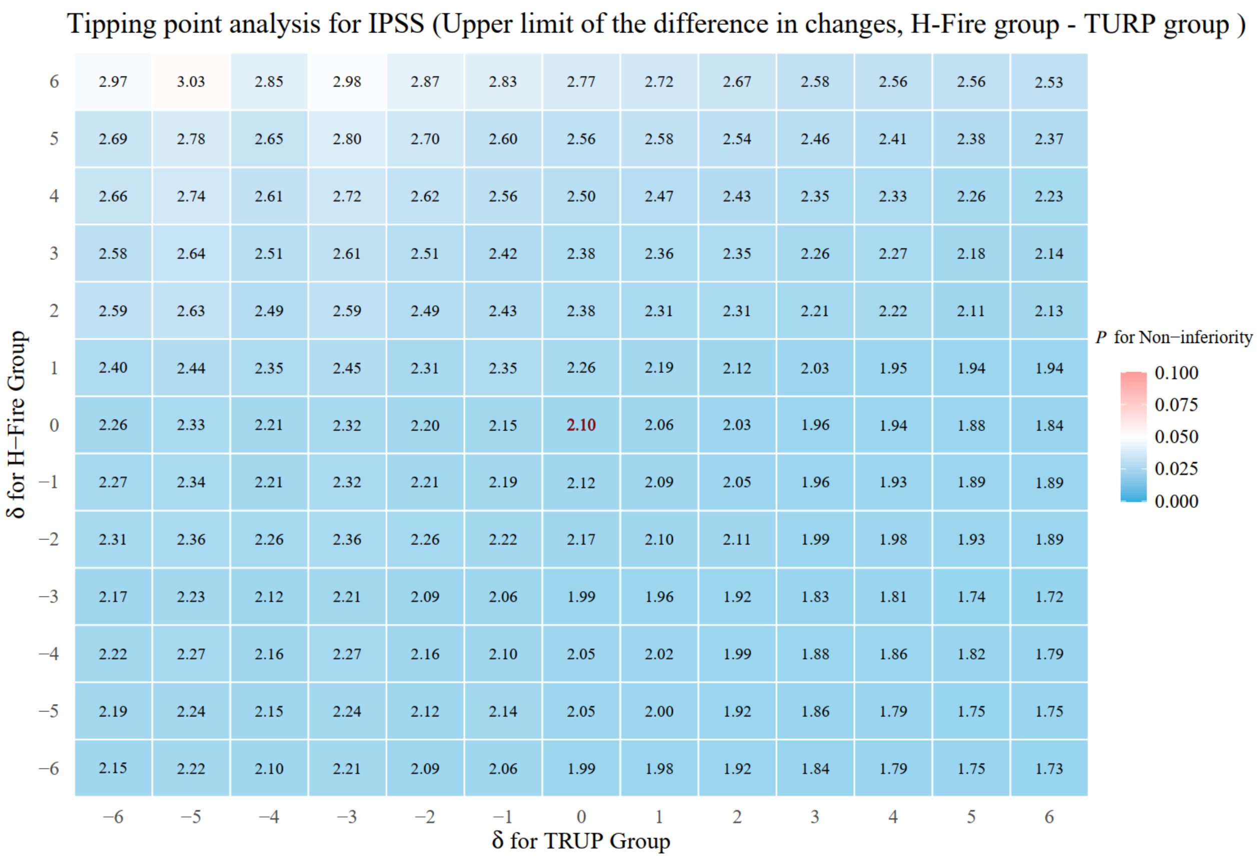

Figure S3. Tipping point analysis for the primary outcome: Change in IPSS.

The heatmap displays the P-values for non-inferiority testing of the change in Total IPSS from baseline at 3 months. The axes represent the shift values (δ) added to the observed mean change for missing data in the H-FIRE and TURP groups. The values within the cells represent the upper limit of the 95% confidence interval for the treatment difference (H-FIRE minus TURP). Blue regions indicate scenarios where non-inferiority is established (upper limit < 3.0 points). The consistent blue shading confirms that the study conclusion is robust to missing data assumptions.

(A) Subgroup analysis for Qmax in Intention-to-treat set

Subgroup	no.	H−Fire Group (N=58) no.	H−Fire Group (N=58) Change (95% CI)	TURP Group (N=58) no.	TURP Group (N=58) Change (95% CI)	Adjusted Difference (95% CI)	$P_{interaction}$
Overall	118	59	7.95(6.04, 9.86)	59	7.84(5.94, 9.75)	0.11(−2.52, 2.73)	
Age							0.128
<70	57	28	9.87(6.90, 12.84)	29	10.41(7.61, 13.21)	−0.54(−4.60, 3.51)	
>=70	61	31	5.80(3.28, 8.33)	30	5.43(2.84, 8.02)	0.37(−3.34, 4.08)	
Body mass index, kg/m2							0.771
<24	60	26	8.27(5.40, 11.14)	34	8.04(5.52, 10.55)	0.23(−3.58, 4.04)	
>=24	58	33	7.38(4.79, 9.97)	25	7.88(4.87, 10.89)	−0.50(−4.49, 3.50)	
Medical treatment of BPH							0.941
No	39	18	7.58(3.87, 11.28)	21	7.29(3.85, 10.74)	0.28(−4.68, 5.24)	
Yes	79	41	8.25(6.08, 10.41)	38	8.21(5.95, 10.47)	0.04(−3.05, 3.12)	
Prostate volume, ml							0.479
<60	60	30	6.62(4.16, 9.08)	30	5.59(3.10, 8.08)	1.03(−2.39, 4.46)	
>=60	58	29	9.53(6.73, 12.33)	29	9.84(6.88, 12.81)	−0.31(−4.34, 3.71)	
Bladder calculi							0.789
No	104	51	7.89(5.73, 10.04)	53	8.02(5.92, 10.12)	−0.13(−3.07, 2.80)	
Yes	14	8	8.45(5.09, 11.82)	6	6.57(2.47, 10.66)	1.89(−3.66, 7.44)	
Urinary catheterization							0.207
No	76	39	6.58(4.43, 8.74)	37	7.35(5.17, 9.54)	−0.77(−3.75, 2.21)	
Yes	42	20	9.06(4.75, 13.37)	22	8.76(5.22, 12.29)	0.30(−5.08, 5.69)	

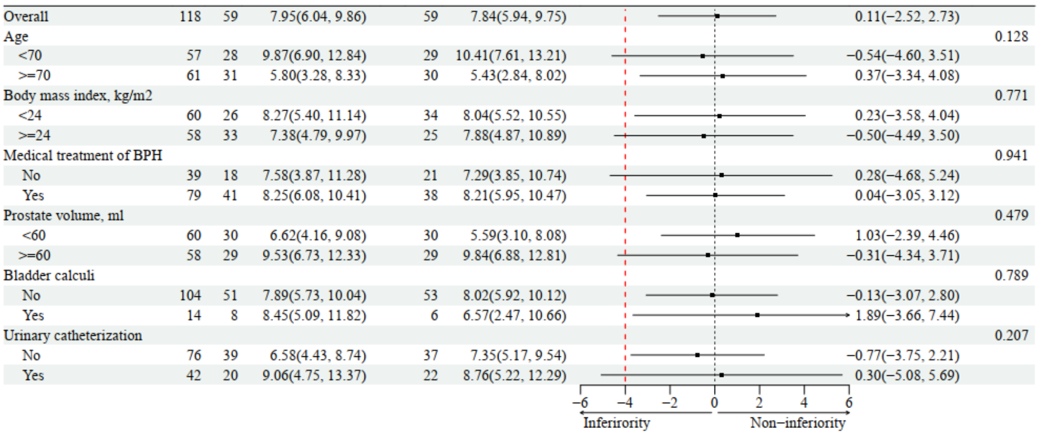

(B) Subgroup analysis for Qmax in Per-protocol set

Subgroup	no.	H−Fire Group (N=52) no.	H−Fire Group (N=52) Change (95% CI)	TURP Group (N=46) no.	TURP Group (N=46) Change (95% CI)	Adjusted Difference (95% CI)	$P_{interaction}$
Overall	98	52	7.81(5.80, 9.81)	46	7.95(5.81, 10.09)	−0.15(−3.08, 2.79)	
Age							0.091
<70	49	25	9.66(6.50, 12.82)	24	10.76(7.53, 13.99)	−1.10(−5.68, 3.47)	
>=70	49	27	5.71(2.91, 8.51)	22	5.31(2.18, 8.44)	0.40(−3.97, 4.76)	
Body mass index, kg/m2							0.955
<24	49	24	8.38(5.31, 11.45)	25	8.58(5.61, 11.55)	−0.20(−4.58, 4.18)	
>=24	49	28	6.87(3.89, 9.85)	21	7.60(4.18, 11.02)	−0.73(−5.33, 3.87)	
Medical treatment of BPH							0.956
No	33	16	7.74(3.53, 11.95)	17	7.69(3.66, 11.72)	0.05(−5.75, 5.86)	
Yes	65	36	7.96(5.60, 10.32)	29	8.23(5.59, 10.87)	−0.27(−3.82, 3.27)	
Prostate volume, ml							0.466
<60	49	27	6.49(3.86, 9.12)	22	5.87(2.95, 8.79)	0.62(−3.35, 4.59)	
>=60	49	25	9.38(6.22, 12.54)	24	9.65(6.42, 12.88)	−0.27(−4.80, 4.26)	
Bladder calculi							0.837
No	85	44	7.71(5.41, 10.01)	41	8.17(5.78, 10.57)	−0.46(−3.80, 2.87)	
Yes	13	8	8.58(4.80, 12.35)	5	7.86(2.78, 12.93)	0.72(−5.79, 7.22)	
Urinary catheterization							0.455
No	69	36	6.44(4.21, 8.66)	33	7.10(4.82, 9.37)	−0.66(−3.82, 2.50)	
Yes	29	16	10.07(4.52, 15.61)	13	10.35(4.81, 15.88)	−0.28(−8.01, 7.45)	

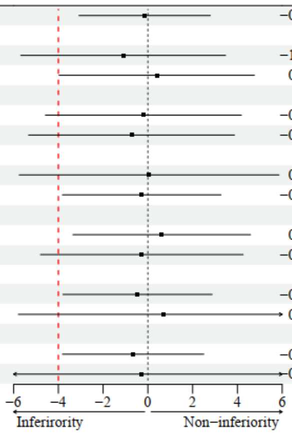

Figure S4 Subgroup analysis for the primary outcome of Qmax

The forest plots display the adjusted differences in the change in Qmax from baseline at 3 months between the H-FIRE and TURP groups across various prespecified subgroups. **(A)** Shows the analysis within the Intention-to-Treat (ITT) population. **(B)** Shows the analysis within the Per-Protocol (PP) population. The solid squares represent the point estimates of the adjusted difference, and the horizontal lines indicate the 95% confidence intervals. The vertical red dashed line represents the non-inferiority margin of -4.0 mL/s. P-values for interaction ($P_{interaction}$) are provided to assess the consistency of the treatment effect across subgroups. No significant interactions were observed, indicating consistent non-inferiority across all subgroups.

(A) Subgroup analysis for IPSS in Intention-to-treat set

Subgroup	no.	H−Fire Group (N=58) no.	H−Fire Group (N=58) Change (95% CI)	TURP Group (N=58) no.	TURP Group (N=58) Change (95% CI)	Adjusted Difference (95% CI)	$P_{interaction}$
Overall	118	59	−13.24(−14.37, −12.12)	59	−13.75(−14.87, −12.63)	0.50(−1.09, 2.10)	
Age							0.276
<70	57	28	−14.13(−15.47, −12.79)	29	−15.55(−16.82, −14.27)	1.42(−0.42, 3.25)	
>=70	61	31	−12.38(−14.15, −10.61)	30	−11.93(−13.78, −10.07)	−0.45(−3.03, 2.12)	
Body mass index, kg/m2							0.325
<24	60	26	−13.70(−15.46, −11.95)	34	−13.24(−14.78, −11.70)	−0.46(−2.86, 1.94)	
>=24	58	33	−12.90(−14.39, −11.41)	25	−14.42(−16.14, −12.69)	1.52(−0.74, 3.78)	
Medical treatment of BPH							0.441
No	39	18	−13.27(−15.57, −10.98)	21	−11.80(−13.90, −9.71)	−1.47(−4.56, 1.62)	
Yes	79	41	−13.15(−14.39, −11.92)	38	−14.80(−16.12, −13.49)	1.65(−0.17, 3.47)	
Prostate volume, ml							0.214
<60	60	30	−11.90(−13.61, −10.20)	30	−13.94(−15.64, −12.23)	2.03(−0.39, 4.46)	
>=60	58	29	−14.64(−16.02, −13.25)	29	−13.48(−14.88, −12.08)	−1.15(−3.11, 0.80)	
Bladder calculi							0.418
No	104	51	−13.38(−14.61, −12.14)	53	−14.02(−15.23, −12.82)	0.65(−1.08, 2.38)	
Yes	14	8	−11.68(−13.92, −9.43)	6	−11.22(−13.84, −8.59)	−0.46(−4.11, 3.19)	
Urinary catheterization							0.66
No	76	39	−12.71(−14.23, −11.19)	37	−13.44(−14.96, −11.91)	0.73(−1.40, 2.85)	
Yes	42	20	−14.34(−16.32, −12.37)	22	−13.99(−15.66, −12.32)	−0.36(−2.89, 2.18)	

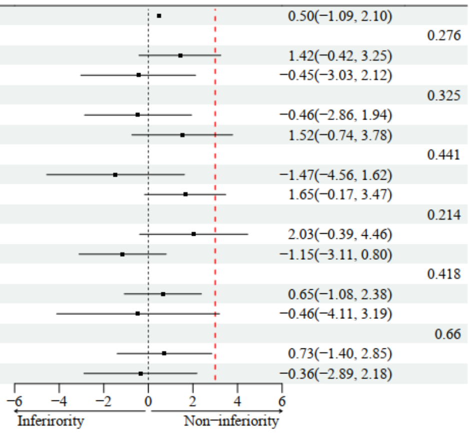

(B) Subgroup analysis for IPSS in Per-protocol set

Subgroup	no.	H−Fire Group (N=52) no.	H−Fire Group (N=52) Change (95% CI)	TURP Group (N=46) no.	TURP Group (N=46) Change (95% CI).	Adjusted Difference (95% CI)	$P_{interaction}$
Overall	98	52	−12.93(−14.12, −11.73)	46	−13.54(−14.81, −12.27)	0.62(−1.13, 2.36)	
Age							0.222
<70	57	25	−14.14(−15.49, −12.80)	24	−15.79(−17.16, −14.41)	1.64(−0.28, 3.57)	
>=70	61	27	−11.74(−13.70, −9.77)	22	−11.14(−13.32, −8.96)	−0.60(−3.54, 2.34)	
Body mass index, kg/m2							0.076
<24	60	24	−13.39(−15.31, −11.46)	25	−12.95(−14.79, −11.10)	−0.44(−3.20, 2.32)	
>=24	58	28	−12.40(−14.03, −10.77)	21	−14.31(−16.18, −12.44)	1.91(−0.60, 4.41)	
Medical treatment of BPH							0.198
No	39	16	−13.49(−16.05, −10.93)	17	−11.92(−14.37, −9.47)	−1.57(−5.10, 1.96)	
Yes	79	36	−12.62(−13.95, −11.28)	29	−14.47(−15.96, −12.98)	1.85(−0.15, 3.85)	
Prostate volume, ml							0.245
<60	60	27	−11.72(−13.57, −9.88)	22	−13.66(−15.71, −11.62)	1.94(−0.82, 4.70)	
>=60	58	25	−14.16(−15.63, −12.70)	24	−13.48(−14.98, −11.99)	−0.68(−2.77, 1.42)	
Bladder calculi							0.243
No	104	44	−13.12(−14.44, −11.81)	41	−13.82(−15.19, −12.45)	0.70(−1.20, 2.60)	
Yes	14	8	−11.01(−13.41, −8.61)	5	−9.76(−12.87, −6.65)	−1.25(−5.43, 2.93)	
Urinary catheterization							0.735
No	76	36	−12.55(−14.13, −10.96)	33	−13.31(−14.93, −11.68)	0.76(−1.49, 3.02)	
Yes	42	16	−13.48(−15.67, −11.30)	13	−13.39(−15.61, −11.17)	−0.09(−3.14, 2.96)	

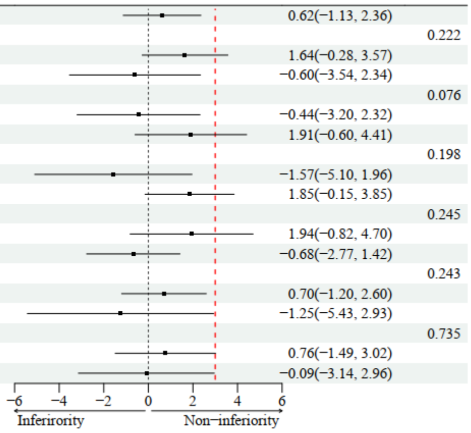

Figure S5 Subgroup analysis for the primary outcome of Total IPSS

The forest plots illustrate the adjusted differences in the change in Total IPSS from baseline at 3 months between the H-FIRE and TURP groups across prespecified subgroups. **(A)** Analysis in the Intention-to-Treat (ITT) population. **(B)** Analysis in the Per-Protocol (PP) population. The solid squares denote the point estimates of the adjusted difference, with horizontal lines representing the 95% confidence intervals. The vertical red dashed line marks the non-inferiority margin of +3.0 points. P-values for interaction ($P_{interaction}$) are shown on the right. Results demonstrate consistent non-inferiority of H-FIRE compared to TURP for symptom relief across all analyzed subgroups.

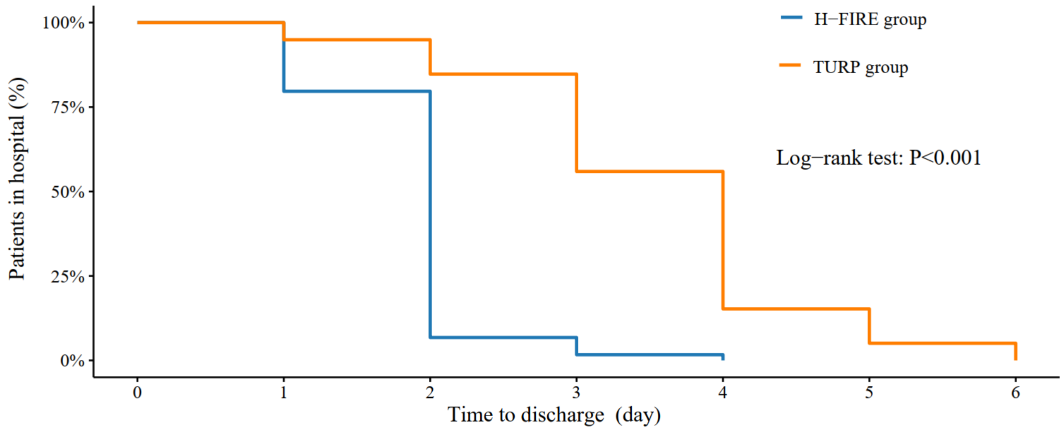

Number at risk

H−FIRE group	59	59	47	4	1	0	0
TURP group	59	59	56	50	33	9	3

Figure S6. Kaplan-Meier estimates of postoperative hospital length of stay (time to discharge). The figure displays the probability of remaining in the hospital post-surgery. In contrast to the catheterization data, the H-FIRE group (blue line) demonstrated a significantly faster discharge trajectory (median 2 days) compared to the TURP group (orange line, median 4 days). This advantage is attributable to the superior hemodynamic stability and lack of postoperative bladder irrigation requirements in the H-FIRE arm, facilitating earlier discharge despite the indwelling catheter. The difference was statistically significant (Log-rank test, $P < 0.001$).

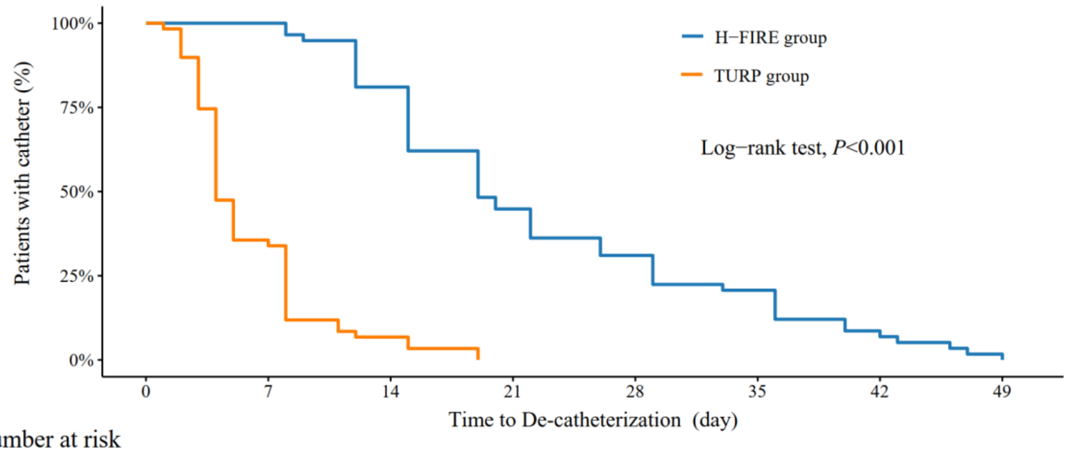

Number at risk

H−FIRE group	58	58	47	26	18	12	5	1
TURP group	59	21	4	0	0	0	0	0

Figure S7. Kaplan-Meier estimates of time to catheter removal (de-catheterization).

The figure displays the probability of retaining the urinary catheter over time. The orange line represents the TURP group, demonstrating the standard rapid removal of the catheter (median 4 days). The blue line represents the H-FIRE group, showing a significantly prolonged catheterization requirement (median 19 days). This extended duration in the H-FIRE arm reflects the specific mechanism of non-thermal ablation, which necessitates time for tissue resorption and edema resolution. The difference between groups was statistically significant (Log-rank test, $P < 0.001$).

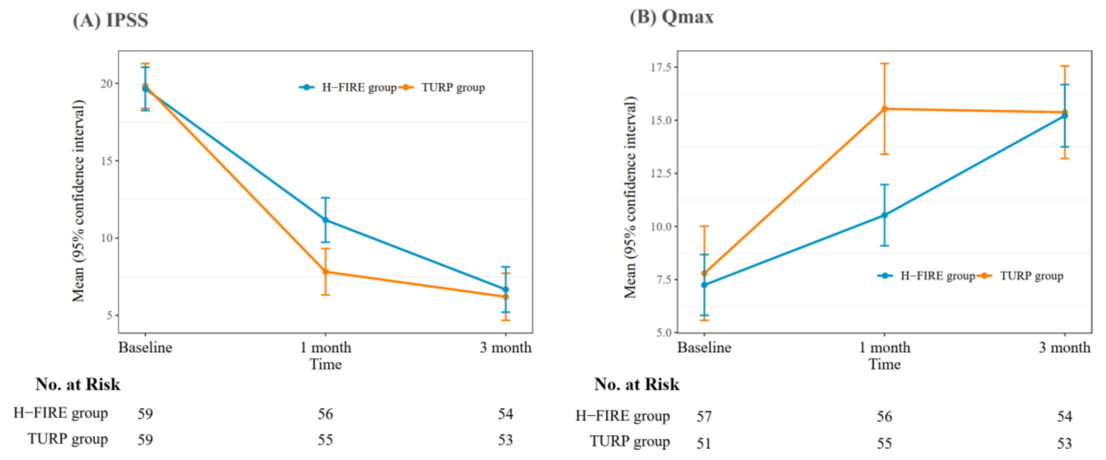

Figure S8. Longitudinal changes in primary outcomes over the follow-up period.

(A) Changes in the International Prostate Symptom Score (IPSS) from baseline to post-operative follow-up. **(B)** Changes in the maximum urinary flow rate (Qmax) from baseline to post-operative follow-up. The blue line represents the High-Frequency Irreversible Electroporation (H-FIRE) group, and the orange line represents the Transurethral Resection of the Prostate (TURP) group. Data points indicate mean values, and error bars represent 95% confidence intervals. The tables below the graphs show the number of participants evaluated at each time point.

Note that the number of evaluable patients for Qmax may increase at follow-up compared to baseline, as patients with acute urinary retention (AUR) at baseline were unable to provide a valid measurement (recorded as NA) until after surgical intervention.

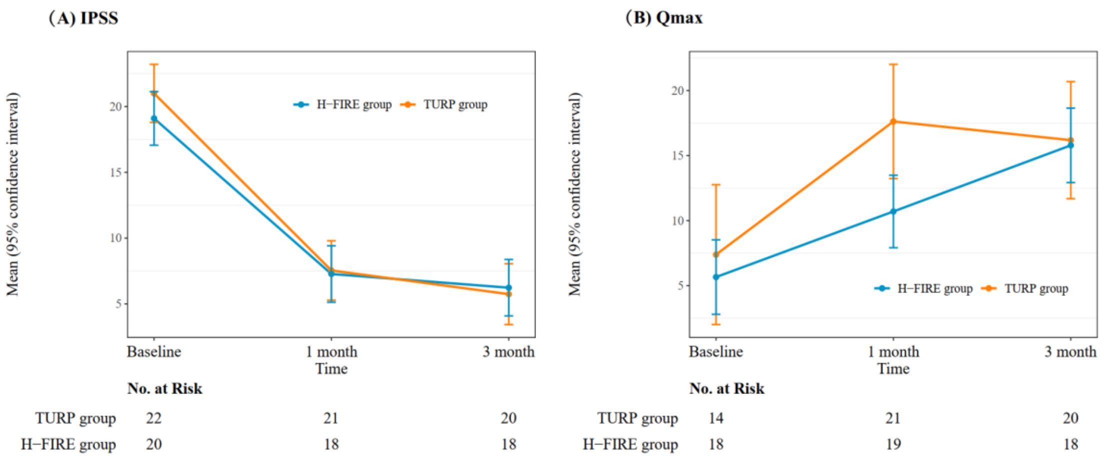

Figure S9. Longitudinal changes in primary outcomes over the follow-up period in patients with catheterization at baseline.

(A) Changes in the International Prostate Symptom Score (IPSS) from baseline to post-operative follow-up. **(B)** Changes in the maximum urinary flow rate (Qmax) from baseline to post-operative follow-up. The blue line represents the High-Frequency Irreversible Electroporation (H-FIRE) group, and the orange line represents the Transurethral Resection of the Prostate (TURP) group. Data points indicate mean values, and error bars represent 95% confidence intervals. The tables below the graphs show the number of participants evaluated at each time point.

Note that the number of evaluable patients for Qmax may increase at follow-up compared to baseline, as patients with acute urinary retention (AUR) at baseline were unable to provide a valid measurement (recorded as NA) until after surgical intervention.

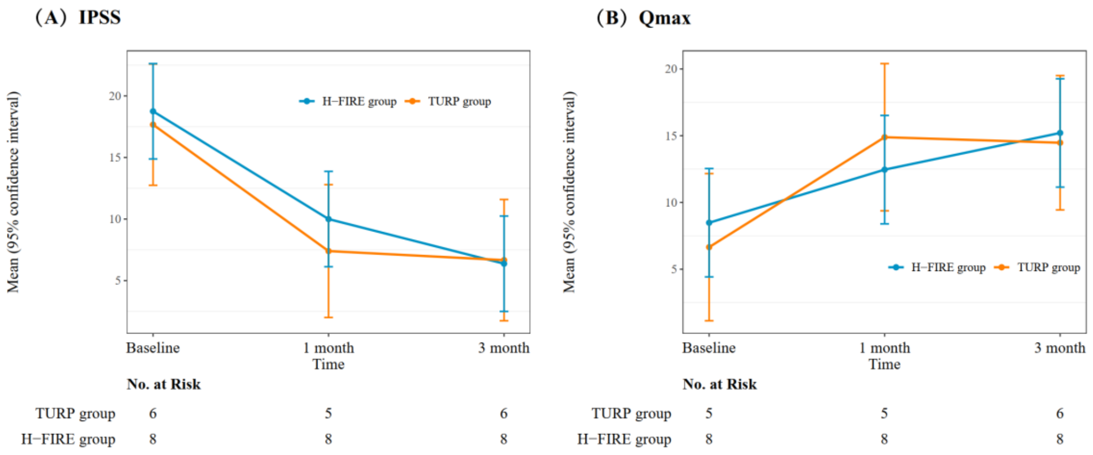

Figure S10. Longitudinal changes in primary outcomes over the follow-up period in patients with bladder calculi at baseline.

(A) Changes in the International Prostate Symptom Score (IPSS) from baseline to post-operative follow-up. **(B)** Changes in the maximum urinary flow rate (Qmax) from baseline to post-operative follow-up. The blue line represents the High-Frequency Irreversible Electroporation (H-FIRE) group, and the orange line represents the Transurethral Resection of the Prostate (TURP) group. Data points indicate mean values, and error bars represent 95% confidence intervals. The tables below the graphs show the number of participants evaluated at each time point.

Note that the number of evaluable patients for Qmax may increase at follow-up compared to baseline, as patients with acute urinary retention (AUR) at baseline were unable to provide a valid measurement (recorded as NA) until after surgical intervention.

Supplementary Table 1. Baseline Clinical Participant Characteristics with Group Comparisons

**Characteristics**	H-Fire Group (n=59)	TURP Group (n=59)	*P*
Age, year, Mean+/-SD	69.58±6.44	68.88±5.58	0.532
<70	28(47.46%)	29(49.15%)	0.854
>=70	31(52.54%)	30(50.85%)	
Race, n (%)			1.000
Han	59(100.00%)	59(100.00%)	
Others	0(0)	0(0)	
Body mass index, kg/m^2, Mean+/-SD	**24.44±2.21**	**23.14±2.96**	**0.008**
Comorbidity			
Hypertension	32(54.24%)	24(40.68%)	0.140
Diabetes Mellitus	14(23.73%)	7(11.86%)	0.092
Coronary heart disease	5(8.47%)	0(0.00%)	0.068
Gout	4(6.78%)	1(1.69%)	0.361
Medical treatment of BPH before surgery, n (%)	41(69.49%)	38(64.41%)	0.557
5α-reductase inhibitors	22(37.29%)	20(33.90%)	0.701
α1 -adrenergic receptor antagonists	4(6.78%)	4(6.78%)	>0.999
Combination of both drugs mentioned above	4(6.78%)	4(6.78%)	>0.999
Others	19(32.20%)	18(30.51%)	0.843
Prostate-specific antigen, ng/ml, M (Q1, Q3)	6.99(2.72, 12.90)	3.59(2.16, 9.27)	0.117
Prostate volume, ml			
M (Q1, Q3)	58.44(49.06, 75.51)	58.23(43.66, 77.83)	0.550
<60	30(50.85%)	30(50.85%)	>0.999
>=60	29(49.15%)	29(49.15%)	
Digital rectal examination, n (%)			>0.999
Positive	3(5.08%)	3(5.08%)	
Negative	56(94.92%)	56(94.92%)	
Bladder calculi, n (%)			0.569
Yes	8(13.56%)	6(10.17%)	
No	51(86.44%)	53(89.83%)	
Urinary catheterization before surgery, n (%)			0.701
Yes	20(33.90%)	22(37.29%)	
No	39(66.10%)	37(62.71%)	
Preoperative clinical characteristics			
Qmax, mL/s, Mean+/-SD	7.90±3.08	7.28±3.44	0.330
IPSS, points, Mean+/-SD	19.83±5.78	19.64±5.02	0.852
IIEF, points, M (Q1, Q3)	2.00(0.00, 19.00)	3.00(0.00, 16.00)	0.528
ICIQ- MLUTSsex, points, M (Q1, Q3)	5.00(2.00, 6.00)	5.00(2.00, 6.00)	0.969
PVRU, ml, M (Q1, Q3)	40.00(5.00, 110.00)	39.00(5.00, 170.00)	0.873

**Characteristics**	H-Fire Group (n=59)	TURP Group (n=59)	*P*
Voided Volume, ml, M (Q1, Q3)	116.94(81.34, 193.14)	104.15(66.60, 153.76)	0.125
ICIQ for urinary incontinence, points			0.229
Free	51(86.44%)	46(77.97%)	
>=1	8(13.56%)	13(22.03%)	
EPIC pad-use, n(%)			>0.999
Free	58(98.31%)	57(96.61%)	
>=1	1(1.69%)	2(3.39%)	
HADS - depression, points, M (Q1, Q3)	1.00(0.00, 3.00)	1.00(0.00, 4.00)	
HADS - anxiety, points, M (Q1, Q3)	0.00(0.00, 2.00)	0.00(0.00, 1.00)	
Hemoglobin, g/L, Mean+/-SD	139.07±14.75	141.07±14.87	0.468
Serum sodium, mmol/L, Mean+/-SD	**138.98±3.24**	**140.55±2.55**	**0.004**

M (Q1, Q3), median (the first quartile, the third quartile); SD, standard deviation; Qmax, maximum urinary flow rate; IPSS, International Prostate Symptom Score; IIEF, International Index of Erectile Function; ICIQ, International Consultation on Incontinence Questionnaire; ICIQ-MLUTSsex, Incontinence Questionnaire Male Sexual Matters Associated with Lower Urinary Tract Symptoms Module; PVRU, Post-Void Residual Urine Volume; EPIC, Expanded Prostate Cancer Index Composite; QoL, Quality of Life; HADS, Hospital Anxiety and Depression Scale;

Supplementary Table 2 Sensitivity analysis for efficacy analysis on primary and secondary outcomes

Outcomes	H-FIRE Group (n=59)		TURP Group (n=59)		*Primary analysis* [a]		***Sensitivity analysis*** [b]	
	n	Estimation (95% CI)	n	Estimation (95% CI)	Difference (95% CI)	*P*	**Difference (95% CI)**	***P***
**Primary outcomes**								
1. Change in Qmax, mL/s [a]								
Intention-to-treat population	59	7.95(6.04, 9.86)	59	7.84(5.94, 9.75)	0.11(-2.52, 2.73)	0.001	**0.18(-2.69, 3.05)**	**0.002**
Per-protocol population	52	7.81(5.80, 9.81)	46	7.95(5.81, 10.09)	-0.15(-3.08, 2.79)	0.005	**0.22(-2.99, 3.42)**	**0.005**
2. Change in IPSS, Points [a]								
Intention-to-treat population	59	-13.24(-14.37, -12.12)	59	-13.75(-14.87, -12.63)	0.50(-1.09, 2.10)	<0.001	**0.35(-1.36, 2.05)**	**0.001**
Per-protocol population	52	-12.93(-14.12, -11.73)	46	-13.54(-14.81, -12.27)	0.62(-1.13, 2.36)	<0.001	**0.33(-1.55, 2.22)**	**0.003**

Qmax, maximum urinary flow rate; IPSS, International Prostate Symptom Score;

Primary outcomes were analyzed in both the Full Analysis Set (FAS) and Per-Protocol Set (PPS). For Qmax (larger is better), the non-inferiority margin is -4 mL/s. For IPSS (smaller is better), the margin is 3 points.

[a] adjusted for the baseline value and two stratification factors (age group: < 70 years, >=70 years; prostate volume: < 60 ml, >=60 ml).

[b] Statistically significant imbalances in body mass index and serum sodium at baseline were observed in group comparison, the two baseline characteristics were further adjusted as a sensitivity analysis.

Supplementary Table 3: Breakdown of IPSS Scores by Voiding and Storage Subdomains at 3 months

IPSS	Overall [a]	TURP Group [a]	H-FIRE Group [a]	Median Difference (95% CI) [b]
Voiding domain	1.0(0.0, 3.0)	1.0(0.0, 3.50)	1.0(0.0, 2.0)	0.50(0.0, 1.0)
Item 1	0.0(0.0, 1.0)	0.0(0.0, 1.0)	0.0(0.0, 1.0)	0.0(0.0, 0.0)
Item 3	0.0(0.0, 1.0)	0.0(0.0, 1.0)	0.0(0.0, 1.0)	0.0(0.0, 0.0)
Item 5	0.0(0.0, 1.0)	0.0(0.0, 1.0)	0.0(0.0, 1.0)	0.0(0.0, 0.0)
Item 6	0.0(0.0, 0.0)	0.0(0.0, 0.0)	0.0(0.0, 0.0)	0.0(0.0, 0.0)
Storage domain	3.0(2.0, 6.0)	3.0(2.0, 5.5)	4.0(2.0, 6.0)	-0.5(-1.0, 0.0)
Item 2	1.0(0.0, 2.0)	1.0(0.0, 2.0)	1.0(0.0, 2.0)	-0.5(-1.0, 0.0)
Item 4	0.0(0.0, 2.0)	0.0(0.0, 2.0)	1.0(0.0, 1.0)	0.0(0.0, 0.0)
Item 7	2.0(1.0, 3.0)	2.0(1.0, 3.0)	2.0(1.0, 3.0)	0.0(0.0, 0.0)

CI, confidence interval.

IPSS items: Item 1, incomplete emptying; Item 2, frequency; Item 3, intermittency; Item 4, urgency; Item 5, weak stream; Item 6, straining; Item 7, nocturia.

[a] Data was presented as median (the first quartile, the third quartile).

[b] Confidence interval was estimated using Hodges-Lehmann method.

Supplementary Table 4 Detailed Analysis of Erectile Function (lIEF-5) at 3 month

IIEF-5	Overall [a]	TURP Group [a]	H-FIRE Group [a]	Median Difference (95% CI) [b]
Item1	1.0(0.0, 3.0)	1.0(0.0, 3.0)	0.50(0.0, 3.0)	0.0(0.0, 0.0)
Item 2	0.0(0.0, 1.0)	0.0(0.0, 1.0)	0.0(0.0, 0.50)	0.0(0.0, 0.0)
Item 3	0.0(0.0, 1.0)	0.0(0.0, 1.0)	0.0(0.0, 0.50)	0.0(0.0, 0.0)
Item 4	0.0(0.0, 1.0)	0.0(0.0, 3.0)	0.0(0.0, 0.50)	0.0(0.0, 0.0)
Item 5	0.0(0.0, 1.0)	0.0(0.0, 1.0)	0.0(0.0, 0.50)	0.0(0.0, 0.0)

CI, confidence interval.

IIEF-5 items: Item 1, erectile confidence; Item 2, erection firmness (frequency); Item 3, maintenance frequency (after penetration); Item 4, maintenance difficulty (to completion of intercourse); Item 5, intercourse satisfaction.

[a] Data was presented as median (the first quartile, the third quartile).

[b] Confidence interval was estimated using Hodges-Lehmann method.

Supplementary Table 5 missing pattern in intention-to-treat set (n=118).

Pattern	Age	Prostate volume	Qmax at baseline	Qmax at 1 month	Qmax at 3 month	IPSS at baseline	IPSS at 1 month	IPSS at 3 month	N	Percent
TURP group										
1	X	X	X	X	X	X	X	X	45	76.27
2	X	X	X	X	.	X	X	.	3	5.08
3	X	X	X	.	X	X	.	X	1	1.69
4	X	X	X	.	.	X	.	.	2	3.39
5	X	X	.	X	X	X	X	X	7	11.86
6	X	X	.	.	.	X	.	.	1	1.69
H-FIRE group										
1	X	X	X	X	X	X	X	X	50	84.75
2	X	X	X	X	X	X	.	X	1	1.69
3	X	X	X	X	.	X	X	.	4	6.78
4	X	X	X	.	X	X	X	X	1	1.69
5	X	X	X	.	.	X	.	.	1	1.69
6	X	X	.	X	X	X	X	X	1	1.69
7	X	X	.	.	X	X	.	X	1	1.69

An “X” represents observed data and a “.” represent missing data.

Q_{max}, maximum urinary flow rate; IPSS, International Prostate Symptom Score;

Supplementary Table 6. Cross-tabulation of patients' perceived versus actual treatment allocation at 3 months post-surgery.

	Patient's Guess			
Actual Allocation	Guessed H-FIRE	Guessed TURP	Don't Know	Total
H-FIRE Group	12 (20.3%)	9 (15.3%)	38 (64.4%)	59
TURP Group	8 (13.6%)	14 (23.7%)	37 (62.7%)	59
Total	20	23	75	118

Footnotes:

Data are presented as No. (%). The blinding assessment was conducted via a questionnaire at the 3-month follow-up visit prior to unblinding. The high proportion of "Don't Know" responses (>60% in both groups) indicates successful maintenance of blinding.

Regarding the reasons for specific guesses (n=43), participants cited catheterisation duration (n=24), sexual function (n=14), and bleeding/hematuria (n=12) as key factors. Notably, "catheterisation duration" was cited as a reason for guessing by participants in both the H-FIRE group (n=12) and the TURP group (n=12), suggesting that the unified preoperative counselling successfully prevented this symptom from serving as a definitive unblinding factor.

Supplementary Table 7: Independent Assessment of Transurethral Resection of the Prostate (TURP) Quality

This table presents the blinded assessments of TURP procedural quality by three independent urological surgeons. Videos were randomly selected from consecutive blocks of procedures. Assessments were based on a 5-point Likert scale (1 = Poor, 2 = Suboptimal, 3 = Adequate, 4 = Good, 5 = Excellent).

Abbreviations: R1, Reviewer 1; R2, Reviewer 2; R3, Reviewer 3.

Patient ID	Overall Score	Resection Completeness	Channel Patency / Anatomical Shape	Hemostasis	Recognition of Surgical Landmarks	Tissue Removal Efficiency
	R1 / R2 / R3	R1 / R2 / R3	R1 / R2 / R3	R1 / R2 / R3	R1 / R2 / R3	R1 / R2 / R3
**Patient 2**	5 / 5 / 4	5 / 5 / 4	5 / 5 / 4	4 / 5 / 4	5 / 5 / 5	5 / 5 / 5
**Patient 5**	5 / 5 / 5	5 / 5 / 4	5 / 5 / 5	5 / 4 / 4	5 / 5 / 5	5 / 5 / 5
**Patient 13**	5 / 5 / 5	5 / 5 / 5	5 / 5 / 5	5 / 5 / 5	5 / 5 / 5	5 / 5 / 5
**Patient 17**	5 / 5 / 5	5 / 5 / 5	5 / 5 / 5	5 / 4 / 4	5 / 5 / 5	4 / 5 / 5
**Patient 20**	4 / 4 / 4	4 / 5 / 4	5 / 4 / 5	4 / 4 / 4	5 / 5 / 5	5 / 5 / 5

Patient ID	Overall Score	Resection Completeness	Channel Patency / Anatomical Shape	Hemostasis	Recognition of Surgical Landmarks	Tissue Removal Efficiency
**Patient 28**	4 / 5 / 4	4 / 5 / 5	5 / 5 / 5	3 / 4 / 3	5 / 5 / 5	5 / 5 / 5
**Patient 32**	4 / 5 / 4	4 / 5 / 4	5 / 5 / 5	4 / 5 / 4	5 / 5 / 5	4 / 5 / 5
**Patient 35**	5 / 5 / 5	5 / 5 / 5	5 / 5 / 5	5 / 5 / 5	5 / 5 / 5	5 / 5 / 5
**Patient 43**	5 / 5 / 5	5 / 5 / 5	5 / 5 / 5	5 / 5 / 5	5 / 5 / 5	4 / 5 / 5
**Patient 47**	4 / 4 / 4	5 / 5 / 5	5 / 5 / 5	4 / 5 / 4	4 / 4 / 5	5 / 5 / 4
**Patient 49**	4 / 5 / 4	5 / 5 / 5	5 / 5 / 5	3 / 4 / 3	4 / 5 / 4	5 / 5 / 5
**Patient 58**	4 / 5 / 4	5 / 5 / 5	5 / 5 / 4	4 / 4 / 4	4 / 5 / 5	5 / 5 / 5
**Mean Score (SD)**	4.6 (0.5)	4.8 (0.5)	4.9 (0.3)	4.3 (0.7)	4.8 (0.4)	4.9 (0.3)

Interpretation: The consistently high scores across all patients and assessment criteria demonstrate that the TURP procedures performed in the GIANT trial were

executed to a high technical standard. The lowest mean score was observed in the 'Hemostasis' category, though it still resided in the 'Good' range. The high degree of inter-rater agreement, with most scores varying by no more than one point between reviewers, supports the reliability of these quality assessments.
