# Supplementary material for: High-frequency irreversible electroporation versus transurethral resection of the prostate for benign prostatic hyperplasia (GIANT): a single-centre, randomised, double-blind, phase 3, non-inferiority trial
Source: eClinicalMedicine. 2026 Jul 2;97:104034. doi: 10.1016/j.eclinm.2026.104034 (PMC13352031; doi:10.1016/j.eclinm.2026.104034)
Supplement: Protocol [file mmc2.pdf]

GIANT — Guided Irreversible electroporation Aim to Narrow down Thick prostate: A single-centre non-inferior double-blinded randomized controlled study evaluating the efficacy and safety of high-frequency irreversible electroporation in treating lower urinary tract symptoms and benign prostatic obstruction

GIANT

Guided Irreversible electroporation
Aim to Narrow down Thick prostate

STUDY PROTOCOL

V3.0 (2023-01-24)

PROTOCOL VERSION

Version number	Approval Date
1.0	Mar 20, 2022
2.0	April 25, 2022
3.0	Jan 24, 2023

PROTOCOL SUMMARY

**Title**	A single-centre non-inferior double-blinded randomized controlled study evaluating the efficacy and safety of high-frequency irreversible electroporation in treating lower urinary tract symptoms and benign prostatic obstruction
**Acronym**	GIANT
**Study design**	GIANT is a prospective, investigator-initiated, single-centre, randomised controlled, double-blinded and non-inferiority study in which men with lower urinary tract symptoms and benign prostatic obstruction
**Trial identifier**	ClinicalTrials.gov: NCT05306145
**Trial Sponsor**	Shanghai East Hospital, School of Medicine, Tongji University
**Clinical site location**	Shanghai, China
**Study centre**	Shanghai East Hospital
**Study objective**	To evaluate whether the high-frequency irreversible electroporation is non-inferior to Transurethral resection of the prostate in treating lower urinary tract symptoms and benign prostatic obstruction
**Subject population**	Subjects with lower urinary tract symptoms and benign prostatic obstruction (LUTS/BPO) will be considered eligible for registration in this trial
**Number of trial subjects**	Approximately 118 subjects will be enrolled.
**Inclusion criteria**	1. Age over 40 years old.<br>2. IPSS>8.<br>3. Q_{max} <15ml/s.<br>4. Prostatic volume range of 30 to 100ml, measured by MRI.

	5. Fully understand the clinical trial protocol and sign the informed consent.
**Exclusion criteria**	1. Have a history of prostate cancer or patients suspicious of prostate cancer.<br>2. Neurogenic bladder<br>3. Metal implants in their body<br>4. Previous history of prostatic or urethral surgery<br>5. With Catheterisation more than 2 weeks.<br>6. Any other conditions that make the investigator judge that participants are not suitable for this trial.
**Randomization ratio**	1:1
**Arms**	There are two arms in the trial:<br>Arm1: High-frequency irreversible electroporation (H-FIRE)<br>Arm2: Transurethral resection of the prostate (TURP)
**Primary outcome**	The co-primary outcome is to assess the change from baseline in maximal flow rate (Q_{max}) and urinary symptoms by questionnaire of International Prostate Symptom Score (IPSS) scoring at 3 months after surgical treatment.
**Secondary outcomes**	1. To assess the improvement from baseline in erectile symptoms by the 5-item version of the International Index of Erectile Function (IIEF-5) scoring and the International Consultation on Incontinence Questionnaire Male Sexual Matters Associated with Lower Urinary Tract Symptoms Module (ICIQ-MLUTSsex) at 3 months after surgical treatment.<br>2. To assess the change from baseline in post-void residual urine volume (PVRU) at 3 months after surgical treatment.<br>3. To assess the change from baseline in voided volume at

	3 months after surgical treatment.<br>4.To assess the change from baseline in the urinary incontinence by ICIQ (International Consultation on Incontinence Questionnaire) score and separate EPIC (Expanded Prostate Cancer Index Composite) pad-use item at 3 months after surgical treatment.<br>5.To assess the change from baseline in the quality of life (QOL) by IPSS QoL subscore[1] and Hospital Anxiety and Depression Scale (HADS) at 3 months after surgical treatment.<br>6.To assess the perioperative parameters, including operative time, the postoperative hospital stay, haemoglobin declination, serum sodium declination, and catheterisation duration.<br>7.To assess the early postoperative urinary symptoms include dysuria, urgency, or postmicturition pain.<br>8.To assess the change from baseline in the pain at 3 months after surgical treatment.<br>9.To assess the adverse event at every visit. It mainly includes transurethral resection syndrome, blood transfusion, clot retention, urinary tract infection (UTI), fever and other adverse events according to the Common Terminology Criteria for Adverse Events (CTCAE).
**Analysis**	The primary outcome in this trial will be analysed following the intention-to-treat principle as well as the per-protocol principle. The difference between the two groups will be evaluated with a 95% confidence interval (CI) by using the generalised linear mix model. The H-FIRE will be described as non-inferior if the lower bound of the 95%CI of the

	difference in the Q_{max} and IPSS of the H-FIRE arm compared with the TURP arm (H-FIRE arm minus TURP arm) is higher than −4mL/s and 3 points(non-inferiority margin).<br>The second outcome will be appropriately analysed for the different distribution (e.g., t-test, Pearson chi-square test, etc.) and described with 95%CI. Each reported P value in this trial will be two-sided.
**Status**	Now recruiting

TABLE OF CONTENTS

1. Title........10
   1.1. Full title: 10
   1.2. Short title: 10
   1.3. Acronym: 10
2. Trial identifier 10
3. Trial Sponsor 10
4. Study organization 10
   4.1. Chief investigator: 11
   4.2. Co-Chief investigator: 11
   4.3. Co-Investigator and GIANT members: 11
5. Support 13
6. ABBREVIATIONS 14
7. Abstract 16
8. Introduction 17
9. Trial design 18
   9.1. Design overview 18
   9.2. Trial aims 20
   9.3. Trial hypotheses 20
   9.4. Timeframe 21
10. Outcomes 22
   10.1. Primary outcome 22
   10.2. Secondary outcomes 23
11. Methods and analysis 26
   11.1. Patient population 26
   11.1.1 Inclusion criteria 27
   11.1.2. Exclusion criteria 27
   11.2. Randomization and blinding 28
   11.2.1. Randomization 28
   11.2.2. Blinding 29
   11.3. Interventions 30

11.3.1 Baseline characteristics 30
11.3.2. Surgery procedure 31
11.3.2.1 H-FIRE strategy 31
11.3.2.2 TURP strategy 32
11.3.3 After surgery 33
11.4. Follow-up 33
11.5. Withdrawal 34
11.5.1. Reasons for withdrawal 34
11.5.2. Handlement for withdrawal 34
12. Analysis plan 34
12.1. Sample size 35
12.2. Statistical analysis 37
12.2.1. Primary outcome 37
12.2.2. Secondary outcomes 37
12.2.3. Pre-specified subgroup analysis 38
12.2.4. Safety analysis 38
13. Harms and adverse events 39
13.1. Expected harms and adverse events 39
13.2. Serious adverse events 40
14. Data collection 40
14.1. Case Report Forms 41
14.2. Data to be collected on Screening and Inclusion 41
14.3. Data to be collected during hospitalization 41
14.4. Data to be collected during follow up 42
14.5. Data retention 42
15. Monitoring 42
15.1. Independent team of clinical research associate 42
15.2. Data Safety Monitoring Board 43
16. Quality control 43
16.1.Surgical Quality Assurance and Control 43
16.1.1 Procedural Documentation and Assessment of H-FIRE 44
16.1.2 Quality Assessment of TURP 45

16.2.Quality Control of Randomization Procedures 46
16.3.Standardization of Patient Assessment and Data Collection 46
16.4.Data Management and Integrity Control 47
16.5.Endpoint Adjudication Committee 47
17. Ethics and dissemination 48
18. Authors' contributions 49
19. Declaration of interests 49
20. Protocol Amendments 49
20.1. Original protocol (V1.0) (March-2022) 49
20.2. Protocol V2.0 (April-2022) 49
20.3. Protocol V3.0 (Jan-2023) 58
20. References 60
Appendix 1: International Prostate Symptom Score (IPSS) 62
Appendix 2: 5-item version of the International Index of Erectile Function (IIEF-5) 63
Appendix 3: International Consultation on Incontinence Questionnaire Male Sexual Matters Associated with Lower Urinary Tract Symptoms Module (ICIQ-MLUTSsex) 64
Appendix 4: International Consultation on Incontinence Questionnaire (ICIQ) 65
Appendix 5: Expanded Prostate Cancer Index Composite (EPIC) pad-use item 66
Appendix 6: Hospital Anxiety and Depression Scale (HADS) 67
Appendix 7: Early postoperative urinary symptoms assessment 69
Appendix 8: Surgical Pain Scale 70
Appendix 9 Common Terminology Criteria for Adverse Events (CTCAE) 71
Appendix 10 TURP Technical Quality Assessment Questionnaire 72

1.Title

1.1. Full title:

A single-centre non-inferior double-blinded randomized controlled study evaluating the efficacy and safety of high-frequency irreversible electroporation in treating lower urinary tract symptoms and benign prostatic obstruction

1.2. Short title:

Guided Irreversible electroporation Aim to Narrow down Thick prostate

1.3. Acronym:

GIANT

2. Trial identifier

ClinicalTrials.gov: NCT05306145

3. Trial Sponsor

Shanghai East Hospital, School of Medicine, Tongji University, No. 150, Jimo Road, Shanghai, China

4. Study organization

The study organization comprise the chief investigator, the chief coordinator and the trial working group (GIANT group). The chief investigator will decide on the plan and the execution of the study alone. The GIANT group can advise the chief investigator.

4.1. Chief investigator:

Professor Hai-Feng Wang

Professor of Urology

Shanghai East Hospital, School of Medicine, Tongji University

E-mail: kuohaiandrew2000@vip.sina.com; 446720864@qq.com

TEL: +86-13681750891

4.2. Co-Chief investigator:

Mr Bi-Ming He

Residency of Urology

Shanghai East Hospital, School of Medicine, Tongji University

E-mail: 190589109@qq.com

TEL: +86-15502139410

4.3. Co-Investigator and GIANT members:

**Steering committee**		
Hai-Feng Wang	Urologist	Department of urology, Shanghai East Hospital, School of Medicine, Tongji University
Bi-Ming He	Urologist	Department of urology, Shanghai East Hospital, School of Medicine, Tongji University
Rong-Bing Li	Urologist	Department of urology, Shanghai East Hospital, School of Medicine, Tongji University
Li-Qun Huang	Urologist	Department of urology, Shanghai East Hospital, School of Medicine, Tongji

		University
Dong-Yang Li	Urologist	Department of urology, Shanghai East Hospital, School of Medicine, Tongji University
Zhen-Kai Shi	Urologist	Department of urology, Shanghai East Hospital, School of Medicine, Tongji University
Shuai-Dong Wang	Urologist	Department of urology, Shanghai East Hospital, School of Medicine, Tongji University
Qi-Wei Yang	Urologist	Department of urology, Shanghai East Hospital, School of Medicine, Tongji University
**Statistical Committee**		
Zhi-Chao Jin	Statistician	Department of Health Statistics, Naval Medical University
Yan-Fang Zhao	Statistician	Department of Health Statistics, Naval Medical University
**Data Safety Monitoring Board**		
Lin-Hui Wang	Urologist	Department of Urology, Changhai Hospital, Naval Medical University
Jia He	Statistician	Department of Health Statistics, Naval Medical University
Jin Li	Oncologist	Department of medical oncology, Shanghai East Hospital, School of Medicine, Tongji University
**Endpoint Adjudication Committee**		
Yong-Da Liu	Urologist	Department of Urology, The First

		Affiliated Hospital of Guangzhou Medical University, Guangzhou Medical University
Yi-Feng Jing	Urologist	Department of Urology, Shanghai General Hospital, Shanghai Jiao Tong University School of Medicine
Xiao-Feng Gao	Urologist	Department of Urology, Changhai Hospital, First Affiliated Hospital of Naval Medical University
**Independent Quality Control Group for the Surgery procedure**		
Yong-Han Peng	Urologist	Department of Urology, Changhai Hospital, First Affiliated Hospital of Naval Medical University
Yi-Sen Meng	Urologist	Department of Urology, Peking University First Hospital
Qing Yuan	Urologist	Department of Urology, Third Center of the Chinese People's Liberation Army General Hospital

5. Support

This trial is supported by National key research and development program(2019YFC0119100) from National Natural Science Foundation of China and supported by Shanghai Science and Technology Commission Foundation (18441910900) from Science and Technology Commission of Shanghai Municipality. The study sponsor or funder had no authority over study design, management, execution, data collection, analysis, manuscript writing, outcome disclosing, and dissemination or publishing.

6.ABBREVIATIONS

BPH	benign prostatic hyperplasia
LUTS	lower urinary tract symptoms
BPO	benign prostatic obstruction
QOL	quality of life
TURP	transurethral resection of the prostate
Q_{max}	maximal flow rate
IPSS	international prostate symptom score
IRE	irreversible electroporation
H-FIRE	High-frequency irreversible electroporation
RCT	randomised controlled trial
PSA	prostate-specific antigen
MRI	magnetic resonance imaging
IIEF-5	5-item version of the International Index of Erectile Function
ICIQ	International Consultation on Incontinence Questionnaire
MLUTSsex	Male Sexual Matters Associated with Lower Urinary Tract Symptoms Module
PVRU	post-void residual urine volume
EPIC	Expanded Prostate Cancer Index Composite
HADS	Hospital Anxiety and Depression Scale
UTI	urinary tract infection
CTCAE	Common Terminology Criteria for Adverse Events
SAP	statistical analysis plan
CRF	case report form
AE	adverse events
SAE	severe adverse events
CI	confidence interval

CRA	clinical research associate
DSMB	Data Safety Monitoring Board

7. Abstract

Transurethral resection of the prostate (TURP) is the gold standard surgical treatment to lower urinary tract symptoms and benign prostatic obstruction (LUTS/BPO). Although it has been proven to have substantial efficacy in improving functional outcomes, it has shown a high incidence of complications, including transurethral resection syndrome, massive bleeding, urinary incontinence, and sexual dysfunction. High-frequency irreversible electroporation (H-FIRE) is a novel nonthermal ablation technique that delivers pulsed high-voltage but low-energy electric current to the cell membrane, thereby leading to cell death. H-FIRE has been reported to be tissue-selective, which leads to fewer side effects. However, no data are available on whether H-FIRE is non-inferior compared with TURP in treating patients with LUTS/BPO regarding safety and efficacy.

This trial is a prospective, single-centre, randomised controlled, double-blinded and non-inferiority study in which all men with LUTS/BPO are included. This study aims to determine whether the H-FIRE is non-inferior to TURP for achieving better functional outcomes as measured by the maximal flow rate (Q max). The main inclusion criteria are men with prostatic volume range 30 to 100ml, the Qmax<15ml/s, and the IPSS>8. A sample size of 118 participants is required, accounting for a 20% loss. All participants will be randomly allocated at a ratio of 1:1 to the H-FIRE arm (n = 59) and the TURP arm (n = 59). The primary outcome is to assess the change from baseline in maximal flow rate (Q_{max}) and urinary symptoms by questionnaire of International Prostate Symptom Score (IPSS) scoring at 3 months after surgical treatment.

8. Introduction

Benign prostatic hyperplasia (BPH) is one of the most common diseases in men over age 50[2]. It is often associated with lower urinary tract symptoms (LUTS) and benign prostatic obstruction (BPO), which make a significant impact on quality of life (QOL) and bring a substantial economic burden. Transurethral resection of the prostate (TURP), using the electrode to resect the enlarged prostatic tissue piece by piece, was the preferred surgical treatment for LUTS/BPO in the 1970s and remained the gold standard to date[3]. It has been proven to have substantial efficacy in improving functional outcomes like the maximal flow rate (Q_{max}), the urinary symptoms, which are assessed by the international prostate symptom score (IPSS), and the QOL[4 5]. Despite its promising efficacy, TURP is associated with several complications and side effects such as transurethral resection syndrome or massive bleeding, which may extend hospital stay and increase the treatment costs, as well as the urinary incontinence or sexual dysfunction[6]. Thus, finding and utilising another minimally invasive treatment is necessary to achieve a similar functional outcome while reducing the limitations.

Irreversible electroporation (IRE) is a novel nonthermal technique for ablation that utilises pulsed, high voltage but the low-energy direct electric current to induce nanometer-scale pores in the cell membrane, thereby leading to cell death[7]. Due to the independence of thermal processes, IRE seems to be tissue-selective with minor damage to the connective tissue structure, such as blood vessels or nerves, whereas the thermal ablative techniques may cause collateral damage or be limited the heat-sink effect[8 9]. Typical IRE protocols utilise 80 to 120 unipolar pulses with a pulse of 50 to 100 ms, which may evoke muscle contraction during the procedure and then cause the deviation of the set ablation area position. Another type of IRE technique called high-frequency

IRE (H-FIRE) has emerged to overcome this limitation. The H-FIRE protocol applies a set of bipolar pulse bursts, consisting of several individual 0.5 to 10 us pulse, aiming to reduce the muscle contraction[10].

Although H-FIRE and IRE have been used to treat a malignant tumour in most cases, they have the promising potential to treat benign diseases like BPH attributed to their efficacy and safety. In our previous multicenter single-arm objective performance criteria trial in treating localised prostate cancer by H-FIRE, we found that in addition to tumour control, it can significantly improve the functional outcome of LUTS/BPO[11]. The IPSS increased by 50.0% six months after H-FIRE treatment. At the same time, it has not had many complications, especially a very small influence on the erectile symptoms and urinary continence. Based on these data and evidence, we hypothesise that H-FIRE might achieve a similar functional outcome but fewer side effects when compared to the TURP.

Hence, we are conducting this single-centre randomised controlled trial (RCT) to confirm our hypothesis. The primary objective of this trial is to assess whether the H-FIRE is non-inferior to TURP in improving functional outcomes as measured by the Q_{max} and IPSS in patients with LUST/BPO.

9. Trial design

9.1. Design overview

GIANT is a prospective, investigator-initiated, single-centre, randomised controlled, double-blinded and non-inferiority study anticipated to enrol 118 patients with symptomatic benign prostatic hyperplasia. Participants will be randomised to the high-frequency IRE (H-FIRE) group or the transurethral

resection of the prostate (TURP) group. The primary objective of this study is to determine whether the H-FIRE is non-inferior to the TURP for achieving better functional outcomes.

The design overview chart is shown in below.

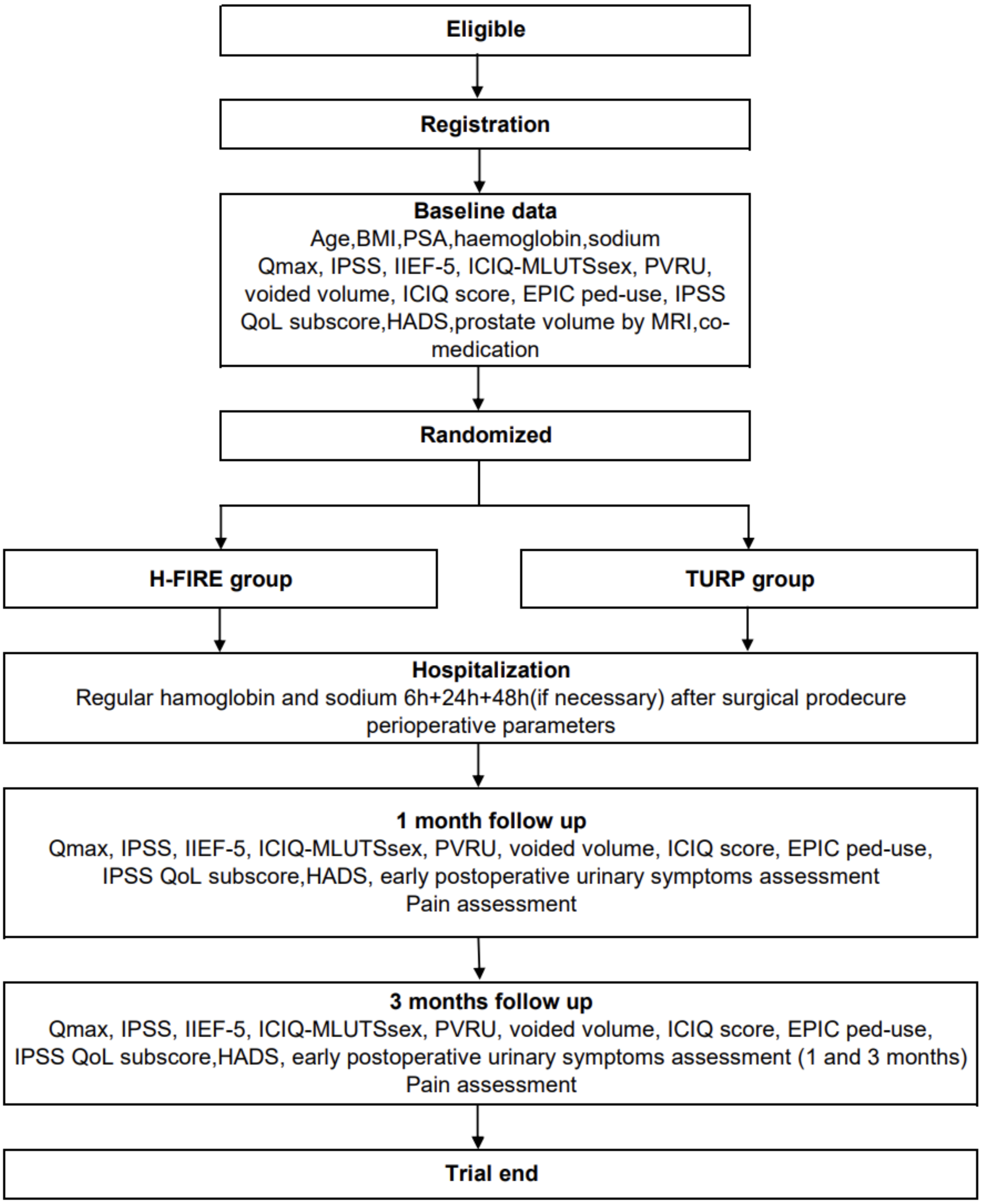

9.2. Trial aims

Aim 1. Compare clinical outcomes in patients randomized to H-FIRE versus TURP.

Sub Aim 1. Compare clinical outcomes in patients without urinary retention randomized to the H-FIRE or TURP strategy.

Aim 2. Compare patient reported outcomes (PROs) in patients randomized to H-FIRE versus TURP.

Sub Aim 2. Compare PROs in patients without urinary retention randomized to the H-FIRE or TURP strategy.

9.3. Trial hypotheses

The central hypothesis of the GIANT trial is that clinical outcomes and PROs among participants in the H-FIRE arm will not be inferior to those in the TURP arm.

The sub-hypotheses are shown as follows:

►Sub hypotheses 1: The urinary symptoms among participants in the H-FIRE arm will not be inferior to those in the TURP arm

►Sub hypotheses 2: The erectile symptoms among participants in the H-FIRE arm will be superior to those in the TURP arm

►Sub hypotheses 3: The urinary continence among participants in the H-FIRE arm will be superior to those in the TURP arm

►Sub hypotheses 4: The post-void residual urine volume and voided volume among participants in the H-FIRE arm will not be inferior to those in the TURP arm

►Sub hypotheses 5: The quality of life among participants in the H-FIRE arm will be superior to those in the TURP arm

9.4. Timeframe

Table1. Participant timeline in the study

	Contact with patient			
	-1~0 month	0	1 month	3 months
Consent	×			
fulfill inclusion criteria	×			
Baseline characteristic	×			
PSA	×	×		
MRI	×			×
Randomisation	×			
H-FIRE procedure(H-FIRE arm)		×		
TURP prodecure (TURP arm)		×		
Qmax	×		×	×
IPSS	×		×	×
IIEF-5	×		×	×
ICIQ-MLUTSsex	×		×	×
PVRU	×		×	×
ICIQ score	×		×	×
EPIC ped-use item	×		×	×
IPSS QoL subscore	×		×	×
HADS	×		×	×
Pain assessment*	×	×	×	×
haemoglobin	×			

serum sodium	×			
co-medication	×		×	×
perioperative parameters		×		
early postoperative urinary symptoms assessment			×	×
Withdrawal	Complete as required at any time following registration			
AE and SAE	Complete as required at any time following registration			

PSA = prostate specific antigen; MRI = magnetic Resonance Imaging; H-FIRE = high-frequency irreversible electroporation; TURP = transurethral resection of the prostate; Qmax = maximal flow rate; IPSS = international prostate symptom score; IIEF5= 5-item version of the International Index of Erectile Function; ICIQ-MLUTSsex = International Consultation on Incontinence Questionnaire Male Sexual Matters Associated with Lower Urinary Tract Symptoms Module; RVRU = postvoid residual urine; ICIQ = International Consultation on Incontinence Questionnaire; EPIC = Expanded Prostate Cancer Index Composite; HADS = Hospital Anxiety and Depression Scale; QOL = quality of life; AE = adverse events; SAE = serious adverse events; * : pain assessment will also be followed up by phone at 1 week

10. Outcomes

10.1. Primary outcome

The co-primary outcome is to assess the change from baseline in maximal flow rate (Q_{max}) urinary symptoms by questionnaire of International Prostate Symptom Score (IPSS)[1] scoring at 3 months after surgical treatment.

Definition: Q_{max} is a widely used clinical measure of urodynamics evaluated by the urinary flow rate assessment for benign prostatic obstruction. Patients with bladder catheters will be assessed the Q_{max} after the removal of the catheter.

Q_{max} will be assessed via uroflowmetry. To standardize the assessment across all patients and visits, the following protocol will be strictly adhered to:

►Pre-assessment Preparation: Patients will be instructed to drink water prior to their visit and to delay voiding until they experience a persistent and strong desire to void.

►Measurement: The Q_{max} obtained during a single voluntary void will be recorded as the raw value for that visit.

►Handling of Acute Urinary Retention: If a participant is unable to void despite a persistent and strong desire to do so, and this is confirmed clinically, the event will be recorded as "acute urinary retention". For the purpose of Q_{max} analysis, this will be treated as a missing data point and assigned a value of NA in the dataset.

IPSS is a well-established and validated patient-reported outcome, with 7 items to assess the degree of urinary symptoms during the last 4 weeks, ranging from 0 to 35, with higher scores indicating more severe urinary symptoms (Appendix 1). IPSS will be assessed for those who have removed the bladder catheter after the surgery. If patients still have bladder catheters, IPSS will not be assessed.

10.2. Secondary outcomes

The main secondary outcomes are as follows:

►To assess the improvement from baseline in erectile symptoms by the 5-item version of the International Index of Erectile Function (IIEF-5)[12] scoring and the International Consultation on Incontinence Questionnaire Male Sexual Matters Associated with Lower Urinary Tract Symptoms Module (ICIQ-MLUTSsex)[13] at 3 months after surgical treatment.

Definition: IIEF-5 is a 5-item self-report questionnaire to the presence and severity of erectile dysfunction, ranging from 0 to 25, with higher scores indicating better erectile function (Appendix 2). IIEF-5 will be assessed for those who have removed the bladder catheter after the surgery. If patients still have bladder catheters, IIEF-5 will not be assessed.
ICIQ-MLUTSsex is a self-report questionnaire to assess sexual dysfunction in men with LUTS (Appendix 3). ICIQ-MLUTSsex will be assessed for those who have removed the bladder catheter after the surgery. If patients still with bladder catheters, ICIQ-MLUTSsex will not be assessed.

►To assess the change from baseline in the post-void residual urine volume (PVRU) at 3 months after surgical treatment.
Definition: PVRU is the amount of urine retained in the bladder after a voluntary void and is a clinical measure for assessing benign prostatic obstruction.

►To assess the change from baseline in the voided volume at 3 months after surgical treatment.
Definition: Voided volume is the amount of urine that is a voluntary void and functions as a clinical measure for assessing LUTS.

►To assess the change from baseline in the urinary incontinence by ICIQ (International Consultation on Incontinence Questionnaire)[14] score and separate EPIC (Expanded Prostate Cancer Index Composite)[15] pad-use item at 3 months after surgical treatment.
Definition: ICIQ is a 3-item self-report questionnaire for urinary incontinence, ranging from 0 to 21, with higher scores indicating worse incontinence (Appendix 4). The ICIQ score will be assessed for those who removed the

bladder catheter after the surgery. The ICIQ score will not be assessed if patients still have bladder catheters.

EPIC pad-use item is a self-report questionnaire to record the daily pad use, with higher daily pad use indicating worse incontinence (Appendix 5). EPIC pad-use score will be assessed for those who have removed the bladder catheter after the surgery. If patients still have bladder catheters, EPIC pad-use will not be assessed.

►To assess the change from baseline in the quality of life (QOL) by IPSS QoL subscore[1] and Hospital Anxiety and Depression Scale (HADS)[16] at 3 months after surgical treatment.

Definition: IPSS QoL subscore is a 1-item self-report questionnaire to assess the quality of life with prostate symptoms, ranging from 0 to 6, with higher scores indicating a worse quality of life (Appendix 1). IPSS QoL subscore will be assessed for those who have removed the bladder catheter after the surgery. The IPSS QoL subscore will not be assessed if patients still have bladder catheters.

HADS is a self-assessment scale for evaluating the presence and severity of anxiety and depression, ranging from 0 to 42, with a higher score indicating more distress (Appendix 6).

►To assess the perioperative parameters, including operative time, the postoperative hospital stay, haemoglobin declination, serum sodium declination, and catheterisation duration.

Definition: Operative time is the time of the procedure of H-FIRE or TURP (If the patient has a combined bladder stone, the time for bladder lithotripsy will not be calculated).

Postoperative hospital stay is the length from the first day after surgery to discharge.

Haemoglobin declination is defined as the baseline haemoglobin minus postoperative haemoglobin.
Serum sodium declination is defined as the baseline serum sodium minus postoperative serum sodium.
Catheterisation duration is the length from the first day after surgery to catheterisation. The catheterisation day before surgery will not be calculated.

►To assess the early postoperative urinary symptoms include dysuria, urgency, or postmicturition pain.
Definition: The early postoperative urinary symptoms will be measured by a 5-item self-report questionnaire (Appendix 7).

►To assess the change from baseline in the pain[17] at 3 months after surgical treatment.
Definition: Pain will be assessed by 4-item surgical pain scale, ranging from 0 to 40, higher score indicating more severe pain (Appendix 8).

►To assess the adverse event at every visit. It mainly includes transurethral resection syndrome, blood transfusion, clot retention, urinary tract infection (UTI), fever and other adverse events according to the Common Terminology Criteria for Adverse Events (CTCAE).

11. Methods and analysis

11.1. Patient population

Patients who fulfil all items of inclusion criteria and without any of the exclusion criteria will be considered qualified to register in this trial. The inclusion criteria include age over 40 years, the prostatic volume range of 30 to 100ml, the Q_{max}

<15ml/s, and the IPSS>8. The volunteers will not be recruited if they have a history of prostate cancer or patients suspicious of prostate cancer, neurogenic bladder, metal implants in their body, with long time catheterisation (>2 weeks), or previous history of prostatic or urethral surgery.

Note:

►Q_{max} assessment: Patients will be instructed to drink water prior to their visit and to delay voiding until they experience a persistent and strong desire to void. The Q_{max} obtained during a single voluntary void will be recorded as the raw value for that visit. If a participant is unable to void despite a persistent and strong desire to do so, and this is confirmed clinically, the event will be recorded as "acute urinary retention". For the purpose of Q_{max} analysis, this will be treated as a missing data point and assigned a value of NA in the dataset.

►IPSS assessment: Patients with bladder catheters will be assessed for the IPSS for 4 weeks before urinary retention and bladder catheter insertion.

►Prostate volume assessment: The height (H), width (W), and length (L) of the prostate gland will be measured by the T2-weighted imaging (T2WI) of MRI. The prostate volume will be calculated by the formula: 0.52*H*W*L.

11.1.1 Inclusion criteria

►Age over 40 years old.

► IPSS>8.

► Q_{max} <15ml/s.

► Prostatic volume range of 30 to 100ml, measured by MRI.

► Fully understand the clinical trial protocol and sign the informed consent.

11.1.2. Exclusion criteria

► Have a history of prostate cancer or patients suspicious of prostate cancer.

► Neurogenic bladder

► Metal implants in their body

► Previous history of prostatic or urethral surgery

► With Catheterisation more than 2 weeks.

► Any other conditions that make the investigator judge that participants are not suitable for this trial.

11.2. Randomization and blinding

11.2.1. Randomization

Participants who meet the criteria and sign the consent form will be randomly allocated 1:1 to the H-FIRE arm or TURP arm by using block-randomisation. The random sequence will be generated by a computerised database independent of the investigator and will be stratified by age (<70 yrs and ≥70 yrs) and prostate volume (<60ml, ≥60ml). The randomised number will be revealed after the investigator checks the patient's informed consent and the inclusion and exclusion criteria. Each participant will have an individual randomisation number which will be recorded in the case report form (CRF).

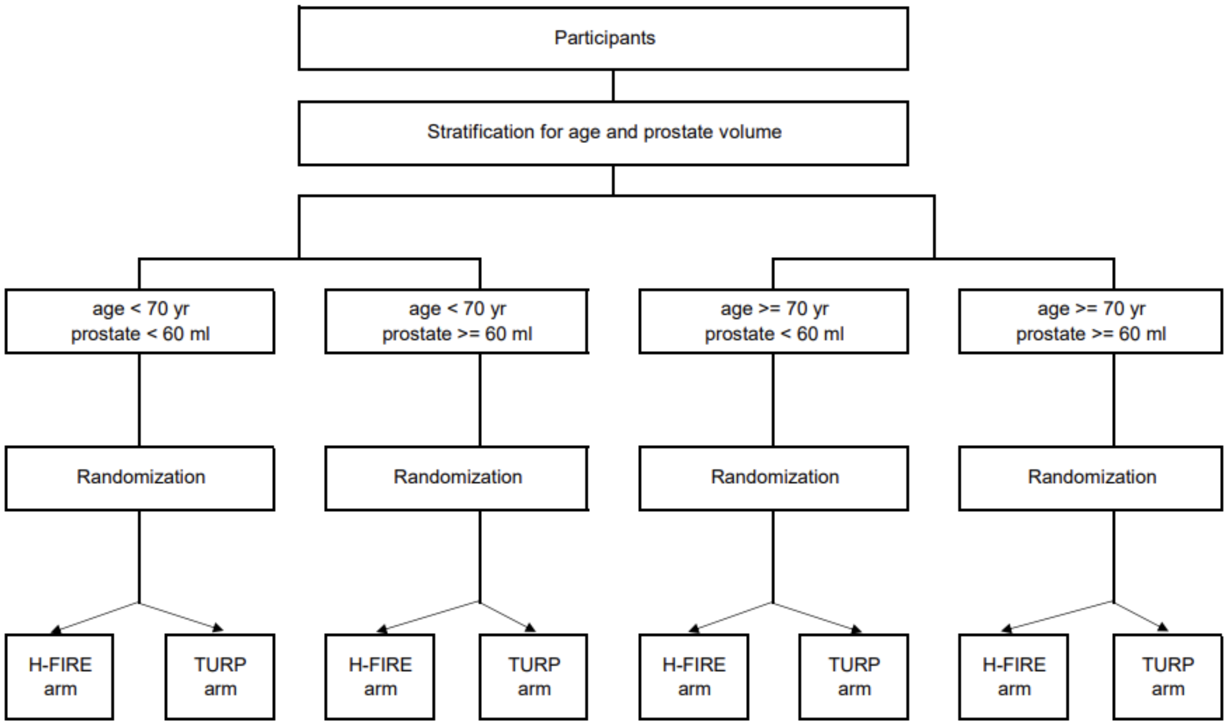

11.2.2. Blinding

To ensure rigorous blinding and minimize bias in outcome assessment, particularly for patient-reported outcomes (PROs), the following measures will be implemented. First, all participants will be blinded to their treatment allocation. To support this, the management protocols for postoperative irrigation and catheter removal will be standardized across both groups. All patients will receive continuous bladder irrigation for a minimum of 24 hours postoperatively, after which the decision to discontinue irrigation will be based solely on urine color. This is done despite irrigation being non-routine for H-FIRE, as it does not impose a significant additional burden and is crucial for maintaining the blind. Similarly, the indwelling urinary catheter will be managed uniformly. The decision for catheter removal prior to discharge will be based on standardized criteria (urine color), irrespective of the assigned procedure. If a patient fails to void after catheter removal, the catheter will be reinserted, and

the patient will be discharged with instructions for removal in the outpatient clinic 1-2 weeks later. Furthermore, during the informed consent process, potential complications will be presented as a combined list from both procedures to prevent patients from deducing their allocation based on their postoperative experience. Finally, as the surgical approaches differ (transperineal for H-FIRE vs. transurethral for TURP), all patients will have a sterile dressing applied to the perineum postoperatively—even though it is not routinely required for both H-FIRE and TURP—to conceal any potential needle puncture sites from H-FIRE. Patients will be instructed to remove the dressing after three days.

The surgical team will not participate in any subsequent patient assessments, data collection, or analysis. Conversely, all researchers responsible for outcome assessment, data collection, and statistical analysis will remain blinded to the treatment allocation throughout the trial. The blind will be broken only after the database is locked and the primary statistical analysis is complete, unless clinical urgency requires knowledge of the treatment for patient safety.

11.3. Interventions

11.3.1 Baseline characteristics

Blood and urine samples will be collected before H-FIRE for a routine examination. The questionnaire (ICIQ score, separate EPIC pad-use item, and IPSS) will be used to assess the baseline urinary symptoms, as well as IIEF-5 and ICIQ--MLUTSsex will be used to evaluate the baseline erectile symptoms. Also, the questionnaire of QOL by IPSS QoL subscore and the HADS will be used to assess the QOL. In addition, the Q_{max} and PVRU will be measured.

Note:

►Urinary symptoms assessment: Patients without bladder catheters will be assessed for urinary symptoms for the past 4 weeks. Patients with bladder catheters will be assessed for urinary symptoms for 4 weeks prior to urinary retention and insertion of the bladder catheter.

►Erectile symptoms assessment: Patients without bladder catheters will be assessed for erectile symptoms for the past 4 weeks. Patients with bladder catheters will be assessed for erectile symptoms for 4 weeks prior to urinary retention and insertion of the bladder catheter.

►QOL assessment: Patients without bladder catheters will be assessed for the QOL for the past 4 weeks. Patients with bladder catheters will be assessed for the IPSS QoL subscore for 4 weeks prior to urinary retention and insertion of the bladder catheter, and the HADS for the past 4 weeks.

11.3.2. Surgery procedure

Both H-FIRE and TURP procedures will be performed by one urologist (HW with experience of more than ten years, with over 200 H-FIRE cases and over 400 TURP cases). This urologist will be blinded to the patient's name， the trial number, and the initial assessment. Also, he will not participate in the subsequent assessment, data collection and statistical analysis.

11.3.2.1 H-FIRE strategy

Participants in this arm will receive H-FIRE using the composite steep pulse therapeutic apparatus manufactured by the REMEDINE company. The procedure will be performed in the lithotomy position under general

anaesthesia with full-muscle paralysis. Under transrectal ultrasound guidance, the electrode needle will be placed on the target lesion via the perineum by getting through a 5 mm brachytherapy template. Four to six needles will be placed around the middle lobe of the prostate, and the distance between two adjacent needles will vary between 0.5-2.0cm, depending on the size of the prostate. The diagram of electrode needle placement is shown in below. After placing the electrode needle, the apparatus will start to release the pre-set pulses. The entire H-FIRE procedure is expected to last 40-60 minutes. A three-way Foley catheter will be indwelled when the procedure has finished.

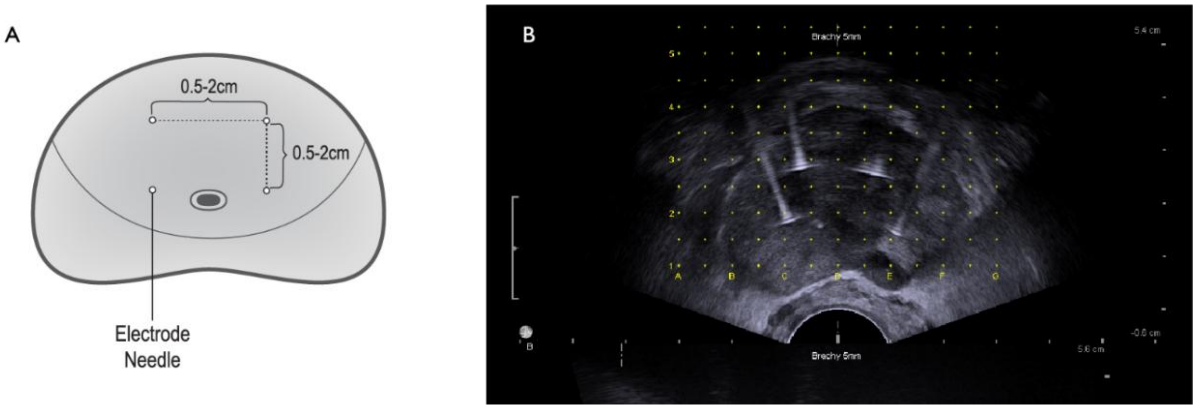

Electrode needle placement

A. Diagram of needle placement; B. Needle placement under transrectal ultrasound guidance

11.3.2.2 TURP strategy

The TURP procedure will be performed with electrodes and a standard tungsten cutting wire loop at 160-W cutting and 80-W coagulating current under general anaesthesia. The resection will begin at the 6 o'clock position of the bladder neck, extending to the verumontanum, and carried down to the surgical capsule, with tissue removal from the gland's centre zone and transition zone. After the procedure, a three-way Foley catheter will be inserted.

11.3.3 After surgery

Irrigation will be applied to all patients, last at least 1 day, and will be stopped based on the urine colour. All patients will receive a dose of antibiotic prophylaxis (cephalosporins or quinolone) on the first day after surgery and routinely last 7 days. The duration of antibiotics may be altered or extended depending on particular circumstances. α1-adrenergic receptor antagonists can be used to relieve the symptoms of bladder spasms and will be abandoned after removing the catheter. Other prostatic drugs can not be used after surgery.

11.4. Follow-up

The primary outcome assessment for the GIANT trial will be conducted at 3 months postoperatively. Regular follow-up visits for the core trial are scheduled at 1 and 3 months.

Long-term Extension Study:
All participants who complete the 3-month visit of the GIANT trial will be invited to enroll in a pre-planned, long-term extension study with a minimum follow-up duration of 24 months. The objective of this extension study is to evaluate the durability of functional outcomes and long-term safety profiles of both H-FIRE and TURP.

Participants who provide additional written informed consent for the extension study will be followed up at 6, 12, and 24 months post-surgery. At these visits, we will systematically collect data on:

►Functional outcomes: Q_{max} and IPSS.

►Long-term safety: Specifically focusing on the incidence of urethral stricture, bladder neck contracture, and surgical re-intervention for BPO.

►Patient-reported outcomes: Quality of life and sexual function.

Beyond the 24-month period, we intend to continue annual follow-up in a registry format, contingent on securing further funding and participant consent, to gather even longer-term data.

11.5. Withdrawal

11.5.1. Reasons for withdrawal

Participants may withdrawal from the trial for the following reasons:

► Participant unwilling to continue the trial

► Investigator judges the withdrawal due to violation of study protocol or poor compliance of the participants

► Investigator deems it in the participant’s best interest to be withdrawn

11.5.2. Handlement for withdrawal

If the participant decides to withdraw from the trial, they will return to the regular clinical pathway and will not continue with scheduled trial follow-ups. The investigator can retain and use the collected data before the withdrawal but discontinue to make further assessments or collect additional data.

12. Analysis plan

Analysis will be performed by statistician who blinded to the treatment allocation. The blinding will remain until the database lock. The details of analysis plan will be included in the comprehensive statistical analysis plan (SAP), which is made prior to the recruiting of the trial. Any change or amendments should be documented and the final version of SAP will be

finalised prior to the database lock. Any analysis not mentioned in the SAP should be reported as secondary analysis. This section is a brief summary of plan for statistics analysis.

12.1. Sample size

We will calculate the sample size based on Q_{max} and IPSS, respectively, and ultimately use the maximum of the two sample sizes.

Literature showed the minimally clinically important differences were 4 mL/s for Q_{max} and 2.5-3 points for IPSS[18 19]

► Sample size calculation based on Q_{max}

For the non-inferiority hypothesis, using 4 mL/s as non-inferiority margin, using 90% power and 2.5% one-sided α, assuming the standard deviation for these two methods are equal as of 6.0 mL/s, using allocation ratio of 1:1, 49 men per arm will be required. Account for 20% withdraw/loss, a total of 118 participants are required for inclusion.

Target Power	Actual Power	α	NIM	D	S1	S2	N1	N2	N
0.8	0.80759	0.025	4	0.0	6	6.0	37	37	74
0.9	0.90434	0.025	4	0.0	6	6.0	49	49	98

The sample size (Q_{max}) in different power (90% or 80%) setting

	Sample Size			Dropout-Inflated Enrollment Sample Size			Expected Number of Dropouts		
Dropout rate	N1	N2	N	N1'	N2'	N'	D1	D2	D
5%	49	49	98	52	52	104	3	3	6
10%	49	49	98	54	54	108	5	5	10
20%	49	49	98	59	59	118	10	10	20

The sample size (Q_{max}) accounting for different dropout rate

► Sample size calculation based on IPSS

For the non-inferiority hypothesis, using 3 point as non-inferiority margin, using 90% power and 2.5% one-sided α, assuming the standard deviation for these two methods are equal as of 4.5 point, using allocation ratio of 1:1, 49 men per arm will be required. Account for 20% withdraw/loss, a total of 118 participants are required for inclusion.

Target Power	Actual Power	α	NIM	D	S1	S2	N1	N2	N
0.8	0.80759	0.025	3	0.0	4.5	4.5	37	37	74
0.9	0.90434	0.025	3	0.0	4.5	4.5	49	49	98

The sample size (IPSS) in different power (90% or 80%) setting

	Sample Size			Dropout-Inflated Enrollment Sample Size			Expected Number of Dropouts		
Dropout rate	N1	N2	N	N1'	N2'	N'	D1	D2	D
5%	49	49	98	52	52	104	3	3	6
10%	49	49	98	54	54	108	5	5	10
20%	49	49	98	59	59	118	10	10	20

The sample size (IPSS) accounting for different dropout rate

The final sample size was calculated to be 118.

12.2. Statistical analysis

12.2.1. Primary outcome

The primary outcome in this trial will be analysed following the intention-to-treat principle as well as the per-protocol principle. The difference between the two groups will be evaluated with a 95% confidence interval (CI) by using the generalised linear mix model. The H-FIRE will be described as non-inferior if the lower bound of the 95%CI of the difference in the Q_{max} and IPSS of the H-FIRE arm compared with the TURP arm (H-FIRE arm minus TURP arm) is higher than −4 mL/s and 3 points(non-inferiority margin).The H-FIRE will be declared noninferior to TURP only if the lower bound of the 95% CI for both Qmax and IPSS is above their respective noninferiority margins (-4 mL/s and -3 points).

12.2.2. Secondary outcomes

The secondary outcomes will be analysed with t-test, chi-square test, Mann Whitney U-test as appropriate and expressed with 95% CIs. Each P value reported in this trial will be two-sided. Further details of the analysis for the secondary outcomes will be provided in the SAP.

12.2.3. Pre-specified subgroup analysis

The pre-specified analysis will be performed with sub-groups where there may be a difference due to population characteristics. Appropriate statistical method will be conducted for subgroup analysis which is provided in the SAP. The subgroup is shown as follows:

►Age: <70 years versus >=70 years

►BMI: <24 versus >=24

►Prostate volume:<60ml versus >=60ml

►Catheterisation status (before surgery): No catheter versus with catheter

►Bladder stone status (before surgery): No bladder stone versus with bladder stone

►Medical treatment of LUTS (before surgery): No drug treatment versus 5α-reductase inhibitors versus α1-adrenergic receptor antagonists versus combination of both drugs mentioned above

12.2.4. Safety analysis

All subjects who undergo surgery (H-FIRE or TURP) will be used for the safety analysis in the trial. Safety will be evaluated by all relevant parameters including adverse experiences, vital signs and laboratory/clinical tests. Details on the approach to analysis safety data are provided in SAP.

13. Harms and adverse events

13.1. Expected harms and adverse events

All harm relevant or not relevant to the treatment will be recorded according to the modified Clavien classification system and the Common Terminology Criteria for Adverse Events (CTCAE) (Appendix 9). The assessment will be made by researchers blinded to the intervention group from the beginning of the trial to 24 months after the surgical procedure. The main expected harms or side effects are listed as below, and the participants will be informed of the risk for these harms or side effects before registration.

Harms or side effects that may occur in this trial (surgery related side effects)

Side effect	Expected probability	Outcome
Hematuria	9 in 10 men	Irrigation or self-resolving, 3-14 days
Hematuria requiring haemoglobin measure	1 in 30 men	Irrigation or self-resolving, 3-14 days
Hematuria requiring transfusion	None	Transfusion
Infection of urine	7 in 10 men	Intravenous or oral antibiotics, 3-7days
Fever	1 in 50 men	Intravenous antibiotics, 3-7days
Clot retention	1 in 20 men	Irrigation, 3-7 days
Prostatic tissue retention	1 in 10 men	Indwelling cathete, 14-28 days
Urinary retention	1 in 10 men	Indwelling cathete, 7-14 days
Urethral stricture	1 in 20 men	Urethral dilatation, 14-28 days
Urinary incontinence	1 in 20 men	Pad used, 7-84 days

Erectile dysfunction	1 in 20 men	Drug used, 7-84 days
Hematospermia	1 in 20 men	Self-resolving, 7-84 days
Lower urinary tract symptoms	1 in 5 men	Self-resolving, 7-28 days
Discomfort when passing urine	2 in 3 men	Self-resolving, 7-14 days
Intra-perineal discomfort	1 in 3 men	Self-resolving, 7-14 days

13.2. Serious adverse events

The serious adverse events are defined as any of the following:

►Death

►Life-threatening

►Need to hospitalization or prolonging an existing hospitalization

►Results in disability or permanent damage

►Congenital deformation or defect

SAEs will be recorded immediately and then sent to the ethics committee and the GIANT monitoring board within 24 hours. With the occurrence of any adverse events or SAEs, necessary therapy will be taken in time to ensure the safety of the participants.

14. Data collection

All data will be collected and be entered into a particular database within 48 hours of each study visit.

14.1. Case Report Forms

The collected clinical data will be recorded by the case report forms (CRFs) for each enrolled participant. The CRFs should be completed with a black or dark blue pen. Any alterations or correction on the CRFs need to be signed with the initials and the date at the bottom of the change. All member involved in data collected for GIANT (e.g. investigator and research nurse) will be training to ensure be familiar with the procedure associated with the CRFs filling.

14.2. Data to be collected on Screening and Inclusion

►Basic data collection: name of patient identification, age (year), PSA (ng/ml), prostate volume (ml), Q_{max} (ml/s), IPSS (scale) and DRE result (normal/abnormal).

14.3. Data to be collected during hospitalization

Data for the participants after the biopsy procedure will be collected as follows:

► Basic data collection (before H-FIRE or TURP): BMI, IIEF-5 (scale), ICIQ—MLUTSsex (scale), PVRU (ml), voided volume (ml), ICIQ (scale), EPIC pad-use item (scale), QOL (scale), HADS (scale), and pain (visual analogue scale), haemoglobin (g/L), serum sodium (mmol/L), and co-medication

► Data collection about surgical procedure (H-FIRE or TURP): Procedure time(minute), weight of resected tissue (g, for TURP)

► Data collection after surgical procedure: Irrigation time (day), bladder catheter indwelling time (day, if the catheter had removed during hospitalization), length of hospital stay (day), transfusion (required /no required), haemoglobin (within 6 hour after surgery, and 24 hour after surgery), serum sodium (within 6 hour after surgery, and 24 hour after surgery), pain (visual analogue scale)

14.4. Data to be collected during follow up

► Data collection at 1 weak: Pain (visual analogue scale)

► Data collection at 1 month: Q_{max} (ml/s), IPSS (scale), IIEF-5 (scale), ICIQ—MLUTSsex (scale), PVRU (ml), voided volume (ml), ICIQ (scale), EPIC pad-use item (scale), QOL (scale), HADS (scale), pain (visual analogue scale), and bladder catheter indwelling time (day, if the catheter had removed after hospitalization)

► Data collection at 3 months: Q_{max} (ml/s), IPSS (scale), IIEF-5 (scale), ICIQ—MLUTSsex (scale), PVRU (ml), voided volume (ml),ICIQ (scale), EPIC pad-use item (scale), QOL (scale), HADS (scale), and pain (visual analogue scale)

14.5. Data retention

CRFs and relative clinical documentation should be kept in a safe place (e.g. locked cabinet in the restricted room) and hold for at least 10 years after the lasted publication of the article related to the trial.

15. Monitoring

15.1. Independent team of clinical research associate

An external monitor team of clinical research associates (CRA) will regularly check the data for completeness and quality at least once weekly. They are responsible for being familiar with the trial protocol. The CRA will contact the responsible person demanding the missing data if the CRF needs to be filled out completely and pose queries to the error data, and the validity of the data will only be confirmed if the questions have been resolved. In addition to

monitoring the data quality and authenticity, the CRA team will also monitor the researchers and the participants during the whole trial, mainly including:

►Monitor the informed consent and the enrolment rate.

►Monitor the participants’ retention or withdrawal.

►Monitor the harms and adverse events.

►Monitor the compliance of participants and investigators with the protocol.

15.2. Data Safety Monitoring Board

GIANT will have an independent Data Safety Monitoring Board (DSMB) to oversee trial safety.

The DSMB’s role is as follows

►Monitor the trial protocol and informed consent documents.

►Monitor the trial's progress, such as participant recruitment, retention, or withdrawal.

►Monitor the safety data during the trial (from enrolled to 2 years after the surgery).

►Monitor the compliance of participants and investigators with the protocol.

►Monitor data quality, timeliness and authenticity.

Based on the monitoring, the DSMB can make the recommendations that whether the trail should continue without change, be modified, or be terminated.

16. Quality control

16.1.Surgical Quality Assurance and Control

To ensure that both surgical procedures are performed to a high and

consistent standard, thereby minimizing operator-dependent bias, the following quality assurance processes are implemented:

16.1.1 Procedural Documentation and Assessment of H-FIRE

Standardized Imaging Documentation: To ensure traceability and consistency of the H-FIRE procedure, transrectal ultrasound (TRUS) images will be systematically captured and saved using a stepper device at three critical timepoints:

►Timepoint 1: Before needle insertion (to document baseline anatomy).

►Timepoint 2: After complete needle placement and before initiating therapy (to document the needle configuration and coverage).

►Timepoint 3: Immediately upon completion of the entire treatment protocol (to document immediate post-procedure sonographic changes, such as areas of increased echogenicity).

At each timepoint, transverse and sagittal images of the prostate will be saved at 5-mm intervals.

Assessment of Procedural Consistency: As there are currently no established standards for the optimal ablation volume in H-FIRE for BPH, the quality control focus for this arm will be on procedural consistency and safety. The saved imaging data will be used to assess:

►Needle Placement Accuracy: Whether the distribution of electrode needles conforms to the pre-defined plan based on prostate volume and morphology.

►Safety Monitoring: Evaluating the relationship of the ablation zones to the prostatic capsule, urethra, and critical surrounding structures (e.g., anterior rectal wall) to ensure the absence of unintended tissue injury.

►Data Archiving: All imaging data will be securely archived to serve as a valuable resource for informing future H-FIRE treatment standards.

16.1.2 Quality Assessment of TURP

Intraoperative Recording and Blinded Expert Review: All TURP procedures will be fully video-recorded. To ensure continuous, representative, and unbiased assessment, the selection of videos for quality control will be performed by an independent statistician who is blinded to patient identity and treatment outcomes.

Sampling Procedure:

►Video Library: All complete TURP videos will be stored in a central repository accessible only to the trial data manager, indexed by consecutive surgery date.

►Block Random Sampling: The independent statistician will use a computer-generated randomization sequence (SAS v9.4, Cary, NC) to select videos. The sampling unit will be consecutive blocks of procedures: for every block of 15 TURP procedures performed in chronological order, the statistician will randomly select 3 complete videos for review.

►Anonymization and Distribution: The trial data manager will then retrieve the specific, randomly selected full-length videos, ensure they are fully anonymized (with all patient identifiers and timestamps removed), and forward them to the three independent urological reviewers.

This process ensures that the selection of cases for review is entirely free from investigator influence, thereby guaranteeing the objectivity of the quality control assessment.

These anonymized video will be reviewed by three independent urological surgeons blinded to the study. Assessment will be conducted via a standardized electronic questionnaire, which includes, but is not limited to:

►Patency of the prostatic urethra post-resection (1-5 Likert scale)

►Adequacy of hemostasis (1-5 Likert scale)

►Presence of residual tissue or obstructive signs at the bladder neck and around the verumontanum (Yes/No, with comments)

►Overall satisfaction with the resection range (e.g., achievement of the surgical capsule, symmetry of resection) (1-5 Likert scale)

Histological Quantification: All resected prostate tissue from TURP procedures will be weighed (in grams) by the operating room nurse prior to fixation. The weight will be recorded as a key perioperative parameter for the objective assessment of tissue removal.

16.2.Quality Control of Randomization Procedures

Randomization Concealment Audit: Upon trial completion and prior to database lock, the DSMB will audit the randomization process. The audit will verify that the generation of all randomization sequences is fully documented with timestamps preceding patient enrollment, and confirm that the allocation sequence for each enrolled participant was revealed only after eligibility was confirmed, ensuring that allocation concealment was not compromised.

16.3.Standardization of Patient Assessment and Data Collection

Standard Operating Procedure for Urodynamic Assessments:

►Q_{max} and PVRU: Strict standard operating procedures for uroflowmetry will be established and adhered to. This will include criteria such as requiring patients to void only when they feel a "strong desire to void" and specifying a minimum interval between repeated measurements. Ultrasound technicians

performing PVRU measurements will receive centralized training and will be required to document transverse and sagittal bladder views in their reports for potential audit.

►PRO Questionnaire Administration: All PRO questionnaires will be administered in a quiet, private setting by trained research coordinators. For patients with limited literacy, questionnaires will be read aloud using a standardized, neutral script to avoid leading questions. All completed questionnaires will be immediately scanned or directly entered into an electronic data capture system, with the original documents stored securely.

16.4.Data Management and Integrity Control

Source Data Verification: Clinical research associates will perform regular source data verification. This involves cross-checking key data points in the electronic database (e.g., primary outcomes Q_{max} and IPSS, serious adverse events) against the source data in the hospital information system (e.g., urodynamic reports, electronic medical records) to ensure accurate transcription. Critical data points requiring 100% verification will be explicitly defined in the data management plan.

16.5.Endpoint Adjudication Committee

This is a core recommendation to enhance the objectivity of outcome assessment.

Function: An independent Endpoint Adjudication Committee (EAC), composed of multiple urology specialists who remain blinded to treatment allocation, will be established.

Adjudication Responsibilities: The EAC will be responsible for the blinded adjudication of predefined key serious adverse events and functional

outcomes. This includes, but is not limited to:

►Events Requiring Blood Transfusion: Adjudicating whether the event is directly related to the surgical procedure.

►Urinary Incontinence: Determining, based on EPIC pad-use and ICIQ scores, whether the condition meets the predefined criteria for "clinically significant urinary incontinence."

►Erectile Dysfunction: Adjudicating whether a change in IIEF-5 score constitutes a new onset or significant worsening of erectile function.

►Re-operation Events: Determining if the cause for re-operation is related to failure of or complications from the initial procedure.

►Process: The committee will review all relevant medical records, laboratory reports, and PRO data (with treatment allocation masked) and will classify events according to pre-established charter definitions. This process ensures that the determination of key safety and functional endpoints is objective and consistent.

17. Ethics and dissemination

Ethical approval for the GIANT trial, including its long-term extension study, was obtained from the institutional ethics committee of Shanghai East Hospital. All participants provided written informed consent for the initial 3-month study, and separate consent will be obtained for participation in the long-term extension phase.

The primary manuscript from the GIANT trial will report the 3-month efficacy and safety outcomes. In this publication, we will transparently acknowledge that the assessment of long-term durability and certain safety events (e.g., urethral stricture) is limited by the 3-month follow-up period. We will explicitly state that these long-term outcomes are being systematically evaluated in the

ongoing extension study, the results of which will be presented in future publications.

18. Authors' contributions

The study protocol was conceived and designed by Bi-Ming He and Hai-Feng Wang. The original draft was completed by Bi-Ming He and revised by Hai-Feng Wang.

19. Declaration of interests

None of the authors have any conflicts of interest to declare.

20. Protocol Amendments

20.1. Original protocol (V1.0) (March-2022)

Amendments: /

20.2. Protocol V2.0 (April-2022)

Amendments:

Major amendments:

1.Changes to the description of study design.

►Change "GIANT is a prospective, investigator-initiated, single-centre, randomised controlled, single-blinded and non-inferiority study with blinded outcome assessment in which men with lower urinary tract symptoms and

benign prostatic obstruction” into “GIANT is a prospective, investigator-initiated, single-centre, randomised controlled, double-blinded and non-inferiority study in which men with lower urinary tract symptoms and benign prostatic obstruction”. (section PROTOCOL SUMMARY)

2.Changes to the description of primary outcome.

►Change “The primary outcome is to assess the change from baseline in maximal flow rate (Q_{max}) at 1, 3, 6, 12 and 24 months after surgical treatment.” into “The co-primary outcome is to assess the change from baseline in maximal flow rate (Q_{max}) and urinary symptoms by questionnaire of International Prostate Symptom Score (IPSS) scoring at 3 months after surgical treatment”.(section PROTOCOL SUMMARY)

3.Changes to the description of secondary outcomes.

►Change “1. To assess the improvement from baseline in urinary symptoms by questionnaire of International Prostate Symptom Score (IPSS) scoring at 1, 3, 6, 12 and 24 months after surgical treatment. 2. To assess the improvement from baseline in erectile symptoms by the 5-item version of the International Index of Erectile Function (IIEF-5) scoring and the International Consultation on Incontinence Questionnaire Male Sexual Matters Associated with Lower Urinary Tract Symptoms Module (ICIQ-MLUTSsex) at 1, 3, 6, 12 and 24 months after surgical treatment. 3.To assess the post-void residual urine volume (PVRU) at 1, 3, 6, 12 and 24 months after surgical treatment and the change from baseline. 4.To assess the voided volume at 1, 3, 6, 12 and 24 months after surgical treatment and the change from baseline. 5.To assess the urinary incontinence by ICIQ (International Consultation on Incontinence Questionnaire) score and separate EPIC (Expanded Prostate Cancer Index Composite) pad-use item at 1, 3, 6, 12 and 24 months after surgical treatment.

6.To assess the quality of life (QOL) by IPSS QoL subscore[1] and Hospital Anxiety and Depression Scale (HADS) at 1, 3, 7, 12 and 24 months after surgical treatment and the change from baseline.8.To assess the perioperative parameters, including operative time, the postoperative hospital stay, haemoglobin declination, serum sodium declination, and catheterisation duration.9.To assess the early postoperative urinary symptoms include dysuria, urgency, or postmicturition pain.10.To assess the pain at 24 hours, 1 week, 1 month and 3 months.11.To assess the adverse event at every visit. It mainly includes transurethral resection syndrome, blood transfusion, clot retention, urinary tract infection (UTI), fever and other adverse events according to the Common Terminology Criteria for Adverse Events (CTCAE). ” into “1. To assess the improvement from baseline in erectile symptoms by the 5-item version of the International Index of Erectile Function (IIEF-5) scoring and the International Consultation on Incontinence Questionnaire Male Sexual Matters Associated with Lower Urinary Tract Symptoms Module (ICIQ-MLUTSsex) at 3 months after surgical treatment. 2. To assess the change from baseline in post-void residual urine volume (PVRU) at 3 months after surgical treatment.
3. To assess the change from baseline in voided volume at 3 months after surgical treatment.4.To assess the change from baseline in the urinary incontinence by ICIQ (International Consultation on Incontinence Questionnaire) score and separate EPIC (Expanded Prostate Cancer Index Composite) pad-use item at 3 months after surgical treatment.5.To assess the change from baseline in the quality of life (QOL) by IPSS QoL subscore[1] and Hospital Anxiety and Depression Scale (HADS) at 3 months after surgical treatment. 6.To assess the perioperative parameters, including operative time, the postoperative hospital stay, haemoglobin declination, serum sodium declination, and catheterisation duration.7.To assess the early postoperative urinary symptoms include dysuria, urgency, or postmicturition pain.8.To assess the change from baseline in the pain at 3 months after surgical treatment.9.To

assess the adverse event at every visit. It mainly includes transurethral resection syndrome, blood transfusion, clot retention, urinary tract infection (UTI), fever and other adverse events according to the Common Terminology Criteria for Adverse Events (CTCAE)".(section PROTOCOL SUMMARY)

4.Changes to the description of analysis.

►Change "The primary outcome in this trial will be analysed following the intention-to-treat principle as well as the per-protocol principle. The difference between the two groups will be evaluated with a 95% confidence interval (CI) by using the generalised linear mix model. The H-FIRE will be described as non-inferior if the lower bound of the 95%CI of the difference in the Q_{max} of the H-FIRE arm compared with the TURP arm (H-FIRE arm minus TURP arm) is higher than −4mL/s (non-inferiority margin). The second outcome will be appropriately analysed for the different distribution (e.g., t-test, Pearson chi-square test, etc.) and described with 95%CI. Each reported P value in this trial will be two-sided. " into "The primary outcome in this trial will be analysed following the intention-to-treat principle as well as the per-protocol principle. The difference between the two groups will be evaluated with a 95% confidence interval (CI) by using the generalised linear mix model. The H-FIRE will be described as non-inferior if the lower bound of the 95%CI of the difference in the Q_{max} and IPSS of the H-FIRE arm compared with the TURP arm (H-FIRE arm minus TURP arm) is higher than −4mL/s and 3 points(non-inferiority margin). The second outcome will be appropriately analysed for the different distribution (e.g., t-test, Pearson chi-square test, etc.) and described with 95%CI. Each reported P value in this trial will be two-sided. " (section PROTOCOL SUMMARY)

5.Changes to the trial design.

►Change “The primary objective of this study is to determine whether the H-FIRE is non-inferior to the TURP for achieving better functional outcomes in the short, middle, and long term.” Into “The primary objective of this study is to determine whether the H-FIRE is non-inferior to the TURP for achieving better functional outcomes.”(section 9.1 Trial overview).

►Change the design overview chart (section 9.1 Trial overview).

6.Changes to the timeframe

►Change to the table1 (section 9.4 Timeframe)

7.Changes to the outcomes

►Change “The primary outcome is to assess the change from baseline in maximal flow rate (Q_{max}) at 1, 3, 6, 12 and 24 months after surgical treatment.” into “The co-primary outcome is to assess the change from baseline in maximal flow rate (Q_{max}) urinary symptoms by questionnaire of International Prostate Symptom Score (IPSS)[1] scoring at 3 months after surgical treatment.” (section 10.1 primary outcome)

►Addition of “IPSS is a well-established and validated patient-reported outcome, with 7 items to assess the degree of urinary symptoms during the last 4 weeks, ranging from 0 to 35, with higher scores indicating more severe urinary symptoms (Appendix 1). IPSS will be assessed for those who have removed the bladder catheter after the surgery. If patients still have bladder catheters, IPSS will not be assessed.” into the definition of primary outcome (section 10.1 primary outcome).

► Deletion of “To assess the improvement from baseline in urinary symptoms by questionnaire of International Prostate Symptom Score (IPSS)[1] scoring at 1, 3, 6, 12 and 24 months after surgical treatment.” and the following definition (section 10.2 secondary outcomes).

►Change "To assess the improvement from baseline in erectile symptoms by the 5-item version of the International Index of Erectile Function (IIEF-5)[12] scoring and the International Consultation on Incontinence Questionnaire Male Sexual Matters Associated with Lower Urinary Tract Symptoms Module (ICIQ--MLUTSsex)[13] at 1, 3, 6, 12 and 24 months after surgical treatment." into "To assess the improvement from baseline in erectile symptoms by the 5-item version of the International Index of Erectile Function (IIEF-5)[12] scoring and the International Consultation on Incontinence Questionnaire Male Sexual Matters Associated with Lower Urinary Tract Symptoms Module (ICIQ-MLUTSsex)[13] at 3 months after surgical treatment."(section 10.2 secondary outcomes).

►Change "To assess the post-void residual urine volume (PVRU) at 1, 3, 6, 12 and 24 months after surgical treatment and the change from baseline." into "To assess the change from baseline in the post-void residual urine volume (PVRU) at 3 months after surgical treatment." (section 10.2 secondary outcomes).

►Change "To assess the voided volume at 1, 3, 6, 12 and 24 months after surgical treatment and the change from baseline." into "To assess the change from baseline in the voided volume at 3 months after surgical treatment." (section 10.2 secondary outcomes).

►Change "To assess the urinary incontinence by ICIQ (International Consultation on Incontinence Questionnaire)[14] score and separate EPIC (Expanded Prostate Cancer Index Composite)[15] pad-use item at 1, 3, 6, 12 and 24 months after surgical treatment." into "To assess the change from baseline in the urinary incontinence by ICIQ (International Consultation on Incontinence Questionnaire)[14] score and separate EPIC (Expanded Prostate Cancer Index Composite)[15] pad-use item at 3 months after surgical treatment." (section 10.2 secondary outcomes).

►Change "To assess the quality of life (QOL) by IPSS QoL subscore[1] and Hospital Anxiety and Depression Scale (HADS)[16] at 1, 3, 6, 12 and 24 months after surgical treatment and the change from baseline." into "To assess the

change from baseline in the quality of life (QOL) by IPSS QoL subscore[1] and Hospital Anxiety and Depression Scale (HADS)[16] at 3 months after surgical treatment.” (section 10.2 secondary outcomes).

►Change “To assess the pain[17] at 24 hours, 1 week, 1 month and 3 months.” into “To assess the change from baseline in the pain[17] at 3 months after surgical treatment.” (section 10.2 secondary outcomes).

8.Changes to follow-up

►Change “Regular follow-up will begin one month after the surgical procedure and continue up to 24 months. ” into “The primary outcome assessment for the GIANT trial will be conducted at 3 months postoperatively. Regular follow-up visits for the core trial are scheduled at 1 and 3 months.” (section 11.4 follow-up)

►Added “Long-term Extension Study” section (section 11.4 follow-up)

9.Changes to sample size

►Rewrite the section 12.1 sample size

10.Changes to statistical analysis

►Change “The primary outcome in this trial will be analysed following the intention-to-treat principle as well as the per-protocol principle. The difference between the two groups will be evaluated with a 95% confidence interval (CI) by using the generalised linear mix model. The H-FIRE will be described as non-inferior if the lower bound of the 95%CI of the difference in the Q_{max} of the H-FIRE arm compared with the TURP arm (H-FIRE arm minus TURP arm) is higher than −4 mL/s (non-inferiority margin).” into “The primary outcome in this trial will be analysed following the intention-to-treat principle as well as the per-protocol principle. The difference between the two groups will be evaluated with a 95% confidence interval (CI) by using the generalised linear mix model.

The H-FIRE will be declared noninferior to TURP only if the lower bound of the 95% CI for both Qmax and IPSS is above their respective noninferiority margins (-4 mL/s and -3 points).” (section 12.2.1 primary outcome).

11. Deletion of interim analysis

► Deletion of “A blinded interim analysis will be performed after 70 men (60% of the targeted sample size) have a 3-months follow-up. The analysis will examine whether the difference in Q_{max} improvement between THE H-FIRE arm and the TURP arm is not greater than 20%. ”

12.Change in data collection

►Deletion of “► Data collection at 6 months: Q_{max} (ml/s), IPSS (scale), IIEF-5 (scale), ICIQ—MLUTSsex (scale), PVRU (ml), voided volume (ml),ICIQ (scale), EPIC pad-use item (scale), QOL (scale), and HADS (scale)

► Data collection at 12 months: Q_{max} (ml/s), IPSS (scale), IIEF-5 (scale), ICIQ—MLUTSsex (scale), PVRU (ml), voided volume (ml),ICIQ (scale), EPIC pad-use item (scale), QOL (scale), HADS (scale), and PSA (ng/ml)

► Data collection at 24 months: Q_{max} (ml/s), IPSS (scale), IIEF-5 (scale), ICIQ—MLUTSsex (scale), PVRU (ml), voided volume (ml), ICIQ (scale), EPIC pad-use item (scale), QOL (scale), HADS (scale), and PSA (ng/ml)”.(section 14.4 data to be collected during follow up).

13. Add quality control as a new section.

►Addition of section “Surgical Quality Assurance and Control”(section 16.1.)

►16.1.1Procedural Documentation and Assessment of H-FIRE(section 16.1.1)

►Addition of section “ Quality Assessment of TURP” (section 16.1.2)

►Addition of section “Quality Control of Randomization Procedures” (section 16.2)

►Addition of section “Standardization of Patient Assessment and Data Collection” (section 16.3)

►Addition of section “Data Management and Integrity Control” (section 16.4)

►Addition of section “Endpoint Adjudication Committee” (section 16.5)

►Addition of appendix 10

14. Addition of section “Co-Investigator and GIANT members” (section 4.3)

Other amendments:

►Change “15.2. Independent team of clinical research associate” into “15.1. Independent team of clinical research associate” (section 15.1. Independent team of clinical research associate)

►Change “ HI-FIRE” into “H-FIRE” (section 7. Abstract).

15. Change in Ethics and dissemination

►Change “Ethical approval was obtained from the ethics committee of Shanghai East Hospital. The results of this trial will be disseminated to an international peer-reviewed journal and disseminated for presentation at an international or national academic conference.” into “Ethical approval for the GIANT trial, including its long-term extension study, was obtained from the institutional ethics committee of Shanghai East Hospital. All participants provided written informed consent for the initial 3-month study, and separate consent will be obtained for participation in the long-term extension phase. The primary manuscript from the GIANT trial will report the 3-month efficacy and safety outcomes. In this publication, we will transparently acknowledge that the assessment of long-term durability and certain safety events (e.g., urethral stricture) is limited by the 3-month follow-up period. We will explicitly state that these long-term outcomes are being systematically evaluated in the

ongoing extension study, the results of which will be presented in future publications.”

20.3. Protocol V3.0 (Jan-2023)

Change in the description of blinding methods

►Change “All participants will be blinded to the treatment allocation to reduce bias in assessing outcomes, especially of the PROs. The researcher responsible for performing the surgical procedure will not go on to participate in the subsequent assessment, data collection and statistical analysis. The researchers responsible for assessment and statistical analysis will be blinded to the treatment allocation. Blinding will only be broken when the statistical analysis has been completed unless the knowledge of the blinded treatment may influence patient care or safety.” into “To ensure rigorous blinding and minimize bias in outcome assessment, particularly for patient-reported outcomes (PROs), the following measures will be implemented. First, all participants will be blinded to their treatment allocation. To support this, the management protocols for postoperative irrigation and catheter removal will be standardized across both groups. All patients will receive continuous bladder irrigation for a minimum of 24 hours postoperatively, after which the decision to discontinue irrigation will be based solely on urine color. This is done despite irrigation being non-routine for H-FIRE, as it does not impose a significant additional burden and is crucial for maintaining the blind. Similarly, the indwelling urinary catheter will be managed uniformly. The decision for catheter removal prior to discharge will be based on standardized criteria (urine color), irrespective of the assigned procedure. If a patient fails to void after catheter removal, the catheter will be reinserted, and the patient will be discharged with instructions for removal in the outpatient clinic 1-2 weeks later. Furthermore, during the informed consent process, potential complications will be presented as a combined list from both procedures to prevent patients from

deducing their allocation based on their postoperative experience. Finally, as the surgical approaches differ (transperineal for H-FIRE vs. transurethral for TURP), all patients will have a sterile dressing applied to the perineum postoperatively—even though it is not routinely required for both H-FIRE and TURP—to conceal any potential needle puncture sites from H-FIRE. Patients will be instructed to remove the dressing after three days.

The surgical team will not participate in any subsequent patient assessments, data collection, or analysis. Conversely, all researchers responsible for outcome assessment, data collection, and statistical analysis will remain blinded to the treatment allocation throughout the trial. The blind will be broken only after the database is locked and the primary statistical analysis is complete, unless clinical urgency requires knowledge of the treatment for patient safety." (section 11.2.2 blinding)

Other amendments:

►Change "4.5 ml/s" into "4.5 point" (section 12.1 Sample size)

20. References

1. MJ B, Jr FF, MP OL, et al. The American Urological Association symptom index for benign prostatic hyperplasia. The Measurement Committee of the American Urological Association. *J Urol* 1992 Nov;148(5):1549-57. doi: 10.1016/j.juro.2016.10.071 [published Online First: 2016/12/26]
2. Berry Sj Fau - Coffey DS, Coffey Ds Fau - Walsh PC, Walsh Pc Fau - Ewing LL, et al. The development of human benign prostatic hyperplasia with age. *J Urol* 1984 Sep;132(3)(0022-5347 (Print)):474-9.
3. Mottet N, Cornford P, Bergh RCNvd, et al. EAU Guidelines-2020. 2020
4. Huang SW, Tsai CY, Tseng CS, et al. Comparative efficacy and safety of new surgical treatments for benign prostatic hyperplasia: systematic review and network meta-analysis. *BMJ* 2019;367:l5919. doi: 10.1136/bmj.l5919 [published Online First: 2019/11/16]
5. Lourenco T, Pickard R Fau - Vale L, Vale L Fau - Grant A, et al. Minimally invasive treatments for benign prostatic enlargement: systematic review of randomised controlled trials. *BMJ* 2008 Oct 9;337:a1662(1756-1833 (Electronic))
6. Reich O, Gratzke C Fau - Bachmann A, Bachmann A Fau - Seitz M, et al. Morbidity, mortality and early outcome of transurethral resection of the prostate: a prospective multicenter evaluation of 10,654 patients. *J Urol* 2008 Jul;180(1)(1527-3792 (Electronic))
7. Davalos RV, Mir IL, Rubinsky B. Tissue ablation with irreversible electroporation. *Ann Biomed Eng* 2005;33(2):223-31. doi: 10.1007/s10439-005-8981-8 [published Online First: 2005/03/18]
8. Li W, Fan Q, Ji Z, et al. The effects of irreversible electroporation (IRE) on nerves. *PLoS One* 2011;6(4):e18831. doi: 10.1371/journal.pone.0018831 [published Online First: 2011/05/03]
9. Tsivian M, Polascik TJ. Bilateral focal ablation of prostate tissue using low-energy direct current (LEDC): a preclinical canine study. *BJU Int* 2013;112(4):526-30. doi: 10.1111/bju.12227 [published Online First: 2013/07/25]
10. Arena CB, Sano Mb Fau - Rossmeisl JH, Jr., Rossmeisl Jh Jr Fau - Caldwell JL, et al. High-frequency irreversible electroporation (H-FIRE) for non-thermal ablation without muscle contraction. *Biomed Eng Online* 2011(1475-925X (Electronic)):10:102.
11. Wang H, Xue W, Yan W, et al. Extended Focal Ablation of Localized Prostate Cancer With High-Frequency Irreversible Electroporation: A Nonrandomized Controlled Trial. *JAMA Surg* 2022 doi: 10.1001/jamasurg.2022.2230 [published Online First: 2022/07/07]
12. RC R, JC C, MD S, et al. Development and evaluation of an abridged, 5-item version of the International Index of Erectile Function (IIEF-5) as a diagnostic tool for erectile dysfunction. *Int J Impot Res* 1999 Dec;11(6):319-26.
13. SJ F, JL D, TI P, et al. Sexual dysfunction in men with lower urinary tract symptoms. *J Clin Epidemiol* 1998 Aug;51(8):677-85.
14. Avery K, Donovan J, Peters TJ, et al. ICIQ: a brief and robust measure for evaluating the symptoms and impact of urinary incontinence. *Neurourol Urodyn* 2004;23(4):322-30. doi: 10.1002/nau.20041 [published Online First: 2004/07/01]
15. JT W, RL D, MS L, et al. Development and validation of the expanded prostate cancer index

composite (EPIC) for comprehensive assessment of health-related quality of life in men with prostate cancer. *Urology* 2000;56(6):899-905.

16. AS Z, RP S. The hospital anxiety and depression scale. *Acta Psychiatr Scand* 1983 Jun;1983 Jun(67(6)):361-70.
17. McCarthy M, Jr., Chang CH, Pickard AS, et al. Visual analog scales for assessing surgical pain. *J Am Coll Surg* 2005;201(2):245-52. doi: 10.1016/j.jamcollsurg.2005.03.034 [published Online First: 2005/07/26]
18. Hashim H, Worthington J, Abrams P, et al. Thulium laser transurethral vaporesection of the prostate versus transurethral resection of the prostate for men with lower urinary tract symptoms or urinary retention (UNBLOCS): a randomised controlled trial. *Lancet* 2020;396(10243):50-61. doi: 10.1016/s0140-6736(20)30537-7
19. Abt D, Hechelhammer L, Müllhaupt G, et al. Comparison of prostatic artery embolisation (PAE) versus transurethral resection of the prostate (TURP) for benign prostatic hyperplasia: randomised, open label, non-inferiority trial. *Bmj* 2018;361:k2338. doi: 10.1136/bmj.k2338 [published Online First: 20180619]

Appendix 1: International Prostate Symptom Score (IPSS)

International prostate symptom score (I-PSS)

Symptoms in the past month	In 5 times						Score
	Not at all	Less than one	Less than half the time	About half the time	More than half the time	Almost always	
1. How often have you had the sensation of incomplete emptying your bladder?							
2. How often have you had to urinate less than every two hours?							
3. How often have you found you stopped and started again several times when you urinated?							
4. How often have you found it difficult to postpone urination?							
5. How often have you had a weak urinary stream?							
6. How often have you had to strain to start urination?							
7. How many times did you typically get up at night to urinate?	None	Once	2 times	3 times	4 times	5 times	
	0	1	2	3	4	5	
						**Overall score=**	

Note: Mild – scores between 0 and 7; Moderate – scores between 8 and 19; Severe – scores between 20 and 35. A postoperative increase in the overall score of 5 is considered adverse effect of the operation on the urinary tract function.

(QOL)

	Delighted	Pleased	Mostly satisfied	Mixed (about	Mostly dissatisfied	Unhappy	Terrible
If you were to spend the rest of your life with your prostate symptoms just as they are now, how would you feel about that?	0	1	2	3	4	5	6
(QOL) =							

signature:................ date:.................

Appendix 2: 5-item version of the International Index of Erectile Function (IIEF-5)

International Index of Erectile Function (IIEF-5):

	0	1	2	3	4	5	Score
1. How do you rate your confidence that you could get and keep an erection?	Not at all	Very low	Low	Moderate	High	Very high	
2. When you had erections with sexual stimulation, how often were your erections hard enough for penetration?	Not at all	Almost never /never	A few times	Some-times (about half the time)	Most times (much more than half the time)	Almost always /always	
3. During sexual intercourse, how often were you able to maintain your erection after you had penetrated (entered) your partner?	Not at all	Almost never /never	A few times	Some-times (about half the time)	Most times (much more than half the time)	Almost always /always	
4. During sexual intercourse, how difficult was it to maintain your erection to completion of intercourse?	Not at all	Extremely difficult	Very difficult	Difficult	Slightly difficult	Not difficult	
5. When you attempted sexual intercourse, how often was it satisfactory for you?	Not at all	Almost never /never	A few times	Some-times (about half the time)	Most times (much more than half the time)	Almost always /always	

Note: Patients encircle the response that best describes for the five questions over the past 6 months. The IIEF-5 score is the sum of the ordinal responses to the 5 items. Scores >21: Normal erectile function; Scores 12-21: Mild erectile dysfunction; Scores 8-11: Moderate erectile dysfunction; Scores 1-7: Severe erectile dysfunction.

signature: .... date: ....

Appendix 3: International Consultation on Incontinence Questionnaire Male Sexual Matters Associated with Lower Urinary Tract Symptoms Module (ICIQ-MLUTSsex)

1. To what extent do you feel that your sex life has been spoilt by your urinary symptoms?

- ☐ not at all
- ☐ a little
- ☐ somewhat
- ☐ alot

How much of a problem is this for you?

- ☐ not a problem
- ☐ a bit of a problem
- ☐ quite a problem
- ☐ a serious problem

2. Do you get erections?

- ☐ yes, with normal rigidity
- ☐ yes, with reduced rigidity
- ☐ yes, with severely reduced rigidity
- ☐ no, erection not possible

How much of a problem is this for you?

- ☐ not a problem
- ☐ a bit of a problem
- ☐ quite a problem
- ☐ a serious problem

3. Do you have an ejaculation of semen?

- ☐ yes, normal quantity
- ☐ yes, reduced quantity
- ☐ yes, significantly reduced quantity
- ☐ no ejaculation

How much of a problem is this for you?

- ☐ not a problem
- ☐ a bit of a problem
- ☐ quite a problem
- ☐ a serious problem

4. Do you have pain or discomfort during ejaculation?

- ☐ no
- ☐ yes, slight pain/discomfort
- ☐ yes, moderate pain/discomfort
- ☐ yes, severe pain/discomfort

How much of a problem is this for you?

- ☐ not a problem
- ☐ a bit of a problem
- ☐ quite a problem
- ☐ a serious problem

signature: .... date: ....

Appendix 4: International Consultation on Incontinence Questionnaire (ICIQ)

[ICIQ]

signature:.................... date:....................

Many people leak urine some of the time. We are trying to find out how many people leak urine, and how much this bothers them. We would be grateful if you could answer the following questions, thinking about how you have been, on average, over the PAST FOUR WEEKS.

1.How often do you leak urine?

Never	☐	0
About once a week or less often	☐	1
Two or three times a week	☐	2
About once a day	☐	3
Several times a day	☐	4
All the time	☐	5

We would like to know how much you think leaks.

2.How much urine do you usually leak (whether you wear protection or not)?

None	☐	0
A small amount	☐	2
A moderate amount	☐	4
A large amount	☐	6

3.Overall, how much does leaking urine interfere with your everyday life?

Please ring a number between 0 (not at all) and 10 (a great deal)

0 1 2 3 4 5 6 7 8 9 10

not at all ... a great deal

ICIQ score: sum scores 1+2+3....................

Appendix 5: Expanded Prostate Cancer Index Composite (EPIC) pad-use item

pad usage

How many pads or adult diapers per day did you usually use to control leakage during the last 4 weeks?

- no pads ☐
- 1 pad per day ☐
- 2 pads per day ☐
- 3 or more pads per day ☐

signature: .... date: ....

Appendix 6: Hospital Anxiety and Depression Scale (HADS)

【HADS】

沿虚线折叠

Doctors are aware that emotions play an important part in most illnesses. If your doctor knows about these feelings he will be able to help you more.
This questionnaire is designed to help your doctor to know how you feel. Ignore the numbers printed on the left of the questionnaire. Read each item and underline the reply which comes closest to how you have been feeling in the past week.
Don't take too long over your replies; your immediate reaction to each item will probably be more accurate than a long thought out response.

A

1. I feel tense or 'wound up':

- 3 ☐ Most of the time
- 2 ☐ A lot of the time
- 1 ☐ From time to time, occasionally
- 0 ☐ Not at all

D

2. I still enjoy the things I used to enjoy:

- 0 ☐ Definitely as much
- 1 ☐ Not quite so much
- 2 ☐ Only a little
- 3 ☐ Hardly at all

A

3. I get a sort of frightened feeling as if something awful is about to happen:

- 3 ☐ Very definitely and quite badly
- 2 ☐ Yes, but not very badly
- 1 ☐ A little, but it doesn't worry me
- 0 ☐ Not at all

D

4. I can laugh and see the funny side of things:

- 0 ☐ As much as I always could
- 1 ☐ Not quite so much now
- 2 ☐ Definitely not so much now
- 3 ☐ Not at all

A

5. Worrying thoughts go through my mind:

- 3 ☐ A great deal of time
- 2 ☐ A lot of time
- 1 ☐ From time to time but not too often
- 0 ☐ Only occasionally

D

6. I feel cheerful:

- 3 ☐ Not at all
- 2 ☐ Not often
- 1 ☐ Sometimes
- 0 ☐ Most of the time

A

7. I can sit at ease and feel relaxed:

- 0 ☐ Definitely
- 1 ☐ Usually
- 2 ☐ Not often
- 3 ☐ Not at all

D

8. I feel as if I am slowed down:

- 3 ☐ Nearly all the time
- 2 ☐ Very often
- 1 ☐ Sometimes
- 0 ☐ Not at all

A

9. I get a sort of frightened feeling like 'butterflies' in the stomach:

- 0 ☐ Not at all
- 1 ☐ Occasionally
- 2 ☐ Quiet often
- 3 ☐ Very often

D

10. I have lost interest in my appearance:

- 3 ☐ Definitely
- 2 ☐ I don't take so much care as I should
- 1 ☐ I may not take quite as much care
- 0 ☐ I take just as much care as ever

A

11. I feel restless as if I have to be on the move:

- 3 ☐ Very much indeed
- 2 ☐ Quite a lot
- 1 ☐ Not very much
- 0 ☐ Not at all

D

12. I look forward with enjoyment to things:

- 0 ☐ As much as ever I did
- 1 ☐ Rather less than I used to
- 2 ☐ Definitely less than I used to
- 3 ☐ Hardly at all

A

13. I get sudden feelings of panic:

- 3 ☐ Very often indeed
- 2 ☐ Quite often
- 1 ☐ Not very often
- 0 ☐ Not at all

D

14. I can enjoy a good book or radio or TV program:

- 0 ☐ Often
- 1 ☐ Sometimes
- 2 ☐ Not often
- 3 ☐ Very seldom

signature: .... date: ....

▶ **For hospital use only**

D (8-10)
A (8-10)

Appendix 7: Early postoperative urinary symptoms assessment

Early postoperative urinary symptoms assessment	
Blood in the urine	not at all ☐<br>minor ☐<br>moderate ☐<br>severe ☐
Difficulty in urination or urinary retention	not at all ☐<br>minor ☐<br>moderate ☐<br>severe ☐
painful during passing urine	not at all ☐<br>minor ☐<br>moderate ☐<br>severe ☐
painful after passing urine	not at all ☐<br>minor ☐<br>moderate ☐<br>severe ☐
urine urgency	not at all ☐<br>minor ☐<br>moderate ☐<br>severe ☐

signature: .... date: ....

Appendix 8: Surgical Pain Scale

SURGICAL PAIN SCALES

1. What was the average amount of pain you had when you were **at rest?**

0 1 2 3 4 5 6 7 8 9 10

【No pain sensation】 【Most intense pain imaginable】

2. How much pain did you have during your **normal activities**?
(for example, walking, climbing stairs, driving a car, getting up from a chair)

0 1 2 3 4 5 6 7 8 9 10

【No pain sensation】 【Most intense pain imaginable】

3. How much pain did you have when you were having **sex, exercising, doing strenuous work,** or **lifting** objects you used to be able to lift comfortably?

☐ I have not done any of these activities today

0 1 2 3 4 5 6 7 8 9 10

【No pain sensation】 【Most intense pain imaginable】

4. How **unpleasant or disturbing** was the **worst pain** that you had today?

0 1 2 3 4 5 6 7 8 9 10

【No bad at all】 【Most intense bad feeling imaginable】

signature: .... **date :** ....

Appendix 9 Common Terminology Criteria for Adverse Events (CTCAE)

Common Terminology Criteria for Adverse Events (CTCAE) v5.0

Grade	Definition
Grade 1	Mild;asymptomatic or mild symptoms; clinical or diagnostic observations only; intervention not indicatied
Grade 2	Moderate;minimal, local or noninvasive intervention indicated; limiting age-approproate instrumental ADL*.
Grade 3	Severe or medically significant but not immediately life-threatening; hospitalization or prolongation of hospitalization in indicated; disabling; limiting self care ADL**.
Grade 4	Life-threatening consequences; urgent intervention indicated.
Grade 5	Death related to AE

*Instrumental ADL refer to preparing meals, shopping for groceries or clothes, using the telephone, managing money,ect.
**Self care ADL refer to bathing, dressing and undressing, feeding self, using the toilet, taking medications, and not bedridden.

Appendix 10 TURP Technical Quality Assessment Questionnaire

TURP Technical Quality Assessment Questionnaire

Instructions for Independent Assessors:

Dear Professor,

Thank you for serving as an independent assessor for the GIANT trial. You will be reviewing anonymized video of TURP procedures. Please evaluate the technical quality based on the following criteria. Your blinded assessment is crucial for ensuring the quality and standardization of the surgical interventions in this study.

Video ID: _______________

Reviewer ID: _______________

Date of Review: _______________

Please circle the score that best reflects your assessment for each criterion.

Criterion	1 (Poor)	2 (Suboptimal)	3 (Adequate)	4 (Good)	5 (Excellent)
**1. Resection Completeness**	Major residual adenoma evident. Surgical capsule not visualized.	Significant residual tissue. Capsule partially visualized but inconsistently.	Moderate residual tissue in non-critical areas. Capsule mostly visualized.	Minimal residual tissue. Capsule well visualized circumferentially.	No appreciable residual adenoma. Surgical capsule clearly visualized throughout.
**2. Channel Patency /**	Irregular, narrow channel. Landmarks	Channel patent but asymmetrical or	Functional channel but with some irregularity.	Smooth, wide channel with good anatomical	Ideal, wide, and symmetrical channel

Criterion	1 (Poor)	2 (Suboptimal)	3 (Adequate)	4 (Good)	5 (Excellent)
**Anatomical Shape**	(e.g., verumontanum) damaged.	poorly defined.		contour.	from bladder neck to apex.
**3. Hemostasis**	Significant, ongoing bleeding obscuring view at segment end.	Moderate bleeding requiring repeated coagulation, affecting efficiency.	Controlled bleeding with some persistent oozing.	Good hemostasis; minor oozing easily controlled.	Excellent hemostasis; clear field with minimal to no bleeding.
**4. Recognition of Surgical Landmarks**	Landmarks (verumontanum, bladder neck, capsule) not respected or injured.	Landmarks poorly identified or at risk of injury.	Landmarks identified but resection boundaries occasionally unclear.	Landmarks well-respected, with precise resection boundaries.	Exemplary respect for and preservation of all anatomical landmarks.
**5. Tissue Removal Efficiency**	Fragmented, inefficient resection with many small chips.	Resection technique inconsistent, with some inefficiency.	Adequate technique, but room for improvement in chip size/consistency.	Efficient resection producing good-sized tissue chips.	Highly efficient, systematic resection producing large, uniform chips.

Overall Technical Impression (Please circle one):
1 - 2 - 3 - 4 - 5
(Unacceptable - Borderline - Satisfactory - Proficient - Masterful)

Thank you for your valuable time and expert assessment.
