# Supplementary material for: High-frequency irreversible electroporation versus transurethral resection of the prostate for benign prostatic hyperplasia (GIANT): a single-centre, randomised, double-blind, phase 3, non-inferiority trial
Source: eClinicalMedicine. 2026 Jul 2;97:104034. doi: 10.1016/j.eclinm.2026.104034 (PMC13352031; doi:10.1016/j.eclinm.2026.104034)
Supplement: Summary of changes in protocol [file mmc3.pdf]

Summary of Protocol Amendments (from V1.0 to V3.0)

Protocol Version & Date	Section / Category	Original Text (Previous Version)	Amended Text (Updated Version)
**V1.0 (March-2022)**	Original Protocol	N/A	Initial protocol established.
**V2.0 (April-2022)**	1. Study Design	"...randomised controlled, single-blinded and non-inferiority study..."	"...randomised controlled, double-blinded and non-inferiority study..."
	2. Primary Outcome	"...maximal flow rate (Qmax) at 1, 3, 6, 12 and 24 months after surgical treatment."	"Co-primary outcome: maximal flow rate (Qmax) and urinary symptoms by IPSS scoring at 3 months after surgical treatment."
	3. Secondary Outcomes	Evaluated multiple parameters (IIEF-5, PVRU, Voided volume, ICIQ, QOL, etc.) longitudinally at 1, 3, 6, 12, and 24 months.	Unified all secondary outcome evaluations to focus specifically on the 3-month endpoint.
	4. Statistical Analysis	Non-inferiority based on Qmax alone: lower bound of 95% CI > −4mL/s.	Non-inferiority based on co-primary endpoints: lower bound of 95% CI for Qmax > −4mL/s AND IPSS > 3 points.
	5. Trial Design & Timeframe	Determine non-inferiority "in the short, middle, and long term." (Table 1 timeframe included up to 24 months).	Determine non-inferiority "for achieving better functional outcomes." (Table 1 updated accordingly).
	6. Outcome Definitions	Lacked specific definition for IPSS applicability.	Added strict definition: "IPSS will be assessed for those who have removed the bladder catheter... If patients still have bladder catheters, IPSS will not be assessed."
	7. Follow-up & Long-term Extension	Regular follow-up up to 24 months as a single core trial.	Core trial primary assessment strictly defined at 3 months. Added entirely new section: "Long-term Extension Study" for 6, 12, 24-month data.
	8. Interim Analysis	Planned blinded interim analysis after 70 men reached 3-months follow-up.	Deleted entirely to preserve statistical power and blinding integrity.
	9. Data Collection	Included sections for data collection at 6, 12, 24 months.	Deleted from the core trial protocol (moved to the Long-term Extension phase).
	10. Quality Control (New Sections)	N/A (Lacked dedicated QA sections).	Massive addition of QA sections: Surgical Quality Assurance (H-FIRE & TURP), Randomization QC, Standardized Patient Assessment, Data Management, and Endpoint Adjudication Committee.
	11. Ethics & Dissemination	Standard single publication plan.	Clarified publication strategy: Core manuscript reports 3-month efficacy/safety; explicitly stating long-term outcomes will be reported via the ongoing extension study.
**V3.0 (Jan-2023)**	1. Blinding Methods (Elaboration)	Brief description: Participants and assessors blinded; surgical team excluded from assessment.	Major elaboration on Sham Protocol: Standardized continuous bladder irrigation (based on urine color), standardized catheter removal, unified complication counseling, and mandatory sham perineal dressings for all patients.
	2. Typographical Correction	Sample size section typo: "4.5 ml/s"	Corrected to: "4.5 point" (referring to IPSS).
