# Supplementary material for: High-frequency irreversible electroporation versus transurethral resection of the prostate for benign prostatic hyperplasia (GIANT): a single-centre, randomised, double-blind, phase 3, non-inferiority trial
Source: eClinicalMedicine. 2026 Jul 2;97:104034. doi: 10.1016/j.eclinm.2026.104034 (PMC13352031; doi:10.1016/j.eclinm.2026.104034)
Supplement: GIANT HFire vs TURP SAP V1.1 [file mmc4.pdf]

STATISTICAL ANALYSIS PLAN

Guided Irreversible electroporation Aim to Narrow down Thick prostate

（GIANT）

A parallel, double-blinded, non-inferior randomized controlled study evaluating the efficacy and safety of high-frequency irreversible electroporation in treating lower urinary tract symptoms and benign prostatic obstruction

Chief investigator: Professor Hai-Feng Wang

Co-Chief investigator: Bi-Ming He

Trial Sponsor: Shanghai East Hospital, School of Medicine, Tongji University

Clinical Trial Registration: NCT05306145

SAP Version: INITIAL#1.1 01-Dec-2024

Summary

The objective of this document is to outline the statistical methods and analytical procedures that will guide the data analysis process for this clinical trial. This statistical analysis plan (SAP) is crafted in alignment with the protocol for GIANT trial (**ClinicalTrials.gov: NCT05306145**). The document serves as a commitment to pre-specified analysis plans and a demonstration of the trial's commitment to maintaining the highest standards of scientific rigor and transparency.

It is recognized that the landscape of statistical analysis is dynamic, and advancements may emerge during the course of the study. Should these advancements present more suitable methodologies for the analysis, this SAP is subject to revision to ensure the integrity and robustness of the statistical analysis. Any proposed changes to the SAP will be carefully considered and implemented with strict adherence to the principle of pre-specification. This means that any alternative or additional statistical methods will be clearly defined and adopted before the database is locked, preventing any potential for post-hoc analysis bias. This transparent approach ensures that the analytical process remains valid, reliable, and in compliance with regulatory requirements and reporting guideline for clinical trial reporting.

Changes from Analyses Specified in the Previous Version of the SAP V1.1

The following changes and additions have been made to SAP Version 1.0 (dated 29-Jun-2024):

1. **Sensitivity Analysis (Section 5.3.3.6):** The methodology for the Tipping Point Analysis has been expanded from a general statement to a specific Two-Way Tipping Point Analysis. This includes defining the shift parameters (δ_H and δ_T), the logic for penalizing the H-FIRE group while rewarding the TURP group (and vice versa for IPSS), and the use of heat maps for visualization.
2. **Analysis Population (Section 5.3.3):** Clarified that secondary efficacy analyses will be performed on a Complete Case Analysis basis (all randomized participants with valid baseline and post-treatment data) instead of per-protocol set. This allows for a broader exploratory assessment of secondary outcomes without the strict exclusions of the per-protocol set.

3. **Secondary Endpoints Analysis (Section 5.3.3.2):** Added Time-to-Event analysis for recovery metrics. Specifically, 'Postoperative hospital stay' and 'Catheterization duration' will now be analyzed using Kaplan-Meier curves and Log-Rank tests in addition to the previously specified descriptive statistics and Wilcoxon rank sum tests.

Contents

1. STUDY SUMMARY AND AIMS 1
1.1 Study background 1
1.2 Study Purpose 1
1.3 Study Design and population 1
1.4 Intervention regimens 2
1.5 Randomization and Blindness 3
1.5.1 Randomization 3
1.5.2 Blindness 3
1.6 Follow-up 3
2. STUDY ENDPOINTS 3
2.1 Primary outcome 3
2.2 Secondary outcomes 4
2.3 Safety outcome 5
2.3.1 Definition of Adverse Events 5
2.3.2 Assessment Criteria 5
2.3.3 Recording of Adverse Events 5
3. SAMPLE SIZE CONSIDERATIONS 6
4. ANALYSIS POPULATION 7
4.1 Full Analysis Set (FAS) 7
4.2 Per Protocol Set (PPS) 7
4.3 Safety Analysis Set (SS) 7
5. STATISTICAL CONSIDERATIONS 8
5.1 Time point and statistical software 8
5.2 Disposition of Subjects 8
5.3 Statistical Considerations 9
5.3.1 Basic Characteristic and descriptive statistics 9
5.3.2 Primary analysis on efficacy 10
5.3.3 Secondary endpoints 14
5.3.4 Safety analysis 16

5.3.5 Interim Analysis 17
6 Supporting documentation 17
6.1 Trial documents 17
6.2 Assessment tools 17
6.3 Other references 17

1. STUDY SUMMARY AND AIMS

1.1 Study background

Benign prostatic hyperplasia (BPH) is prevalent in men over 50 and is commonly associated with lower urinary tract symptoms (LUTS) and benign prostatic obstruction (BPO), significantly affecting quality of life and posing an economic burden. Transurethral resection of the prostate (TURP) has been the gold standard treatment, improving outcomes such as maximal flow rate (Qmax) and the International Prostate Symptom Score (IPSS). However, TURP can lead to complications such as transurethral resection syndrome, massive bleeding, urinary incontinence, and sexual dysfunction.

Irreversible electroporation (IRE) is a non-thermal ablation technique that uses pulsed, high voltage direct electric current to induce cell death while minimizing damage to connective tissues. A variant, high-frequency irreversible electroporation (H-FIRE), uses bipolar pulse bursts to reduce muscle contractions during the procedure. In our multicenter single-arm objective performance criteria trial, H-FIRE was used to treat localized prostate cancer and showed significant improvement in the functional outcomes of LUTS/BPO, with fewer reported complications, especially concerning erectile function and urinary continence. Although primarily used for malignant tumors, H-FIRE has shown potential in treating benign conditions like BPH.

Based on this evidence, we hypothesize that H-FIRE will provide similar improvements in functional outcomes as TURP but with fewer side effects. To test this hypothesis, we are conducting a ‘Guided Irreversible electroporation Aim to Narrow down Thick prostate’ (GIANT) trial to evaluate whether H-FIRE is non-inferior to TURP in improving functional outcomes for patients with symptomatic BPH.

1.2 Study Purpose

The purpose of the GIANT trial is to evaluate whether high-frequency irreversible electroporation (H-FIRE) is non-inferior to Transurethral resection of the prostate (TURP) in treating BPH with symptomatic LUTS/BPO. The study aims to determine if H-FIRE can achieve similar or better functional outcomes with fewer side effects compared to the standard TURP procedure, thereby offering a potentially safe and effective treatment option for men with symptomatic BPH.

1.3 Study Design and population

The GIANT study is designed as a prospective, investigator-initiated, single-center, randomized controlled, double-blinded, and non-inferiority trial.

Eligible Subjects: Men with symptomatic BPH, including lower urinary tract symptoms and benign prostatic obstruction (LUTS/BPO).

Inclusion criteria:

- Age over 40 years' old
- International Prostate Symptom Score (IPSS) > 8.
- Maximum urinary flow rate (Qmax) < 15 ml/s.
- Prostatic volume between 30 to 100 ml, measured by MRI.
- Fully understand the clinical trial protocol and sign the informed consent.

Exclusion criteria:

- History of prostate cancer or suspicion of prostate cancer.
- Neurogenic bladder.
- Metal implants in the body.
- Previous history of prostatic or urethral surgery.
- Catheterization for more than 2 weeks.
- Any other conditions that make the investigator judge that participants are not suitable for this trial.

1.4 Intervention regimens

Arm 1: High-frequency irreversible electroporation (H-FIRE)

- **Procedure**: Participants in the H-FIRE arm will receive H-FIRE using equipment manufactured by REMEDINE. The procedure is performed under general anesthesia with full-muscle paralysis and transrectal ultrasound guidance.
- **Electrode Placement**: The electrode needle will be placed perineally, targeting the middle lobe of the prostate through a 5 mm brachytherapy template. Four to six needles will be used, with the distance between adjacent needles varying between 0.5-2.0 cm based on prostate size.
- **Pulse Delivery**: After electrode placement, the apparatus will release pre-set pulses. The entire H-FIRE procedure is expected to last 40-60 minutes.
- **Post-Procedure**: A three-way Foley catheter will be indwelled at the end of the procedure.

Arm 2: Transurethral resection of the prostate (TURP)

- **Procedure**: The TURP procedure will be performed using electrodes and a standard tungsten cutting wire loop at specific cutting and coagulating currents under general anesthesia.
- **Resection**: The resection begins at the 6 o'clock position of the bladder neck, extending to the verumontanum, and down to the surgical capsule, focusing on tissue removal from the

gland's center zone and transition zone.

- **Post-Procedure**: A three-way Foley catheter will be inserted after the procedure.

1.5 Randomization and Blindness

1.5.1 Randomization

Only subjects who have provided informed consent, meet all inclusion criteria and none of the exclusion criteria, will be eligible for randomization. Eligible participants will be randomized in 1:1 ratio to either the H-FIRE arm or the TURP arm using **stratified block randomization**, which is stratified by age (< 70 years, >=70 years) and prostate volume (< 60 ml, >=60 ml).

After an independent nurse checks the patient's informed consent and verifies the inclusion and exclusion criteria, an individual randomization number will be revealed for the participant and will be recorded in the case report form (CRF). Allocation to either group will be concealed until the point of treatment assignment. Randomization numbers will serve as a permanent and confidential identifier for each participant and will be utilized for all subsequent procedures and analyses.

1.5.2 Blindness

All participants will be blinded to the treatment allocation to reduce bias in assessing outcomes, especially patient-reported outcomes (PROs).

The researcher performing the surgical procedure will not participate in subsequent assessments, data collection, or statistical analysis.

Researchers responsible for assessment and statistical analysis will also be blinded to the treatment allocation.

1.6 Follow-up

Regular follow-up will begin one month after the surgical procedure and continue up to 3 months.

2. STUDY ENDPOINTS

2.1 Primary outcome

The GIANT study's primary outcome is a **co-primary endpoint**, which includes two measures assessed at 3 months after surgical treatment:

- **Change in Qmax from Baseline to 3 months**: Qmax is a clinical measure of urodynamics, indicating the maximum speed at which urine is expelled from the bladder. High score indicates less severity of benign prostatic obstruction.

$$\text{Change in Qmax} = \text{Qmax at 3 moths} - \text{Qmax at baseline}$$

- **Change in IPSS from Baseline to 3 months**: IPSS is a validated patient-reported outcome measure that assesses the degree of urinary symptoms over the last 4 weeks. The score ranges from 0 to 35, with higher scores indicating more severe symptoms.

$$\text{Change in IPSS} = \text{IPSS at 3 moths} - \text{IPSS at baseline}$$

2.2 Secondary outcomes

The secondary outcomes of the GIANT study encompass a range of assessments at 3 months' post-treatment, including:

1. **Change at 3 months from baseline in Erectile Symptoms**:
   a) 5-item version of the International Index of Erectile Function (**IIEF-5**): IIEF-5 is a self-report questionnaire to the presence and severity of erectile dysfunction, ranging from 0 to 25, with higher scores indicating better erectile function.
   b) International Consultation on Incontinence Questionnaire Male Sexual Matters Associated with Lower Urinary Tract Symptoms Module (**ICIQ-MLUTSsex**). The ICIQ-MLUTSsex is 4-item self-report questionnaire for detailed evaluation of male sexual matters associated with their lower urinary tract symptoms and impact on quality of life (QoL). It is 0-12 overall score with greater values indicating increasing problems with sexual matters.
2. **Change at 3 months from baseline in Post-Void Residual Urine Volume (PVRU)**: PVRU is the amount of urine retained in the bladder after a voluntary void and is a clinical measure for assessing BPO.
3. **Change at 3 months from baseline in Voided Volume**: Voided volume is the amount of urine that is a voluntary void and functions as a clinical measure for assessing LUTS.
4. **Urinary Incontinence**: Assessed by the ICIQ score (**Change at 3 months from baseline**) and the Expanded Prostate Cancer Index Composite (EPIC) pad-use item (**at 3 months**).
   a) ICIQ score is a 3-item self-report questionnaire for urinary incontinence, ranging from 0 to 21, with higher scores indicating worse incontinence.
   b) EPIC pad-use is a 1-item self-report questionnaire to record the number of pad use daily.
5. **Change at 3 months from baseline in Quality of Life (QOL)**:
   a) IPSS QoL subscore is a 1-item self-report questionnaire to assess the quality of life with prostate symptoms, ranging from 0 to 6, with higher scores indicating a worse quality of life.
   b) Hospital Anxiety and Depression Scale (HADS) is a self-assessment scale for evaluating the presence and severity of anxiety and depression, ranging from 0 to 42, with a higher score indicating worse distress.

6. **Change at 3 months from baseline** in **Pain assessment**: Surgical Pain Scale is a 4-item self-report questionnaire, ranging from 0 to 40, with a higher score indicating worse pain.
7. **Perioperative outcomes**:
   a) Operative time, hour, the time of the procedure of H-FIRE or TURP
   b) Postoperative hospital stay, day, the length from the first day after surgery to discharge
   c) Change in hemoglobin from baseline to post-operation at 6 hours and 24 hours
   d) Change in serum sodium from baseline to post-operation at 6 hours and 24 hours
   e) Catheterization duration, day, the length from the first day after surgery to de-catheterization
8. **Early Postoperative urinary symptoms at 3 months**: Early Postoperative Urinary Symptoms assessment is a 5-item self-report questionnaire to test severity of blood in the urine, difficulty in urination or urinary retention, painful during passing urine, painful after passing urine and urine urgency.

2.3 Safety outcome

The safety assessment will be made by independent Data Safety Monitoring Board (DSMB) blinded to assignment group from the beginning of the trial to 24 months after the surgical procedure.

2.3.1 Definition of Adverse Events

An Adverse Event (AE) is defined as any untoward medical occurrence in a research participant that happens during the conduct of a clinical trial. These events may or may not be related to the biopsy procedure and are captured regardless of causality.

2.3.2 Assessment Criteria

All AEs will be assessed using the Common Terminology Criteria for Adverse Events (CTCAE), which provides a standardized nomenclature and grading system for the evaluation of AEs.

2.3.3 Recording of Adverse Events

The following AEs, relevant to the biopsy procedures and potential complications, will be specifically recorded and monitored throughout the study:

- Hematuria
- Hematuria requiring hemoglobin measure
- Hematuria requiring transfusion
- Infection of urine
- Fever
- Clot retention
- Prostatic tissue retention
- Urinary retention

- Urethral stricture
- Urinary incontinence
- Erectile dysfunction
- Hematospermia
- Lower urinary tract symptoms
- Discomfort when passing urine
- Intra-perineal discomfort
- others

A serious adverse reaction as determined by the PI is any event that results in any of the following outcomes:

- Death
- Life-threatening
- Need to hospitalization or prolonging an existing hospitalization
- Disability or permanent damage

3. SAMPLE SIZE CONSIDERATIONS

The sample size is calculated to detect a non-inferiority margin for the co-primary endpoints, which are the change at 3 months after surgical treatment from baseline in Qmax and IPSS.
A non-inferiority margin is predetermined for both change in Qmax (larger is better) and change in IPSS (smaller is better). For Qmax, the margin is -4 mL/s, and for IPSS, it is 3 points. These margins represent the smallest differences in outcomes that would be considered clinically significant. It is assumed that changes in Qmax and IPSS are equal for both treatment groups (H-FIRE and TURP). Parameters for the sample size estimation are summarized in Table 1 below. Using an allocation ratio of 1:1, 49 men per arm will be required. Accounting for a 20% withdrawal/loss rate, a total of 118 participants are required for inclusion.

Table 1 parameters in sample size estimation

Outcome	Alpha (one-sided)	Targeted power	Assumed true difference	Standard deviation	Margin	sample size per arm
Change in Qmax, ml/L, larger is better	0.025	0.9	0	6	-4	49
Change in IPSS, Point, smaller is better	0.025	0.9	0	4.5	3	49

4. ANALYSIS POPULATION

Subjects are considered enrolled at the time they sign informed consent. Subjects will be included in analysis populations when they have provided consent and have been randomized to a surgery arm. The reasons that screen failure will be summarized as a list.

4.1 Full Analysis Set (FAS)

The Full Analysis Set (FAS) will comprise all participants who are randomized to either the H-FIRE or TURP treatment groups to test EFFICACY, regardless of whether they complete the study as intended or experience protocol deviations.

Subjects within the FAS will be analyzed according to randomized treatment assignment, known as the "intention-to-treat" (ITT) principle. The FAS population will be used for primary analysis of primary outcome to test non-inferiority.

The final clinical report will include a detailed description of any subjects or observations that were excluded from the FAS, along with the rationale for their exclusion.

4.2 Per Protocol Set (PPS)

The Per-Protocol (PP) Set is a subset of the FAS population. The PP Set includes only those participants who complete the study according to the protocol, without major deviations. Protocol deviations will be classified in the following categories:

- Dose not fulfill eligibility criteria.
- Not adhered to randomized and protocol-required procedure.
- Missing data in basic characteristics and primary outcome.
- Others

The PP population will be used for sensitivity analysis of primary endpoints.. A list of patients to be excluded from the randomized patients to create the PP analysis will be established and validated by the DSMB prior to database lock.

4.3 Safety Analysis Set (SS)

The Safety analysis set includes all subjects all patients who received any planned surgery (H-FIRE or TURP). In case of violation of the randomization scheme, patients will be classified according to the treatment they actually received.

This set will be used to summarize safety outcomes.

5. STATISTICAL CONSIDERATIONS

5.1 Time point and statistical software

The designated and authorized statistician(s) will be fully responsible for conducting the statistical analysis and preparing the statistical report of the study. The statistician will operate independently to ensure the integrity and objectivity of the analysis. All statistical analyses will be performed on blinded data. The database will be locked prior to the final analysis, ensuring that no further changes can be made to the data, which supports the integrity of the statistical analysis.

Any comments on the pre-final analysis will be addressed and resolved in a timely manner. Only after all feedback has been satisfactorily incorporated and the study database is declared final will the final analysis proceed. The last version of the SAP will be finalized and signed off by the responsible parties before the database lock and the commencement of the final analysis. All statistical calculations will be performed using SAS software, version 9.4 (TS level 1M6, Windows x64_10).

5.2 Disposition of Subjects

All efforts will be made to collect outcome data also in patients withdrawn from the trial for whichever reasons and to minimize the amount of missing data. The process by which an independent team of **clinical research associate (CRA) and a DSMB** monitor participant flow, ensuring adherence to the protocol and ethical guidelines.

A comprehensive summary of participant flow through the study will be documented and tabulated to ensure transparency and facilitate the understanding of the study's progress and outcomes. The following information will be included:

- Number of patients screened and reasons for screen failure.
- Number of participants who meet the inclusion and exclusion criteria and provide informed consent.
- Number of participants randomly allocated to treatment groups (H-FIRE or TURP).
- Number of participants who complete the scheduled follow-up visits and assessments.
- Number of patients in the full analysis set and number of patients excluded from this set along with reasons.
- Number of patients in the per-protocol set and number of patients excluded from this set along with reasons.
- Number of patients in the safety set and number of patients excluded from this set along with reasons.
- Number of patients who withdraw from the study for various reasons, including personal

choice, non-compliance, adverse events, or other protocol-specified criteria.

The study report and publications will include a **participant flow diagram** that visually represents the disposition of subjects throughout the study, typically in the form of a CONSORT (Consolidated Standards of Reporting Trials) diagram.

5.3 Statistical Considerations

Baseline characteristics will be collected prior to the randomization and include demographic and clinical parameters relevant to the study. These will be recorded in the Case Report Forms (CRFs) for each enrolled participant. Tabular and figure summaries will be presented based on the intervention groups.

5.3.1 Basic Characteristic and descriptive statistics

The following basic characteristics will be coded and summarized:

- Age, year
- Ethnic group
- Body Mass Index, kg/m^2
- Medical history:
- Co-medications for prostate: No, 5α-reductase inhibitors, α1-adrenergic receptor antagonists, Combination of both drugs, Others.
- Prostate-Specific Antigen (PSA), ng/ml
- Prostate volume (measured by MRI), ml
- DRE: negative, positive
- Bladder stone: yes/no
- Catheterization Status: yes/no

The following clinical assessment at baseline will be summarized:

- Maximal Flow Rate (Qmax), ml/s
- International Prostate Symptom Score (IPSS), scale, point
- 5-item version of the International Index of Erectile Function (IIEF-5), scale, point
- International Consultation on Incontinence Questionnaire Male Sexual Matters Associated with Lower Urinary Tract Symptoms Module (ICIQ-MLUTSsex), scale, point
- Post-Void Residual Urine Volume (PVRU), ml
- Voided Volume, ml
- International Consultation on Incontinence Questionnaire (ICIQ) score, scale, point
- Expanded Prostate Cancer Index Composite (EPIC) pad-use item, scale, point
- Quality of Life (QOL) assessed by IPSS QoL subscore, scale, point
- Hospital Anxiety and Depression Scale (HADS), scale, point

- Hematology assessments, including haemoglobin, serum sodium levels, et, al.

Categorical variables were described using frequencies and percentages, continuous variables were described as means +/- standard deviation for normally distributed variables or medians (the first quartile, the third quartile) for non-normal variables. Between-group comparisons are evaluated using Fisher exact test, Student's t test and Wilcoxon rank sum test, respectively.

5.3.2 Primary analysis on efficacy

5.3.2.1 clarification of selection of analysis set

Traditionally, primary analysis of a clinical trial follows the ITT principle, namely, all randomized patients are analyzed according to the treatment to which they were randomized, including patients who leave the study prematurely. Protocol violations, such as non-adherence, misclassification of the primary endpoint, or attrition, tend to bias the results toward no treatment difference (success) and undermine the validity of the trial, creating apparent non-inferiority. In accordance with guidance provided by regulatory authorities (Chinese National Medical Products Administration and U.S. Food and Drug Administration), **it is recommended to simultaneously perform analysis on two analysis sets for non-inferiority trials: the intention-to-treat (ITT) set, which represents an ideal scenario, and the per-protocol set (PPS), which reflects real-world conditions more closely.**

Consistency in conclusions drawn from both analysis sets indicates robust quality control and reliable study results. However, if conclusions from the two sets are inconsistent, it may indicate issues related to protocol adherence, effect modifiers, or other factors that could influence the outcome. In such cases, further discussion, sensitivity analyses, and in-depth exploration of the data are warranted to understand the reasons for the discrepancies and to provide a more nuanced interpretation of the study results.

Even though the statistical strategy of using both FAS and PP is adopted, study quality at the planning stage is emphasized and the trial during the conduct and analysis stages are continuously monitor to minimize the potential bias.

5.3.3.2 Hypothesis

The study adopts co-primary endpoints, including change in Qmax and IPSS from baseline to 3 months. The primary hypothesis to be tested is that H-FIRE is non-inferior to TURP in FAS and PPS.

Change in Qmax from baseline to 3 months (larger is better)

The non-inferiority margin is 4 mL/s for Qmax, let D_1 denotes the difference in Change in Qmax from baseline to 3 months:

$\mathbf{D_1}$ = mean change in Qmax in H-FIRE group – mean change in Qmax in TURP group

The null-hypothesis and alternative hypothesis of non-inferiority are formally expressed as:

$$H_0 : D_1 \leq -4$$

$$H_a : D_1 > -4$$

Change in IPSS from baseline to 3 months (smaller is better)

The non-inferiority margin is 3 points for IPSS, let D_2 denotes the difference in Change in IPSS from baseline to 3 months:

$\mathbf{D_2}$ = mean change in IPSS in H-FIRE group – mean change in IPSS in TURP group

The null-hypothesis and alternative hypothesis of non-inferiority are formally expressed as:

$$H_0 : D_2 \geq 3$$

$$H_a : D_2 < 3$$

The study can declare success only if both primary endpoints statistically meet non-inferiority. There is no risk from multiplicity since co-primary endpoints are used in this study. Each primary endpoint is tested at significant level of one-sided 0.025. Non-inferiority can be confirmed if the lower bound of the two-sided 95% confidence interval (CI) of the difference for change in Qmax is greater than -4 ml/s, and the upper bound of the two-sided 95% CI of the difference for change in IPSS is less than 3 points, equating to a one-sided P-value threshold of less than 0.025.

5.3.3.3 Statistical methods

In this single-center trial, multiple linear models are used to estimate potential differences in the primary endpoints between the two groups. For each model on either co-primary outcome, the change from baseline is modeled as the dependent variable, and the group variable is included as the independent variable of interest. Additionally, the baseline value and two stratification factors (age group: < 70 years, >=70 years; prostate volume: < 60 ml, >=60 ml) are included as covariates. The least squares mean change and corresponding 95% confidence interval (CI) will be estimated for each group. Importantly, the difference in changes between the two groups will be estimated with a two-sided 95% CI.

Stratification characteristics will be coded and included as fixed covariates:

- Age (< 70 years, >=70 years)
- Prostate volume (< 60 ml, >=60 ml).

Example SAS code is shown below:

```
proc mixed data=final;
    class group age_group PV_group;
    model change_from_baseline= group baseline_value age_group PV_group;
    lsmeans group /DIFF CL alpha=0.05; * 95% CI;
run;
```

5.3.3.4 Handling of missing value

Missing data is a common occurrence in clinical research. All efforts will be made to obtain complete information on baseline characteristics, outcomes, and corresponding dates. The study site will be asked to ensure that all data elements are complete, regardless of whether the subject was treated with the study intervention. The reasons for early discontinuation and dropouts will be summarized by group to assess potential biases in the analyses due to differences between groups.

Reporting missing information is crucial for ensuring the accuracy, reliability, and validity of the study results. This allows researchers to accurately analyze the data and draw valid conclusions about the safety and efficacy of the intervention being studied. Initially, the missingness pattern for each co-primary endpoint will be reported. Subsequently, model-based multiple imputation by chained equations (MICE) will be used to impute the missing values. If the missing rate is less than 20%, ten imputed datasets will be generated. If the rate is greater than 20%, twenty imputed datasets will be generated to ensure that our effect estimates are not unduly influenced by Monte Carlo variability.

Example SAS code is shown below:

```
proc mi data= final out=inputed_DS nimpute=10 seed=2024;
    by group; /* separate for different surgery group */
    class group age_group PV_group;
    fcs regpredmeanmatch; /* regression for continuous variable */
    /* include both primary endpoints*/
    var group age_group PV_group Qmax0 IPSS0 Qmax1 IPSS1;
run;
data inputed_DS;
    set inputed_DS;
    change_from_baseline= IPSS1 - IPSS0; * update change;
run;
proc sort data=inputed_DS;
    by _imputation_;
run;
ods output diffs=diffs;
proc mixed data= inputed_DS;
```

```
    by _imputation_;
    class group age_group PV_group;
    model change_from_baseline = IPSS0 group age_group PV_group;
    lsmeans group/pdiff CL alpha=0.05;
run;
proc mianalyze data=diffs alpha=0.05;/* pool and give 95% confidence interval*/
    modeleffects estimate;
    stderr stderr;
run;
```

5.3.3.5 Pre-specified subgroup Analysis

The purpose of the pre-specified subgroup analysis is to explore whether the treatment effects of H-FIRE versus TURP are consistent across various subgroups of participants. This analysis will help identify specific populations where the treatment might be more or less effective or have different safety profiles. Heterogeneity tests will be performed to evaluate whether there is substantial evidence indicating that the effects in specific subgroups differ significantly from the overall effect observed in all patients combined. To explore potential effect modification, an interaction term between the surgery group and the subgroup factor will be created and introduced into the regression model.

Subgroup analyses will be performed on FAS and PP population. Subgroups to be examined include:

- Age: <70 years, >=70 years
- BMI: <24, >=24
- Medical Treatment of LUTS before Surgery: No drug treatment, 5α-reductase inhibitors, α1-adrenergic receptor antagonists, Combination of both drugs
- Prostate volume (< 60 ml, >=60 ml).
- Catheterization Status before Surgery: Yes, no
- Bladder Stone Status before Surgery: Yes, no

The results of subgroup analyses will be presented in a forest plot, providing information on the number of patients in each subgroup, the number of patients in each treatment group, the adjusted absolute difference, and the corresponding 90% CI and P-value for interaction.

5.3.3.6 Sensitivity analysis

Sensitivity analyses play a crucial role in assessing the robustness of the findings or conclusions based on primary analyses of data in clinical trials. The following sensitivity analyses of the co-primary endpoints will be performed:

- **Post hoc baseline adjustment:** A pronounced baseline imbalance is not expected a priori in a

randomized trial if the randomization process has worked correctly. However, if a significant imbalance is observed in group comparisons of basic characteristics (two sided P value less than 0.05), the imbalanced baseline characteristics will be further adjusted for as part of a sensitivity analysis.

- **Tipping Point Analysis**: To assess the robustness of the primary efficacy results against deviations from the Missing At Random (MAR) assumption, a two-way tipping point analysis will be conducted under the Missing Not At Random (MNAR) assumption. This analysis assumes that the missing outcomes in the H-FIRE and TURP groups may differ systematically from the observed outcomes by specific shift parameters, denoted as δ_H and δ_T, respectively. We will vary the shift parameters independently over a range of plausible values to identify the "tipping point". The adjusted datasets will be analyzed using the primary Mixed Model (LMM), and results will be pooled using Rubin's rules. The results will be presented using a heat map. The x-axis and y-axis will represent the shift parameters for the H-FIRE and TURP groups, respectively. The region where non-inferiority is maintained will be distinguished from the region where it is not, allowing for an assessment of whether the tipping point falls within a clinically likely range of missing data bias.

5.3.3 Secondary endpoints

The analysis of secondary outcomes is intended to provide supportive evidence for the primary efficacy findings. Secondary efficacy analyses will be performed on **all randomized participants who have valid baseline and post-treatment data for the respective outcome** (Complete Case Analysis). Findings from secondary outcomes will be interpreted as exploratory to avoid potential multiplicity. 95% confidence intervals and P values will not be adjusted for multiple outcomes. Missing data will not be imputed for secondary outcomes, but corresponding missingness patterns will be reported.

Statistical methods will be selected based on the data type and distribution characteristics of each outcome variable:

5.3.3.1 Continuous Variables (Linear Mixed Models)

For secondary endpoints that are continuous and assumed to be approximately normally distributed, a linear mixed model will be used. The model will include the change from baseline as the dependent variable, with the treatment group, age group, prostate volume group, and baseline value as fixed effects. The least squares mean difference between groups (H-FIRE minus TURP) and its 95% CI will be estimated. Outcomes in this category include:

- 1(1) Change at 3 months from baseline in Erectile Symptoms using IIEF-5.
- 1(2) Change at 3 months from baseline in Erectile Symptoms using ICIQ-MLUTSsex.
- 3 Change at 3 months from baseline in Voided Volume. Note: Change in voided volume is

considered a physiological measure of functional bladder capacity, where changes are expected to approximate a normal distribution, thereby justifying the use of parametric linear mixed models with covariate adjustment.

- 4(1) Change at 3 months from baseline in Urinary Incontinence using ICIQ score.
- 5(2) Change at 3 months from baseline in Quality of Life using HADS. Note: anxiety and depression will be analyzed separately.
- 7(c) Change in hemoglobin from baseline to post-operation at 6 hours and 24 hours.
- 7(d) Change in serum sodium from baseline to post-operation at 6 hours and 24 hours.

Example SAS for is shown below:

```
/* Linear Mixed Model (For Items 1.1, 1.2, 3, 4.1, 5.2, 7.c, 7.d) */
proc mixed data=final;
    class group age_group PV_group;
    model Change_from_baseline = group baseline_value age_group PV_group;
    lsmeans group /DIFF CL alpha=0.05; * 95% CI;
run;
```

5.3.3.2 Skewed, Ordinal, and Count Variables (Non-Parametric Tests)

For variables that typically exhibit a skewed distribution, linear models are inappropriate. Due to the exploratory nature of these analyses and methodological constraints in adjusting medians, covariates will not be adjusted to provide a direct and robust comparison of the randomized groups.

Median and 95% CI for each group will be reported. The Median difference (Hodges-Lehmann estimate) and 95% CI will be estimated. Between-group comparisons will be performed using the Wilcoxon Rank Sum test. Outcomes in this category include:

- **2** Change at 3 months from baseline in Post-Void Residual Urine Volume (PVRU). Note: Change in PVRU typically exhibits a marked non-normal, right-skewed distribution characterized by a 'floor effect' (clustering of values near zero or the limit of detection) and extreme outliers. Consequently, it is analyzed using robust non-parametric methods based on ranks.
- **4(2)** Urinary Incontinence using EPIC pad-use at 3 months. Note: Additionally, the proportion of "Pad-Free" patients (score = 0) may be compared using Fisher’s exact test.
- **5(1)** Change at 3 months from baseline in Quality of Life using IPSS QoL subscore.
- **6** Surgical pain assessment at 3 months (Surgical Pain Scale items, range: 0-10). As surgical pain is not expected at baseline, the post-operative score is the relevant outcome. Note: Due to the conditional nature of Item 3 ("pain during exercise"), a total sum score will not be calculated. The 4 items will be analyzed separately. For Item 3, responses indicating "I have not done any of these activities" will be treated as missing data (not imputed as 0), and the

analysis will be conducted on the sub-population of patients who performed the activities.

- **7(a)** Operative time (hour).
- **7(b)** Postoperative hospital stay (day).
- **7(e)** Catheterization duration (day).
- **8** Early Postoperative urinary symptoms at 3 months. This scale assesses symptoms specifically arising from the surgery, thus no baseline value exists.

Example SAS for is shown below:

```
proc univariate data=final cipctldf;
    class group;
    var EPIC_pad_use IPSS_QoL;
    ods select Quantiles;
run;
/* Non-parametric test with Hodges-Lehmann estimation (HL) */
proc npar1way data=final Wilcoxon HL;
    class group;
    var EPIC_pad_use IPSS_QoL;
run;
```

For recovery metrics, in addition to the descriptive summary statistics (medians and IQR) and Wilcoxon rank sum tests, the Postoperative hospital stay (Item 7b) and Catheterization duration (Item 7e) will be further analyzed using time-to-event methods to visualize and compare the **speed of recovery** between the two groups. For these analyses, the "event" is defined as hospital discharge or catheter removal, respectively. Kaplan-Meier curves will be plotted to estimate the distribution of time to discharge and time to catheter removal. The Log-Rank test will be used to compare the survival curves between the H-FIRE and TURP groups. This approach provides a dynamic view of the recovery trajectory that complements the median comparisons.

5.3.4 Safety analysis

Safety analysis will be performed using the safety population. Missing data will not be imputed. Adverse events occurring in patients from the Safety set will be presented in data listings. All outputs will be summarized separately by the two biopsy groups, which will present the number and percentage of patients (crude incidence rate).

For all treated subjects, summary tables will be provided for each of the following categories:

• Any adverse events (overall and by maximum severity)
• Any adverse events of special interest (overall and by maximum severity)
• Any serious adverse events (SAEs)

Results for safety outcomes will be presented with count and incidence rate (%), which will be compared between the two groups using of Chi-squared test or Fisher's exact test, as appropriate.

5.3.5 Interim Analysis

This clinical trial is designed as a single-center study with a follow-up period of 3 months. Given the nature of the intervention and the expected outcomes, we have determined that a formal interim analysis is not necessary for the integrity and validity of the study results. However, to ensure the safety of participants and the integrity of the trial, we have established a DSMB. Should the DSMB determine that an unplanned interim analysis is warranted, an independent statistician will be invited to join the DSMB to conduct this analysis.

The results of any such interim analysis will be treated with the utmost confidentiality. The DSMB will review the findings and provide recommendations regarding the continuation, modification, or termination of the trial. The final decision on any changes to the trial protocol will be made by the trial's steering committee, taking into account the DSMB's recommendations and adhering to ethical and regulatory standards.

6 Supporting documentation

6.1 Trial documents

Clinical Trial Registration: www.ClinicalTrials.gov, identifier: **NCT05306145**.

This trial is supported by National key research and development program(2019YFC0119100) from National Natural Science Foundation of China and supported by Shanghai Science and Technology Commission Foundation (18441910900) from Science and Technology Commission of Shanghai Municipality.

The study sponsor or funder had no authority over study design, management, execution, data collection, analysis, manuscript writing, outcome disclosing, and dissemination or publishing.

6.2 Assessment tools

All assessment tools are available in the appendices of protocol version 1.2.

6.3 Other references

[1] Lourenco T, Pickard R, Vale L, et al. Minimally invasive treatments for benign prostatic enlargement: systematic review of randomised controlled trials. BMJ. 2008;337:a1662.

[2] Food and Drug Administration. Non-Inferiority Clinical Trials to Establish Effectivenes

s, Guidance for Industry. Available from: https://www.fda.gov/regulatory-information/search-fda-guidance-documents/non-inferiority-clinical-trials. Access at May 25, 2024.

[3] Center for drug evaluation, NMPA. Guiding principles for non-inferiority design of drug clinical trials. Available from:https://www.cde.org.cn/main/news/viewInfoCommon/322593ac8e690e63730fc63acd1ecba4. Access at May 25, 2024.

[4] Kevin S. Kim, An-Wen Chan, Emilie P. Belley-Côté, et al. Noninferiority Randomized Controlled Trials. Journal of Investigative Dermatology. 2022; 142 (7):1773-1777.

[5] Cuzick, J., Sasieni, P. Interpreting the results of noninferiority trials—a review. Br J Cancer. 2022; 127:1755–17

[6] McLeod C, Norman R, Litton E, Saville BR, Webb S, Snelling TL. Choosing primary endpoints for clinical trials of health care interventions. Contemp Clin Trials Commun. 2019;16:100486.
